# Outcomes of point-of-care manufactured CAR T cell therapy for B cell acute lymphoblastic leukemia and non-Hodgkin lymphoma in Vietnam

**DOI:** 10.1016/j.omton.2026.201156

**Published:** 2026-02-13

**Authors:** Liem Thanh Nguyen, Duy D. Nguyen, Quoc Khanh Bach, Lan T.M. Dao, Trang Thi Kieu Phan, Hoang - Phuong Nguyen, Hong-Nhung Dao, Trang H. Pham, Phuong T. Pham, Hien T. Mai, Viet Huong T. Pham, Thanh Mai T. Nguyen, Van Binh Le, Nam Lam Phung, Ngoc Quang Nguyen, Michelle L. Hermiston, Quynh Lan Phan, Do Quang Trung Nguyen, Lan Mai, Quoc Nhat Nguyen, Van T. Hoang

**Affiliations:** 1 Vinmec Research Institute of Stem Cell and Gene Technology, VinUniversity, Hanoi, Vietnam; 2 Department of Regenerative Medicine and Cell Therapy, Vinmec Healthcare System, Hanoi, Vietnam; 3 Vinmec Smart City International Hospital, Vinmec Health Care System, 2A Tay Mo Street, Hanoi 12009, Vietnam; 4 National Institute of Hematology and Blood Transfusion (NIHBT), 5 Pham Van Bach, Cau Giay District, Hanoi, Vietnam; 5 Vinmec Times City International Hospital, Vinmec Health Care System, 458 Minh Khai, Hanoi 11622, Vietnam; 6 College of Health Sciences, VinUniversity, Vinhomes Ocean Park, Gia Lam District, Hanoi 1310, Vietnam; 7 University of California, San Francisco, Helen Diller Comprehensive Cancer Center, 1450 3^rd^ Streen, San Francisco, CA, USA

**Keywords:** acute lymphoblastic leukemia, non-Hodgkin lymphoma, CAR T-cell therapy, point-of-care manufacturing, CRS, ICANS, resource-constrained settings, cell therapy, global health oncology

## Abstract

Chimeric antigen receptor (CAR) T cell therapy has transformed the treatment of relapsed/refractory (R/R) B cell acute lymphoblastic leukemia (ALL) and non-Hodgkin lymphoma (NHL), but access remains limited in resource-constrained settings. This phase I study evaluated the safety and feasibility of point-of-care (PoC) manufactured CD19-targeted CAR T cell therapy in Vietnamese patients. Between August 2023 and June 2025, 16 patients, eight with R/R ALL and eight with R/R NHL, were enrolled. All received fresh CAR T cells produced on-site using the CliniMACS Prodigy system, with a median dose of 1.9 × 10^6^ CAR T cells/kg (range, 0.83–2.17 × 10^6^). Cytokine release syndrome (CRS) occurred in 13 patients (12 with grade 1–2, one with grade 3), and grade 1 neurotoxicity was observed in two patients. In ALL, the complete remission (CR) rates were 100% on day 30, 75% on day 90, and 62.5% on day 180. Patients with NHL showed CR rates of 87.5% on both day 90 and day 180. The estimated 1-year progression-free survival rates were 62.5% (95% confidence interval [CI]: 36.5%–100%) for ALL and 87.5% (95% CI: 67.3%–100%) for NHL. PoC manufactured CD19 CAR T cells demonstrated manageable toxicity and encouraging early efficacy in Vietnamese patients with R/R ALL and NHL. This model offers a cost-effective strategy for delivering advanced therapy in resource-limited settings.

## Graphical abstract

**Figure:**
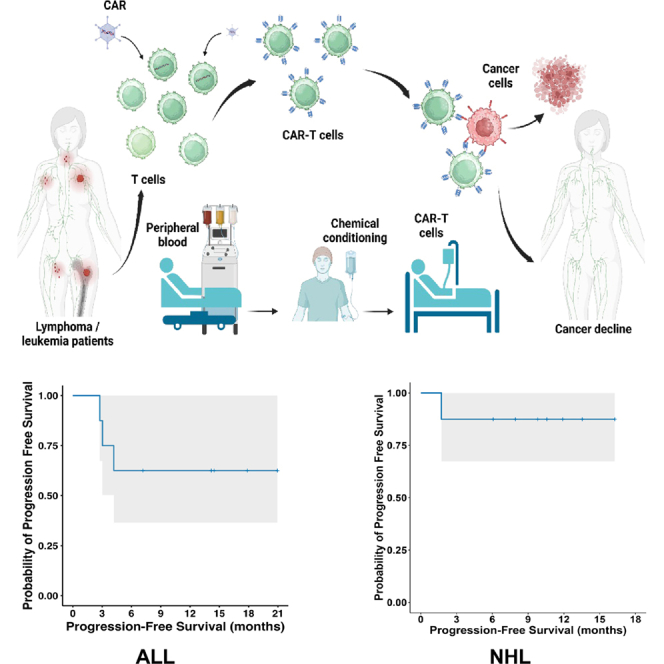

## Teaser

This phase 1 clinical trial in Vietnam demonstrates that point-of-care CD19-targeted CAR T cell therapies are safe and effective for relapsed/refractory ALL and NHL. The findings highlight the feasibility of delivering advanced cellular therapies in resource-limited settings, offering a promising strategy to expand access to life-saving treatments for B cell malignancies.

## Introduction

Cure rates for acute lymphoblastic leukemia (ALL) and non-Hodgkin lymphoma (NHL) have improved substantially.^1^^,^^2^ However, the prognosis for patients with relapsed/refractory (R/R) disease remains poor. Before the introduction of chimeric antigen receptor (CAR) T cell therapy into clinical practice, the Acute Leukemia Working Party of the European Society for Blood and Marrow Transplantation (EBMT) reported a median time to relapse of less than 6.9 months following hematopoietic stem cell transplantation, with a median survival after relapse of only 5.5 months in ALL.^3^ Although initial responses to first-line chemotherapy in B cell NHL are generally favorable, historical data from the pre-CAR T cell era demonstrate high rates of treatment failure: approximately 15%–20% of patients with diffuse large B cell lymphoma (DLBCL) exhibited refractory disease, and an additional 20%–30% experienced relapse within 2 years of initial therapy.^4^ For refractory DLBCL, response rates were only 26%, with a median overall survival (OS) of 6.3 months.^5^

CAR T cell therapy has revolutionized the treatment of patients with R/R ALL and NHL. So far, seven CAR T cell products have been approved by the U.S. Food and Drug Administration (FDA), including five CD19-targeted therapies for use in R/R B cell ALL, DLBCL, follicular lymphoma, and/or mantle cell lymphoma.^6^^,^^7^^,^^8^^,^^9^ Tisa-cel has demonstrated complete remission (CR) rates of 81%–87% in pediatric and young adult patients with R/R B cell ALL across clinical trials and real-world studies. In adult patients with R/R B cell ALL, Brexu-cel has achieved CR rates ranging from 71% to 81%. For DLBCL, three CAR T cell therapies are currently approved, with CR rates of 37%–54% when used as third-line therapy and 65%–66% when administered as second-line therapy.^7^^,^^8^

However, all approved CAR T cell products are centrally manufactured, leading to high production costs and long turnaround times due to the need for shipping cells to and from specialized facilities.^10^ This poses significant barriers to access, particularly for patients in countries outside of Europe and North America, such as Vietnam. To address these challenges, some centers have successfully implemented point-of-care (PoC) CAR T cell manufacturing to reduce costs and delays.^11^^,^^12^ This study is the first to evaluate the safety and preliminary efficacy of CAR T cell therapy using PoC-manufactured CAR T cells (VINCAR-T) in Vietnamese patients with R/R NHL and ALL.

## Results

### Baseline demographics and patient characteristics

Eighteen patients with R/R B cell NHL and ALL met the inclusion and exclusion criteria. Two patients were excluded from the final analyses because their CAR T cell product did not meet release criteria, and the remaining 16 patients comprised the evaluable cohort (Figure S1A). Baseline demographic and clinical characteristics are summarized in Table 1. The median age was 14 years (range, 5–50 years) for ALL and 41 years (range, 32–55 years) for NHL. The median number of prior lines of therapy was 3 (range, 1–3) for ALL and 3 (range, 2–5) for NHL. At baseline, all eight patients with ALL had relapsed disease, of which four were refractory and four had incomplete responses to their last regimen. Bone marrow (BM) blast counts exceeding 5% were observed in two patients. In the NHL cohort, three patients had refractory disease. The remaining five patients with NHL experienced disease relapse, with one classified as refractory and four exhibiting incomplete responses to the most recent line of treatment. One patient with NHL presented with bulky disease, with a tumor size of 117 × 81 mm.

**Table 1:** Baseline demographics and disease characteristics of patients treated with anti-CD19 CAR T cell therapy

Clinical parameters	ALL (*n* = 8)	NHL (*n* = 8)
Age, median (range), years	14 (5–50)	41 (32–55)
Gender, n (%)		
Female	5 (62.5%)	4 (50.0%)
Male	3 (37.5%)	4 (50.0%)
Hemoglobin, median (range), gm%	11.0 (8.2–12.9)	12.9 (8.6–13.8)
White blood cells, median (range), G/l	5.2 (2.9–6)	4.3 (2.4–6.5)
Lymphocytes, median (range), G/l	1.0 (0.5–2.1)	1.1 (0.79–2.6)
Platelets, median (range), G/l	275 (152–315)	170 (107–227)
Liver Function Test		
Total bilirubin, median (range), μmol/L	8.1 (6.4–19.8)	9.2 (5.8–16.5)
SGOT, median (range), U/L	29.2 (19–55)	25.7 (16.1–52.2)
SGPT, median (range), U/L (range)	27.8 (8.4–50.8)	18.9 (8.4–59.8)
Serum creatinine, median (range), μmol/L	41.0 (27.0–58.0)	75.0 (43.0–115.0)
Serum LDH, median (range), U/L	221.5 (124–592)	206 (14.2–1455)
Patients with Additional Comorbidities		
Cardiovascular disease	0	1
Hepatitis B	1	2
Primary indication, n (%)		
Refractory relapse	4 (50.0%)	1 (12.5%)
Non-refractory relapse	4 (50.0%)	4 (50.0%)
Refractory	0 (0%)	3 (37.5%)
Line	3 (1–3)	3 (2–5)
BM blast (>5%)	2/8	NA
BM blast (>2%)	3/8	NA
Bulky lymphoma (i.e., >7.5 cm)	NA	1/8
CNS or other extramedullary involvement before lymphodepletion	Nil	Nil
Length of hospitalization (days)	43 (31–74)	32 (30–34)

Abbreviations are as follows: ALL, acute lymphoblastic leukemia; LDH, lactate dehydrogenase; NHL, non-Hodgkin lymphoma; SGOT, serum glutamic-oxaloacetic transaminase; SGPT, serum glutamate pyruvate transaminase.

VinCART cells were manufactured on-site using the CliniMACS Prodigy system, with a vein-to-vein time of 13 days for the first two patients and 9 days for the remaining patients. Characteristics of the VinCART cells are presented in Table 2. Patients received lymphodepleting chemotherapy with cyclophosphamide and fludarabine, followed by intravenous infusion of CAR T cells at a median dose of 1.90 × 10^6^ cells/kg body weight (range, 0.83–2.17 × 10^6^ cells/kg body weight) (Figure S1B).

**Table 2:** Characterization and quality control parameters of CD19 CAR T cell products

ID	Total cell culture day	%CD3+ (%)	%CAR+ (%)	CAR-T (million cells/kg)	Viability (%)	Mycoplasma	Endotoxin (EU/kg)	Bacteria/fungi	VCN (copies per cell)	Potency (%) E:T = 5:1
BN01	12	99.17	52.82	2.09	90.22	Neg.	<0.05	Neg.	2.46	99.99
BN03	12	99.49	67.92	1.84	95.45	Neg.	<0.05	Neg.	2.25	99.8
BN06	8	99.21	37.95	1.75	99	Neg.	<0.05	Neg.	1.82	99.78
BN07	8	99.22	34.5	1.33	91.44	Neg.	0.086	Neg.	1.69	99.97
BN09	8	99.33	41.96	2.09	98.33	Neg.	<0.05	Neg.	2.01	99.99
BN10	8	99.24	49.64	1.61	98.47	Neg.	<0.05	Neg.	2.14	99.95
BN12	8	99.23	43.34	1.97	97.91	Neg.	<0.05	Neg.	1.82	99.97
BN17	8	99.21	33.76	2.05	98.64	Neg.	<0.1	Neg.	1.96	99.91
BN05	8	99.39	43.25	1.85	97.46	Neg.	<0.05	Neg.	1.8	99.9
BN08	8	98.7	20.2	0.83	92.17	Neg.	<0.05	Neg.	1.87	99.96
BN11	8	99.49	40.12	1.81	98.41	Neg.	<0.05	Neg.	1.82	99.96
BN13	8	99.4	25.28	2.09	98.14	Neg.	<0.05	Neg.	1.73	99.93
BN14	8	99.17	41.13	1.6	95.25	Neg.	<0.05	Neg.	1.78	99.94
BN15	8	99.71	17.24	2.17	96.29	Neg.	<0.05	Neg.	3.44	99.83
BN16	8	99.36	42.98	2	98.18	Neg.	<0.05	Neg.	2.1	99.94
BN18	8	99.16	32.8	1.94	98.46	Neg.	<0.053	Neg.	2.04	99.66
Median (range)	**8 (8,12)**	**99.24 (98.70–99.71)**	**40.63 (17.24–67.92)**	**1.90 (0.83–2.17)**	**98.03 (90.22–99.00)**	**NA**	**NA**	**NA**	**1.92 (1.69–3.44)**	**99.94 (99.66–99.99)**

Abbreviations are as follows: E, effector; NA, not applicable; neg., negative; T, target; VCN, vector copy number.

### Safety

There were no deaths attributed to CAR T cell therapy. Patients with ALL had a total of 162 adverse events (AEs) and no serious AEs (SAEs). One patient with NHL experienced an SAE, and the NHL cohort had a total of 139 AEs (Table 3).

**Table 3:** AEs and SAEs

AE/SAE	ALL	NHL	Notes
SAE	0	1	
AE	162	139	
Unrelated to the CT intervention	7 (4.3%)	11 (7.9%)	flank pain, lymph node pain, rash, abdominal pain, anal pain, hypercalcemia, hypercholesterolemia
Unlikely related to the CT intervention	41 (25.3%)	30 (21.6%)	bone pain, hypocalcemia, decreased albumin, hypokalemia, hypocalcemia, hyponatremia, vomiting, increased uric acid, hypertriglyceridemia, diarrhea
Possibly related to the CT intervention	48 (29.6%)	43 (30.9%)	headache, decreased fibrinogen, fever, elevated bilirubin, increased INR, elevated creatinine, anemia, elevated LDH, increased liver enzymes.
Probably related to the CT intervention	58 (35.8%)	48 (34.5%)	leukopenia, neutropenia, thrombocytopenia
Definitely related to the CT intervention	8 (4.9%)	7 (5.0%)	CRS, ICANS.
**CRS/ICANS**	**Grade**	**ALL**	**NHL**
CRS	Grade 1 and 2	6 (75.0%)	6 (75.0%)
	Grade ≥3	0 (0%)	1 (12.5%)
ICANS	Grade 1 and 2	1 (12.5%)	1 (12.5%)
	Grade ≥3	0 (0%)	0 (0%)
Medications	Tocilizumab	6 (75.0%)	3 (37.5%)
	Dexamethasone	2 (25.0%)	1 (12.5%)

AEs and SAEs occurred following VinCART therapy.

Abbreviations are as follows: AE, adverse event; ALL, acute lymphoblastic leukemia; CRS, cytokine release syndrome; CT, cell therapy; ICANS, immune effector cell-associated neurotoxicity syndrome; NHL, non-Hodgkin lymphoma; SAE, serious adverse event.

Cytokine release syndrome (CRS) occurred in 13 of 16 patients (81.2%), and immune effector cell-associated neurotoxicity syndrome (ICANS) was observed in two of 16 patients (12.5%) (Table 3). Among patients with ALL, CRS was restricted to grade 1–2 events, occurring in 6 of 8 patients (75.0%), with no grade ≥3 CRS reported. ICANS in the ALL cohort was infrequent and mild, with one patient (12.5%) experiencing grade 1 ICANS. In the NHL cohort, CRS was reported in 7 of 8 patients (87.5%), including six patients (75.0%) with grade 1–2 CRS and one patient (12.5%) with grade 3 CRS. The patient who developed grade 3 CRS also experienced grade 1 ICANS (12.5%) and required admission to the intensive care unit. ICANS was not observed in the other patients.

For CRS management, tocilizumab was administered to 6 of 8 patients with ALL (75.0%) and 3 of 8 patients with NHL (37.5%), with a median time to first administration of 1.5 days post-infusion (range, 1–8 days) (Table 3). Patients receiving tocilizumab required a median of 3 doses (range, 1–17). Dexamethasone was administered to two patients with ALL (25.0%) and one patient with NHL (12.5%), with dosing frequencies of 1, 4, and 11 doses, respectively (Table 3).

### Clinical responses

**Figure 1:**
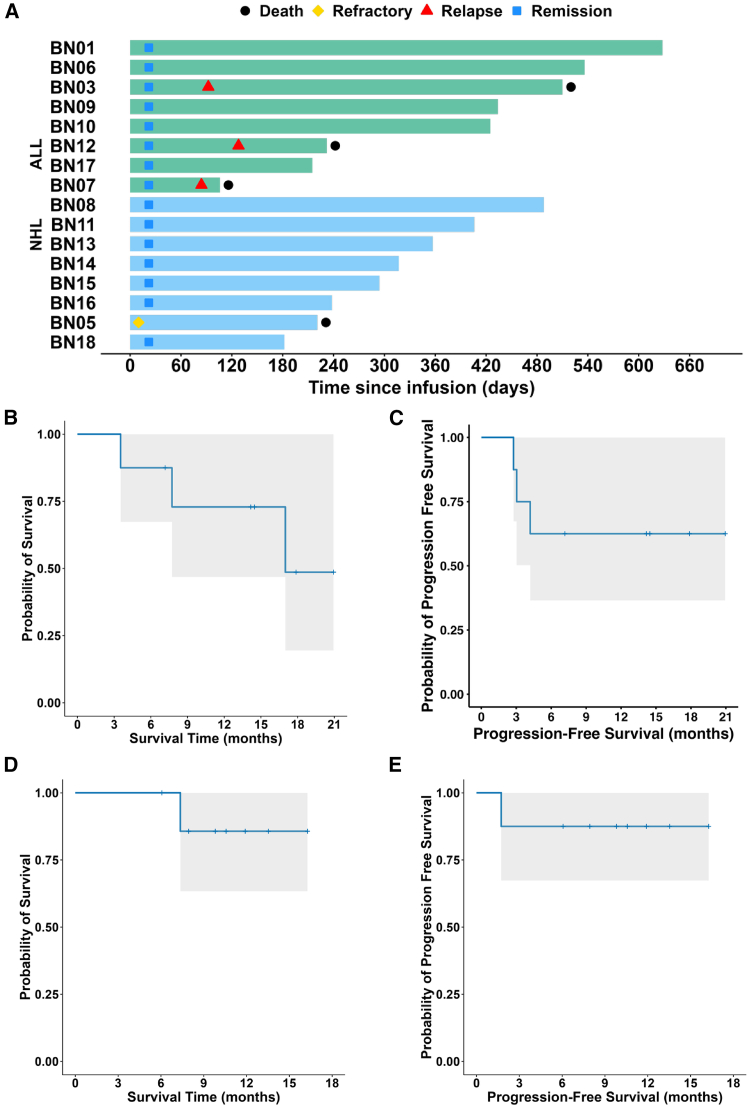
Overall and progression-free survival of ALL and NHL (A) Clinical outcomes of individual patients. Each horizontal bar represents a single patient, grouped by diagnosis: B cell ALL (top, green) and NHL (bottom, blue). (B and C) OS and PFS of the ALL cohort (*n* = 8), respectively. The gray area denotes the 95% confidence interval of the Kaplan-Meier survival estimate. (D and E) OS and PFS of patients with NHL (*n* = 8), respectively. The gray area denotes the 95% confidence interval of the Kaplan-Meier survival estimate. ALL, acute lymphoblastic leukemia; NHL, non-Hodgkin lymphoma; PFS, progression-free survival; OS, overall survival.

In the cohort of ALL patients, the CR rates were 100% at day 30 post-treatment, 75.0% at day 90, and 62.5% at day 180, and these rates remained stable through the most recent follow-up assessments (Table S1). During a mean follow-up of 12.9 months (95% confidence interval [CI]: 7.8–17.9 months) for the ALL group, three patients in the ALL cohort died of disease progression (Figure 1A). The estimated 1-year progression-free survival (PFS) rate for the ALL group was 62.5% (95% CI: 36.5%–100%), and the median PFS was not reached.

Patients with NHL demonstrated a CR rate of 87.5% at both day 90 and day 180, with this rate persisting through subsequent follow-up evaluations (Table S1). The mean follow-up duration was 10.4 months (95% CI: 8.07–12.79 months). One patient experienced disease progression, resulting in death at 7.4 months post-treatment (Figure 1A). The 1-year PFS rate in this group was 87.5% (95% CI: 67.3%–100%), while the median PFS was not reached. Kaplan-Meier plots illustrating OS and PFS for both ALL and NHL cohorts are presented in Figures 1B–1E.

### Correlates of response: CD19^+^ B cells and CAR T cells in patients

**Figure 2:**
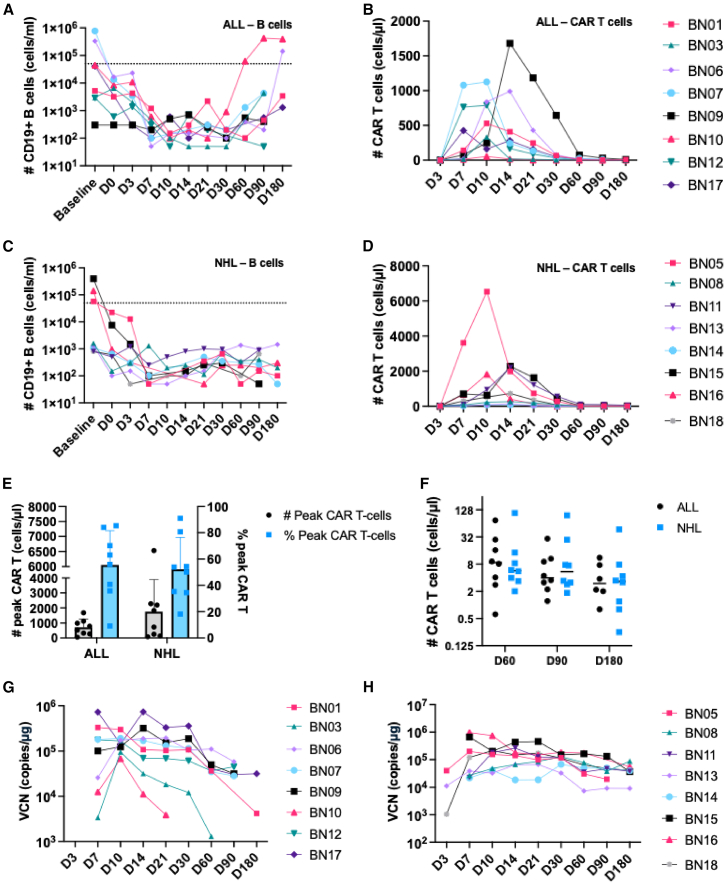
Analysis of B cells and CAR T cells in patients with ALL and NHL (A and B) Flow cytometry analysis of PB samples from patients with ALL from baseline to day 180, quantifying B cells (A) and CAR T cell numbers (B). (C and D) Flow cytometry analysis of B cells (C) and CAR T cell numbers (D) in patients with NHL. The dotted line represents the threshold for B cell aplasia, defined as fewer than 50 B cells/μL. (E) The highest observed numbers and frequencies of CAR T cells in the PB of patients with ALL and NHL. (F) CAR T cell numbers at days 60, 90, and 180 in patients with ALL and NHL. (G and H) Quantitative analysis of CAR VCN in T cells from the PB of patients with ALL (G) and NHL (H). ALL, acute lymphoblastic leukemia; NHL, non-Hodgkin lymphoma; PB, peripheral blood; VCN, vector copy number. The data in E are represented as mean ± SD.

B cells and CAR T cells were monitored to evaluate the therapeutic response. In patients with ALL, CD19^+^ B cell levels declined following conditioning therapy and CAR T cell infusion (Figure 2A). In parallel, CAR T cell concentrations increased progressively, peaking on days 10–14 post-infusion in all patients (Figure 2B). Similarly, patients with NHL exhibited rapid depletion of B cells, corresponding with CAR T cell expansion (Figures 2C and 2D). The median peak CAR T cell count was 658.9 cells/μl (range: 54.0–1680.6) in patients with ALL and 1278.2 cells/μl (range: 82.9–6529.3) in those with NHL, representing a median of 59.55% (range: 9.17–85.30) and 52.07% (range: 18.22–91.06) of the CD3+ T cell subset in flow cytometry analysis, respectively (Figure 2E). CAR T cells persisted at lower levels through the final follow-up on day 180, with a median of 2.99 cells/μl (*n* = 6, range: 0.80–11.12) in patients with ALL and 3.32 cells/μl (*n* = 8, range: 0.25–47.36) in patients with NHL (Figure 2F). Data were not available for two patients who relapsed. Furthermore, quantitative PCR analysis confirmed an initial increase in CAR vector copy number (VCN) in peripheral blood (PB) following infusion, followed by a gradual decline over time in patients with ALL and more sustained persistence in NHL cases (Figures 2G and 2H).

**Figure 3:**
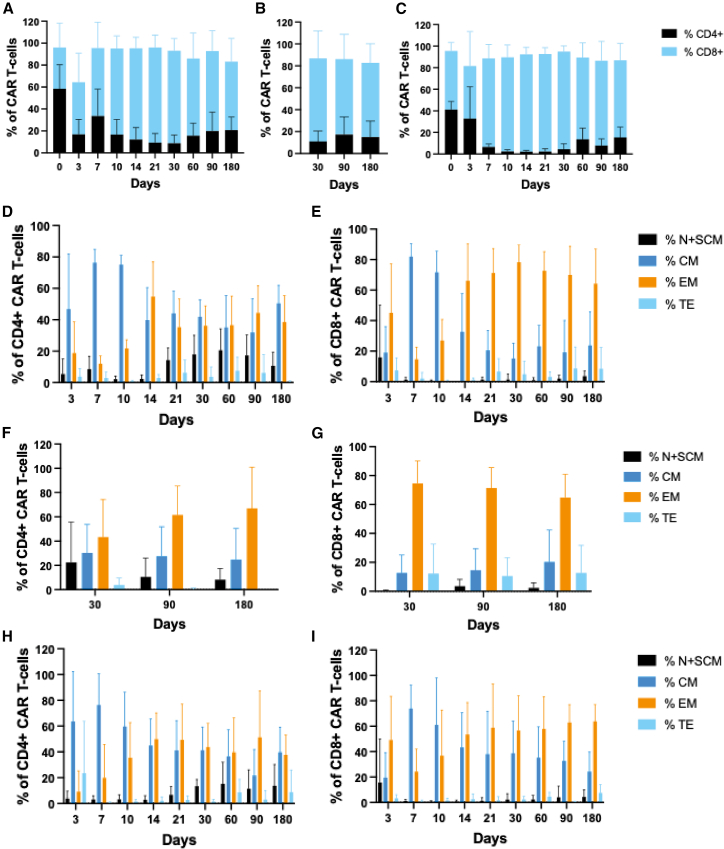
Phenotypic characterization of CAR T cells in patients (A and B) Distribution of CD4+ and CD8+ subsets of CAR T cells in PB (A) and BM (B) of patients with ALL. (C) CD4+ and CD8+ subsets of CAR T cells in PB of patients with NHL. (D and E) Longitudinal immunophenotypic profiling of CD4^+^ (D) and CD8^+^ (E) CAR T cells in the PB of patients with ALL from day 3 to day 180 post-infusion. (F and G) BM analysis of CD4^+^ (F) and CD8^+^ (G) CAR T cells in patients with ALL at days 30, 90, and 180 post-infusion. (H and I) Evaluation of CD4^+^ (H) and CD8^+^ (I) CAR T cells in PB of patients with NHL over the 180-day follow-up period. ALL, acute lymphoblastic leukemia; BM, bone marrow; NHL, non-Hodgkin lymphoma; PB, peripheral blood; VCN, vector copy number. The data are represented as mean ± SD.

The infused CAR T cell products exhibited a balanced CD4:CD8 ratio. However, following infusion, the CAR T cell population became skewed toward predominantly CD8^+^ T cells, as observed in both the PB and BM of patients with ALL (Figures 3A and 3B) and in the PB of patients with NHL (Figure 3C). Both patients with ALL and patients with NHL demonstrated a phenotypic shift from central memory T cells to effector memory T cells around days 10–14 post-infusion (Figures 3D–3I).

### IgG levels

**Figure 4:**
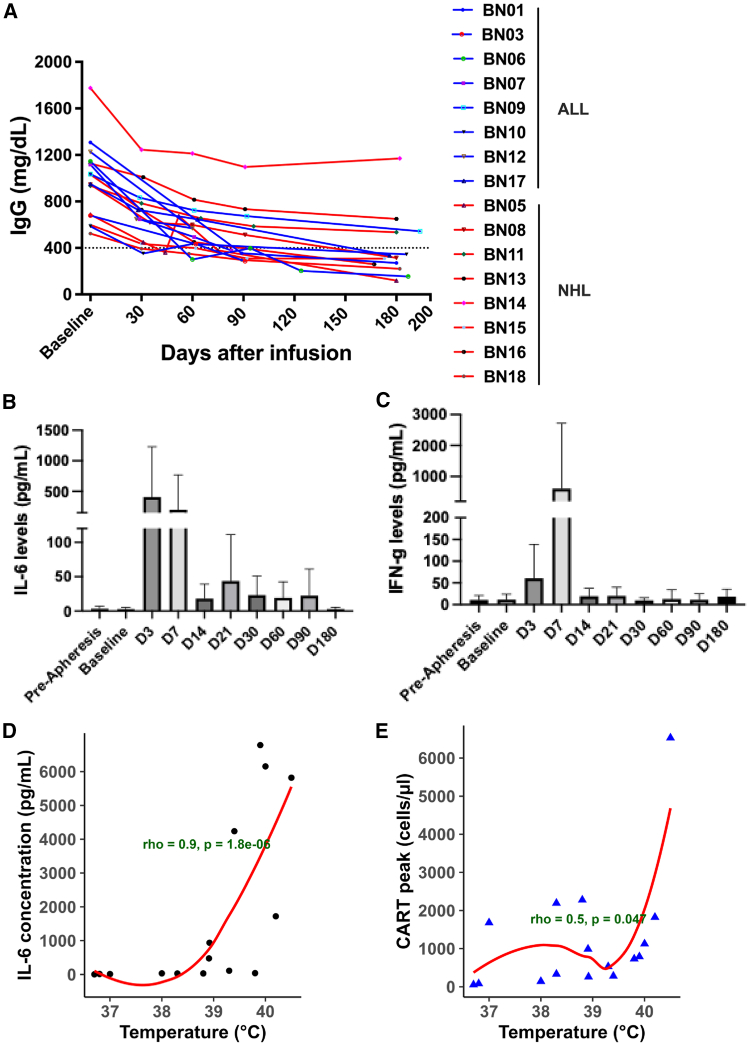
IgG and cytokine levels after CAR T cell infusion (A) Longitudinal assessment of IgG levels in patients after CAR T cell infusion. (B and C) Plasma concentrations of IL-6 (B) and IFN-γ (C) in patients treated with CAR T cells. (D) Correlation between peak IL-6 levels and patients’ maximum recorded temperature. (E) Correlation between patients’ maximum recorded temperature and peak CAR T cell expansion. The data in B and C are represented as mean ± SD.

Serum IgG levels declined in all patients, consistent with B cell depletion. Median IgG concentrations decreased from 990.5 mg/dL at baseline (*n* = 16; range: 524.0–1774.9 mg/dL) to 314.0 mg/dL by day 180 (*n* = 13; range: 120.0–1170.0 mg/dL) (Figure 4A).

Hypogammaglobulinemia, defined as serum IgG < 400 mg/dL, was observed post-treatment in 6 of 8 patients with ALL and 5 of 8 patients with NHL at least at one time point during the 180-day follow-up. On day 180, hypogammaglobulinemia persisted in 4 of 5 patients with ALL and 4 of 7 patients with NHL who remained in CR, as well as in one patient with NHL experiencing disease progression. Unfortunately, IgG data at day 180 were unavailable for three patients with ALL who relapsed. Patients with hypogammaglobulinemia received intravenous immunoglobulin (IVIG) therapy (0.4 mg/kg body weight per dose) to reduce infection risk.

### Cytokine quantification

Serum cytokines were analyzed on days 3, 7, 14, 21, 30, 60, 90, and 180 after CAR T cell infusion. Levels of IL-6 and IFN-γ were elevated on days 3–7 in a subset of individuals following CAR T cell infusion, whereas other cytokines, including GM-CSF, TNF-α, IL-4, and IL-17A, remained unchanged (Figure S2). Among patients who developed CRS, IL-6 levels significantly increased by day 3 post-infusion relative to baseline (*p* = 0.01; Figure 4B). IFN-γ levels were higher on day 7; however, this change did not reach statistical significance (Figure 4C).

A positive association was observed between peak body temperature and both the highest IL- 6 concentration (Spearman ρ = 0.90, *p* = 1.8 × 10^−6^) and peak CAR T cell levels (Spearman ρ = 0.50, *p* = 0.047), indicating that higher febrile responses coincided with increased cytokine release and CAR T cell expansion (Figures 4D and 4E).

Patients were stratified into two groups: those who maintained CR at day 180 and those who experienced refractory disease or relapse (R/R) to explore a potential correlation between these parameters and clinical outcomes. The baseline characteristics and pharmacodynamic parameters of these groups are represented in Table S2. Notably, the R/R group exhibited a higher initial disease burden, as reflected by increased blast frequency in the ALL cohort and larger tumor size in patients with NHL (Figure S3; Table S2). This group also tended to exhibit greater CAR T cell expansion, higher body temperature, and elevated IL-6 levels than the CR group (Figure S3; Table S2). However, no statistically significant differences were found, potentially due to the limited sample size of this cohort.

### Treatment failure and relapse

In the ALL cohort, three patients presented with a disease burden of ≥4% blasts (4%, 5%, and 10%, respectively) in the BM prior to treatment. All three patients achieved CR by day 30 post-infusion. However, two children (BN03 and BN07) with pre-treatment disease burdens of 4% and 10% experienced relapse by day 91 and 83, respectively, and both subsequently succumbed to the disease on days 510 and 106, respectively (Table S1). An additional relapse occurred in an adult patient with ALL (BN12) on day 126 (Table S1). In the NHL cohort, one patient, presenting with a tumor mass measuring 117 × 81 mm, demonstrated refractory disease and died on day 221 (Table S1). The relationship between tumor load, peak CAR T cell and IL-6 levels, and clinical responses is detailed in Table S2.

**Figure 5:**
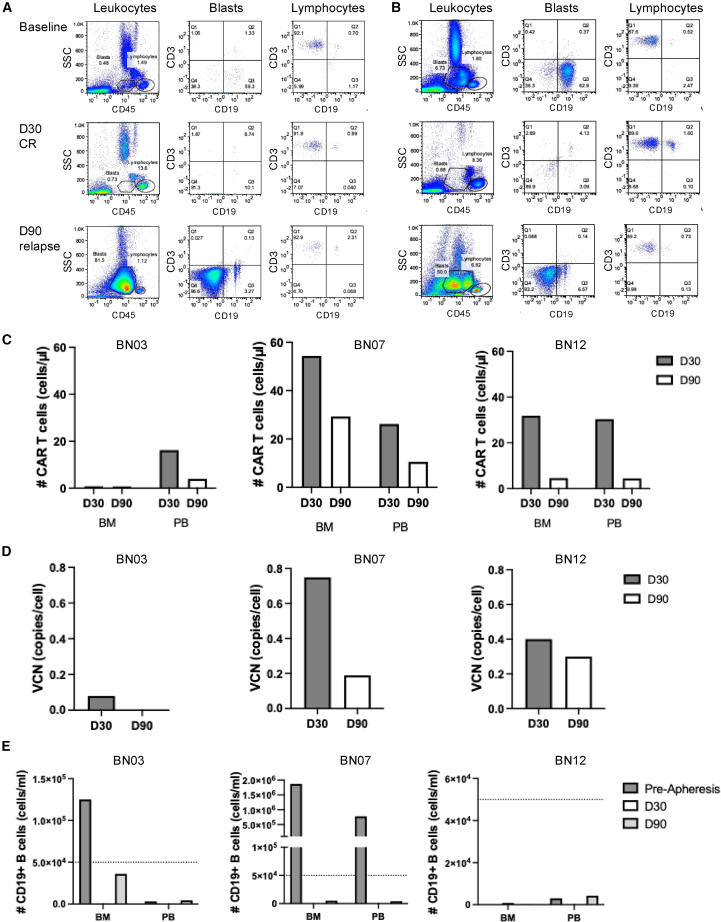
Leukemic blasts and CAR T cells in relapsed patients (A and B) Flow cytometry analysis of CD19 and CD3 expression in CD45^dim^ SSC^low^ leukemic blasts and CD45^bright^ SSC^low^ lymphocytes from patients BN03 (A) and BN07 (B) at three time points: prior to apheresis, day 30 (CR), and day 90 (relapse). (C) Quantification of CAR T cell levels in BM and PB samples from patients BN03, BN07, and BN12 on days 30 and 90, based on flow cytometry. (D) Quantitative PCR analysis of CAR VCN at the corresponding time points in relapsed patients. (E) CD19+ B cell counts in BM and PB samples obtained before apheresis and on days 30 and 90 post-infusion. BM, bone marrow; CR, complete remission; PB, peripheral blood; PCR, polymerase chain reaction; VCN, vector copy number.

All three patients with relapsed ALL exhibited CD19^−^ disease recurrence. In patient BN03, 69.3% CD45^dim^SSC^low^ blasts expressed CD19 prior to apheresis, while 38.9% lacked this marker. Both blast populations disappeared at the time of CR on day 30. At relapse, 95.5% blasts were CD19^−^ (Figure 5A). Similarly, patient BN07 had 62.9% CD19^+^ and 36.3% CD19^−^ blasts prior to apheresis, shifting to 93.2% CD19^−^ blasts at relapse (Figure 5B). Patient BN12 also demonstrated loss of CD19 expression in the blast population at relapse.

Interestingly, the BM and PB of the relapsed patients displayed variable CAR T cell levels, as determined by flow cytometry and quantitative PCR over time (Figures 5C and 5D, respectively). At relapse on day 90, patient BN03 exhibited low levels of CAR T cells (Figures 5C and 5D), which corresponded with an elevated concentration of B cells in the BM (Figure 5E). In contrast, CAR T cells persisted in patients BN07 and BN12 at day 90 (Figures 5C and 5D), corresponding with profound B cell aplasia (Figure 5E), indicating sustained activity and consistent with CD19-negative relapses.

### B cell recovery

In the ALL cohort, B cell recovery (>50,000 B cells/mL) was observed in two patients who remained in CR. These patients exhibited 0% blast frequency in the BM at pre-apheresis (Table S1). Patient BN10 showed re-emergence of peripheral B cells by day 60 following CAR T cell infusion (Figures 2A and S4A), while patient BN06 recovered by day 180 (Figures 2A and S4B). Notably, these patients exhibited low to no detectable CAR T cells as B cells recovered, as measured by flow cytometry (Figures S4C and S4D) and quantitative PCR (Figures S4E and S4F). B cell differentiation was analyzed in BN10 (Figure S5A). By day 7 post-infusion, the patient exhibited profound B cell aplasia. However, by day 90, PB analysis showed evidence of B cell reconstitution, with populations including CD19^+^CD20^+^CD38^+^ pre-B cells, CD19^+^CD20^+^IgD^+^ activated B cells, CD19^+^CD20^+^CD27^+^ memory B cells, and CD38^+^CD138^+^ plasma cells. A concurrent BM sample collected on day 90 revealed a similar pattern of B cell differentiation, though with a predominance of immature CD19^+^CD20^−^CD38^+^ pro-B cells (Figure S5B). The remaining six patients exhibited sustained B cell aplasia, with no evidence of B cell recovery by day 180 (Figure 2A). In the NHL cohort (*n* = 8), all patients demonstrated persistent B cell aplasia throughout the observation period, with no recovery detected up to day 180 post-infusion (Figure 2C).

### Cost for VinCART therapy

The estimated total cost per patient receiving VinCART therapy in our study was approximately US$120,000, which is significantly lower than that of commercial products in the United States, Canada, the European Union, Singapore, and China (Figure S6; Table S3). This included US$80,000 attributed to the manufacturing of the CAR T cell product, which involved reagent and quality control costs of US$39,100, lentiviral vector cost of US$33,000, personnel expenses of US$2,300, and facility costs of US$5,600. This is comparable to other PoC CAR T products manufactured in Spain, Germany, and India, the latter with estimated costs that excludes the viral vector (Table S3). The remaining US$40,000 covered clinical care-related costs, including hospitalization, laboratory testing, and supportive medications administered during treatment and post-infusion monitoring. Two-thirds of the clinical care-related costs were allocated to inpatient expenses, while the remaining one-third covered outpatient and follow-up expenses.

## Discussion

CAR T cell therapy has led to impressive clinical outcomes in hematological cancers; however, technological and manufacturing challenges significantly limit its applications, especially in low- and middle-income countries (LMICs) and resource-limited settings. Decentralized CAR T cell manufacturing can pave the way to accelerate the implementation of CAR T cell therapy, especially in developing countries.^13^ Our results demonstrate that VinCART can be successfully manufactured on-site, ensuring high product quality and a rapid turnaround time.

VinCART products underwent rigorous quality control testing in accordance with regulatory guidance from the U.S. FDA and the European Pharmacopoeia.^14^^,^^15^^,^^16^^,^^17^ Quality assessments included evaluations of safety (sterility, mycoplasma testing, and VCN); purity (T cell and CAR T cell content and contaminating cell populations); and potency (CAR expression and *in vitro* cytotoxic activity). Despite the absence of internationally harmonized release criteria,^18^ our PoC manufacturing process adhered to the standards proposed by Delgado et al. in the EBMT/ European Haematology Association (EHA) CAR T cell Handbook.^19^ We demonstrated high purity, cell viability, cytotoxicity, and reasonable VCN in our CAR T cell products.

### Safety

In our cohort of 16 patients, CAR T cell therapy demonstrated a favorable safety and efficacy profile in the treatment of both ALL and NHL. No treatment-related deaths were observed. Notably, only one case of grade 3 CRS occurred in the NHL group. ICANS was infrequent and mild across both disease cohorts, with only one grade 1 case reported in each. These safety outcomes align favorably with findings from previously published clinical studies (Table S4).^11^^,^^20^^,^^21^

CRS is a frequently observed complication associated with CAR T therapy. Despite its prevalence, optimal strategies for early detection and the appropriate timing for therapeutic intervention remain matters of ongoing debate. Fever is widely regarded as a hallmark clinical feature indicative of early CRS onset; however, distinguishing CRS from infectious etiologies remains challenging in clinical practice.^22^ Tocilizumab is generally recommended for grade 1 CRS if fever persists beyond 72 h following the exclusion of infection or at grade 2 in patients with significant comorbidities or advanced age.^22^^,^^23^ Early administration of tocilizumab is also advised in cases in which two temperature readings ≥38.5°C occur within a 24-h period, spaced at least 4 h apart, or in patients with CRS of grade ≤2, in order to mitigate progression to more severe grades, as reported elsewhere.^24^^,^^25^

In our cohort, CRS was detected by the onset of fever in accordance with international guidelines.^23^ Furthermore, real-time monitoring of IL-6 upon fever occurrence enabled prompt recognition of cytokine elevation, based on a strong correlation observed between peak body temperature and IL-6 concentrations (Spearman ρ = 0.90). This facilitated early administration of tocilizumab, and such rapid intervention might have contributed to the overall mitigation of CRS severity, with only one patient developing grade 3 CRS and no instances of grade 4 or 5 events in our cohort.

### Efficacy

The data revealed high CR rates in both ALL and NHL cohorts, underscoring the robust antitumor efficacy of CAR T cell therapy. In ALL, the CR rate was 100% at day 30 post-infusion. Although three patients experienced relapse during the mean follow-up period of 12.9 months, the overall durability of remission remained favorable, with an estimated 1-year PFS rate of 62.5%, indicating sustained clinical benefit in the majority of patients, comparable to findings reported by Maude et al.^26^ In NHL, CR rates of 87.5% were observed at both day 90 and day 180. The 1-year PFS rate of 87.5% further supports the potential of CAR T cell therapy to induce durable responses in this patient population, surpassing outcomes reported in prior studies (Table S5).^6^^,^^27^^,^^28^ CAR T cells persisted in the majority of patients, indicating sustained therapeutic activity. Although continued follow-up will be essential to assess long-term disease control and to identify factors predictive of durable response, the efficacy outcomes observed in this cohort are encouraging and reinforce the therapeutic promise of PoC CAR T cell therapy across hematologic malignancies.

Pre-treatment disease burden is widely recognized as a critical factor influencing the efficacy of CAR T cell therapy, with higher tumor load associated with lower response rates and poorer outcomes in both patients with ALL and patients with NHL.^6^^,^^26^ Among the eight patients with NHL, one individual with bulky disease died due to disease progression, while CR was observed in the other seven patients without bulky disease. In the ALL group, two of the three patients who relapsed and subsequently died had pre-treatment blast percentages of 4% and 10%, respectively. In retrospect, both patients had a significant CD19-negative blast population at the time of infusion. In subsequent studies, we will use this as an exclusion criterion or shift similar patients to therapy with a dual antigen-targeting CAR T product (e.g., a CD19/CD22 CAR T cell product). The third relapsed patient was 40 years old and exhibited 0% blasts at baseline. Despite robust CAR T cell expansion and persistence post-infusion, leading to sustained B cell aplasia through day 90, the patient relapsed on day 128 with a CD19-negative clone. Notably, adult ALL is associated with a higher risk of relapse and poorer prognosis, which may have contributed to this outcome.^29^

### CAR T cell expansion, persistence, and loss of B cell aplasia in patients

A higher peak of CAR T cell levels has been correlated with initial CR and negative minimal residual disease.^30^ CAR T cell persistence is recognized as a key determinant of sustained therapeutic benefit in both patients with ALL and patients with NHL.^31^^,^^32^ In our study, VinCART products exhibited robust expansion, peaking between days 10 and 14 and gradually declining thereafter, but still remaining detectable at the final follow-up on day 180, which is consistent with other reports.^26^^,^^33^ The cells displayed a central memory phenotype during the first 2 weeks, then transitioned to an effector memory phenotype with cytotoxic functionality in both PB and BM. Central memory T cells are associated with durable CAR T cell persistence and antitumor efficacy,^34^ which aligns with the high CR rates observed and suggests a sustained therapeutic response.

Early loss of B cell aplasia (within 6 months) is considered a risk factor for relapse.^9^^,^^35^ Applying the threshold proposed by Sahai et al. (<50 B cells/μL),^36^ we found that two out of five patients with ALL experienced loss of B cell aplasia, which corresponded with low to undetectable CAR T cell levels at days 60 and 180, respectively. In contrast, all seven patients with NHL maintained B cell aplasia throughout the observation period. Notably, both patients with ALL remained in CR, and the recovered B cells displayed differentiating phenotypes, suggesting reconstitution of normal B cell populations.

### Economic considerations of VinCART therapy

The median cost of CAR T cell therapy in the US is US$620,500 and US$608,100 for ALL and DLBCL, respectively.^37^^,^^38^ In comparison, the total cost per patient in our study was approximately US$120,000, including US$80,000 for CAR T cell production (inclusive of the lentiviral vector) and US$40,000 for clinical care-related costs. Our manufacturing cost of the CAR T cell product alone is comparable to the reported costs in India, where it is US$35,107, excluding the cost of the lentiviral vector, and to those reported by other PoC centers in Spain and Germany.^11^^,^^39^ To further reduce the overall cost of CAR T cell therapy, in addition to PoC manufacturing, key strategies include optimizing CAR T cell production methods, lowering the cost of viral vectors, and minimizing the duration of hospital stays. Our excellent safety profile supports outpatient care of patients until the onset of fever, similar to standard practice in high-resource settings.^40^

### Conclusion

PoC manufacturing of CAR T cells is feasible in resource-limited settings such as Vietnam, offering a cost-effective alternative to centralized production. CAR T cell therapies generated through PoC platforms have demonstrated safety and efficacy in the treatment of B cell ALL and NHL.

## Materials and methods

### Patients and study design

This phase I, single-center trial (NCT06027957) was conducted between August 2023 and June 2025 with approval from the National Ethics Committee (No. 64/CN-HDDD). The study followed the Declaration of Helsinki and ICH-GCP (International Council for Harmonisation - Good Clinical Practice) guidelines. CAR T cell therapy and related clinical costs were fully covered by the sponsor. All patients provided written informed consent. Patients with R/R B cell NHL and ALL were enrolled according to the inclusion and exclusion criteria (for details, see supplemental information).

### Manufacturing CAR T cells using the CliniMACS Prodigy system

Mononuclear cells were collected using the Spectra Optia Apheresis system (Terumo BCT). CD19 CAR T cells were produced with the CliniMACS Prodigy system and Lentigen’s CD19 CAR lentiviral vector (Miltenyi Biotec, Bergisch Gladbach, Germany) for 8–12 days. The lentiviral vector LTG1563 encoded an anti-CD19 single-chain variable fragment derived from FMC63, a CD8 linker, a TNFRSF19 transmembrane domain, a 4-1BB co-stimulatory domain, and a CD3-zeta chain intracellular signaling domain.^11^^,^^41^

Quality control included assessments of CD3^+^ T cells, transduction efficiency, viability, sterility, endotoxin, VCN, and cytotoxic potency (for details, see supplemental information).

Release criteria required 1–2 million ±20% CAR T cells/kg body weight, CD3+ T cells >70%, CAR T cells >10%, viability >70%, negative results for bacteria, fungi, and mycoplasma, endotoxin < 5 EU/mL, VCN < 5, and cytotoxicity against NALM6 cells.

### Intervention

Patients underwent leukapheresis to obtain mononuclear cells for CAR T cell production. They received lymphodepleting conditioning with cyclophosphamide (NHL: 500 mg/m^2^/day and ALL: 250 mg/m^2^/day) and fludarabine (NHL: 30 mg/m^2^/day and ALL: fludarabine 25 mg/m^2^/day) from day −5 to day −3, followed by intravenous infusion of CAR T cells on day 0.

### Toxicity monitoring

AEs and SAEs were recorded according to Common Terminology Criteria for Adverse Events (CTCAE) version 5.0,^42^ with attribution graded in accordance with National Cancer Institute guidelines.^43^ CRS and ICANS were evaluated using the American Society for Transplantation and Cellular Therapy (ASTCT) criteria.^44^ IL-6 levels were measured every 24 h post-infusion and every 8 h following the onset of fever until resolution to guide tocilizumab use. Tocilizumab (8 mg/kg body weight/dose) was administered in response to fever accompanied by elevated IL-6 levels observed at two consecutive time points. Dexamethasone (10 mg per dose) was administered if CRS worsened following tocilizumab treatment.

### Response assessment

ALL response was assessed via BM analysis of blast percentage using flow cytometry at baseline and on days 30, 90, and 180, in accordance with the 2022 National Comprehensive Cancer Network (NCCN) criteria.^45^ NHL response was evaluated using positron emission tomography-computed tomography (PET-CT) at baseline and on days 90 and 180, according to the Lugano classification.^46^

### Monitoring B cells, CAR T cells, and cytokines *in vivo*

PB samples were collected on days 3, 7, 10, 14, 21, 30, 60, 90, and 180 and monitored for CD19+ B cells, CAR T cells, plasma cytokines, and VCN according to the manufacturer’s instructions (for details, see supplemental information).

### Statistical analysis

Response outcomes and survival analyses were reported using frequency tables with absolute values, percentages, and 95% CIs. Categorical variables were compared using Fisher’s exact test and continuous variables using nonparametric tests (the Wilcoxon rank-sum test for independent groups and the Wilcoxon signed-rank test for paired data). Exploratory comparisons between responders and non-responder/relapser for baseline and pharmacodynamic metrics used Fisher’s exact test and the Wilcoxon rank-sum test. Survival was analyzed using Kaplan-Meier methods and compared with the log-rank test. Statistical significance was set at *p* < 0.05. Analyses were performed using R vesion 3.5.2 and GraphPad Prism version 9.

## Data and code availability

The data that support the findings of this study are available from the corresponding author upon reasonable request.

## Acknowledgments

We sincerely thank the doctors and nurses at the Department of Cell Therapy and Regenerative Medicine, Vinmec Healthcare System, for their exceptional patient care. We are grateful to colleagues at the HiTech Center and Laboratory Department for their support in biobanking, quality control, and labor management. Special thanks to Dang Van Duc (German Rheumatism Research Center, Germany) for his support with flow cytometric analysis support, and to Jacek Toporski (Karolinska University Hospital, Sweden), Fabio Ciceri and Jacopo Peccatori (Vita-Salute San Raffaele University, Italy), Rupert Handgretinger (University Hospital of Tübingen, Germany), and Dr. Michael Aigner (University Hospital of Erlangen, Germany) for their generous contributions to clinical and cell manufacturing training. We also acknowledge Miltenyi Biotec for technical training and lentiviral vector support. Most importantly, we thank the patients and their families for their trust and participation.

This investigation was supported by the Vingroup Research Grant (project no: ISC.19.26). The funding body played no role in the design of the study, data collection, analysis, or interpretation, or in writing the manuscript.

The trial was conducted with approval from the National Ethical Committee (no. 64/CN-HDDD) and registered on ClinicalTrials.gov (NCT06027957). The study followed the Declaration of Helsinki and ICH-GCP guidelines. CAR T cell therapy and related clinical costs were fully covered by the sponsor. All patients provided written informed consent.

During the preparation of this work, we used Microsoft Copilot to check grammar and improve clarity. After using this tool, we reviewed and edited the content as needed and take full responsibility for the content of the published article.

## Author contributions

L.T.N., V.T.H., Q.K.B., D.D.N., and V.H.T.P. contributed to the study conception and design. V.T.H., L.T.M.D., T.T.K.P., H.-N.D., P.T.P., H.T.M., T.H.P., and T.M.T.N. were responsible for apheresis, CAR T cell manufacturing and QC, and follow-up analyses. L.T.N., D.D.N., Q.K.B., V.B.L., N.L.P., N.Q.N., M.L.H., Q.L.P., D.Q.T.N., L.M., and Q.N.N. provided patient care. D.D.N., T.H.P., H.-P.N., V.T.H., L.T.M.D., T.T.K.P., and H.-N.D. performed data collection and analysis. L.T.N., V.T.H., D.D.N., H.-P.N., T.H.P., and M.L.H. drafted the manuscript. All authors critically reviewed the manuscript and approved the final version.

## Declaration of interests

The authors declare no competing interests.

## Supplemental information

Document S1. Figures S1–S6, Tables S1, S2, S4, and S5, and Supplemental Methods

Table S3. Costs of CAR T cell therapy

Document S2. Article plus supplemental information
