# Supplementary material for: Outcomes of point-of-care manufactured CAR T cell therapy for B cell acute lymphoblastic leukemia and non-Hodgkin lymphoma in Vietnam
Source: Mol Ther Oncol. 2026 Feb 13;34(1):201156. doi: 10.1016/j.omton.2026.201156 (PMC12969028; doi:10.1016/j.omton.2026.201156)
Supplement: Document S1. Figures S1–S6, Tables S1, S2, S4, and S5, and Supplemental Methods [file mmc1.pdf]

OMTON, Volume *34*

Supplemental information

Outcomes of point-of-care manufactured CAR T cell therapy for B cell acute lymphoblastic leukemia and non-Hodgkin lymphoma in Vietnam

Liem Thanh Nguyen, Duy D. Nguyen, Quoc Khanh Bach, Lan T.M. Dao, Trang Thi Kieu Phan, Hoang - Phuong Nguyen, Hong-Nhung Dao, Trang H. Pham, Phuong T. Pham, Hien T. Mai, Viet Huong T. Pham, Thanh Mai T. Nguyen, Van Binh Le, Nam Lam Phung, Ngoc Quang Nguyen, Michelle L. Hermiston, Quynh Lan Phan, Do Quang Trung Nguyen, Lan Mai, Quoc Nhat Nguyen, and Van T. Hoang

Supplemental information

Methods

Eligibility

Inclusion criteria

- Both sexes, aged 1 to 60 years, diagnosed with relapsed or refractory (R/R) B-cell non-Hodgkin lymphoma (B-NHL) or B-cell acute lymphoblastic leukemia (B-ALL).
- B-ALL refractory to ≥2 cycles of chemotherapy or relapsed after chemotherapy or hematopoietic stem cell transplantation (HSCT); or B-NHL refractory to ≥2 lines of chemotherapy or relapsed after chemotherapy or HSCT.
- CD19 positivity confirmed by immunohistochemistry or flow cytometry.
- Absolute neutrophil count (ANC) ≥ 1.0 G/L without filgrastim support, absolute lymphocyte count ≥ 0.1 G/L, platelet count ≥ 75 G/L, and hemoglobin ≥ 8.0 g/dL.
- Renal function: serum creatinine ≤ 1.5 × upper limit of normal or estimated glomerular filtration rate ≥ 60 mL/min/1.73 m^2.
- Hepatic function: AST and ALT ≤ 5 × upper limit of normal; total bilirubin ≤ 2.0 mg/dL.
- Pulmonary status: no chronic pulmonary diseases requiring continuous medication and no respiratory insufficiency (oxygen saturation SpO_2 > 92% on room air).
- Cardiac function: no arrhythmias, intracardiac or vascular thrombosis, or heart failure, with left ventricular ejection fraction (LVEF) ≥ 45%.
- Willingness and ability to provide written informed consent to participate in the study.
- Female patients of childbearing potential agreed to use effective contraception during the study period.

Exclusion criteria

- Central nervous system involvement at the time of screening; history of autoimmune neurological disorders, neuromyelitis optica, or clinically significant CNS diseases.
- Diagnosed primary immunodeficiency.
- History of veno-occlusive disease.
- Acute medical conditions requiring immediate treatment; active, progressive, or chronic graft-versus-host disease; or active infectious disease.
- History of or concurrent malignancy other than B-cell lymphoma or B-ALL.
- Active hemolytic anemia.
- Receiving immunosuppressive therapy, except for ≤ 30 mg prednisolone or equivalent at the time of CAR-T-cell infusion.
- Critically ill patients or those at risk of premature death, including acute liver failure requiring dialysis, heart failure requiring vasopressors, systemic infection unresponsive to antibiotics, or ECOG performance status ≥ 3 at screening.
- Other severe concomitant diseases (e.g., uncontrolled hypertension or NYHA class III–IV heart failure) or unstable angina within 3 months prior to screening.
- Intolerance to excipients used in cellular products.
- Pregnant or breastfeeding women, or women planning pregnancy during the study period.
- Participation in another clinical trial at the time of screening.

Manufacturing CAR-T cells using the CliniMACS Prodigy system

Mononuclear cell collection was performed using the Spectra Optia Apheresis system with a MNC Spectra Optia Kit (Terumo BCT). Clinical-grade CD19-targeted CAR T-cells were manufactured using the automated CliniMACS Prodigy system with the TCT program and TS520 tubing set (Miltenyi Biotec, Germany). CD4+ and CD8+ T cells were isolated using CliniMACS CD4 and CD8 Reagents (Miltenyi Biotec, Germany) and cultured in Miltenyi's

TexMACS GMP Medium supplemented with 3% human AB serum (HiMedia, India) and recombinant human IL-7 and IL-15 (Miltenyi Biotec, Germany). Cells were activated with MACS® GMP T Cell TransAct™ for anti-CD3/CD28 co-stimulation overnight before transduction with Lentigen's CD19 CAR lentiviral vector (Miltenyi Biotec, Germany). Transduced T cells were expanded in TexMACS GMP Medium supplemented with 3% human AB serum (HiMedia, India) and recombinant human IL-7 and IL-15 for 8-9 days. On the day of infusion, CAR T-cells were harvested in 0.9% NaCl (Bbraun, Vietnam) supplemented with 0.5% human serum albumin (Grifols Biologicals Inc., USA).

Quality controls of CAR-T cells

Flow cytometry

CAR T-cells were stained with CD45 VioBlue, CD4 VioGreen, CD3 FITC, CD16/CD56 PE, CD19 PE-Vio 770, CD14 APC, CD8 APC-Vio 770 antibodies, and 7-AAD, and analyzed using a MACS Quant 10 (Miltenyi Biotec, Germany) following the manufacturer's instructions to assess immunophenotype and viability. Transduction efficiency was determined using CD45 VioBlue, CD4 VioGreen, CD3 FITC, CAR-DR PE, CD14 APC, CD8 APC-Vio 770, and 7-AAD (Miltenyi Biotec, Germany). Flow cytometry data were analyzed using MACSQuant Analyzers software.

Sterility

Sterility testing for bacteria and fungi was performed using the BacT/Alert3D microbial detection system (bioMérieux, Durham, North Carolina) at the CAP-accredited Vinmec laboratory department, Vinmec Times City Hospital. Mycoplasma contamination was assessed using the MycoAlert Mycoplasma Detection Kit (Lonza, Switzerland), and endotoxin levels were measured with the EndoSafe-PTS Kit (Charles River Laboratories, USA) according to the manufacturer's instructions.

Vector copy number was quantified using TaqMan real-time PCR with the MACS COPYcheck Kit (Miltenyi Biotec, Germany), which measures the lentiviral gag gene and the human reference gene PTBP2, following the manufacturer's instructions.

Potency assay

CAR T-cells were co-cultured with the CD19+ NALM6 cell line at an effector-to-target cell ratio of 5:1, stained with CD3 FITC, CD19 PE-Vio 770, and 7-AAD, and analyzed using a MACSQuant 10 (Miltenyi Biotec, Germany). The number of CD19+ cells was quantified and compared to samples containing only target cells. Additionally, the supernatant from the co-culture was collected, and concentrations of GM-CSF, Granzyme B, IFN-γ, IL-2, IL-4, IL-6, IL-10, IL-17A, IL-21, MCP-1 (CCL2), Perforin, and TNF-α were measured using the human MACSPlex Cytotoxic T/NK Cell Kit (Miltenyi Biotec, Germany) according to the manufacturer's instructions.

Monitoring B-cells, CAR T-cells, and cytokines in vivo

Peripheral blood samples from treated patients were collected on days 3, 7, 10, 14, 21, 30, 60, 90, and 180. Peripheral blood mononuclear cells (PBMCs) were isolated by density gradient centrifugation using Ficoll-Paque™ PREMIUM density gradient media (Cytiva, Sweden). B-cell quantification was performed by flow cytometry using the following antibodies: CD45 Vioblue, CD4 Viogreen, CD3 FITC, CD16/CD56 PE, CD19 PE-Vio® 770, CD14 APC, CD8 APC-Vio® 770, and 7-AAD, analyzed on a MACSQuant 10 (Miltenyi Biotec, Germany). CAR T-cell numbers and phenotypes were assessed by staining with CD45RA Vioblue, CD223 Vioblue, CD4 Viogreen, CD3 FITC, CD14-PerCP-Vio700, CD15-PerCP-Vio700, CAR-DR PE, CD62L PE-Vio® 770, CD279 PE-Vio® 770, CD45RO APC, CD366 APC, CD8 APC-Vio® 770, and 7-AAD (Miltenyi Biotec, Germany). Flow cytometry data were analyzed using MACSQuant® Analyzer software.

Plasma levels of GM-CSF, IFN-α, IFN-γ, IL-2, IL-4, IL-5, IL-6, IL-9, IL-10, IL-12p70, IL-17A, and TNF-α were measured using the MACSPlex Cytokine 12 Kit (Miltenyi Biotec, Germany) according to the manufacturer's instructions.

CAR T-cell copy number was quantified by TaqMan real-time PCR using the MACS® COPYcheck Kit (Miltenyi Biotec, Germany) following the manufacturer's instructions.

Statistical analysis

Response outcomes and survival analysis were reported using frequency tables with absolute values, percentages, and 95% CIs. Categorical variables were compared using Fisher's exact test, and continuous variables using nonparametric tests (the Wilcoxon rank-sum test for independent groups and the Wilcoxon signed-rank test for paired data). Exploratory responder versus non-responder/relapser comparisons of baseline and pharmacodynamic metrics used Fisher's exact and Wilcoxon rank-sum tests. Survival was analyzed via Kaplan-Meier methods and compared using the Log-rank test. Statistical significance was set at $p < 0.05$. Analyses were performed in R vesion 3.5.2 and GraphPad Prism version 9.

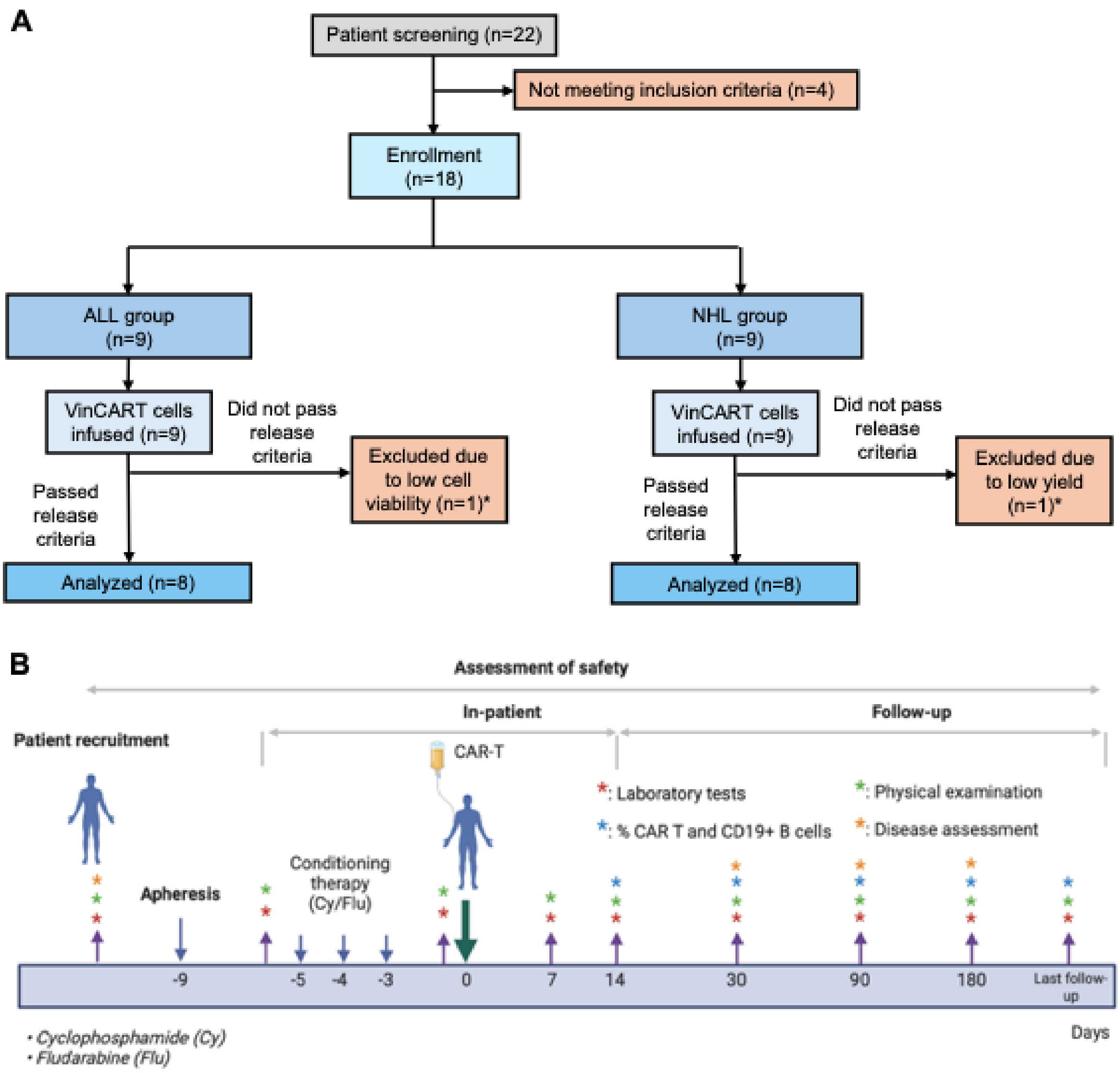

Figure S1. Patients and study design. A. Consort diagram of patients included in the VinCART trial. *Excluded from the evaluable cohort due to not meeting protocol eligibility criteria but still infused with CD19 CAR-T. **B.** Study timeline for CAR T-cell therapy administration and follow-up. Patients received apheresis on day -9 (or day -13 in the first two patients), lymphodepleting conditioning therapy with cyclophosphamide (Cy) and fludarabine (Flu) from days -5 to -3, CAR-T infusion on day 0, and scheduled follow-up visits for safety, laboratory testing, and outcome assessments through day 180 and last visit.

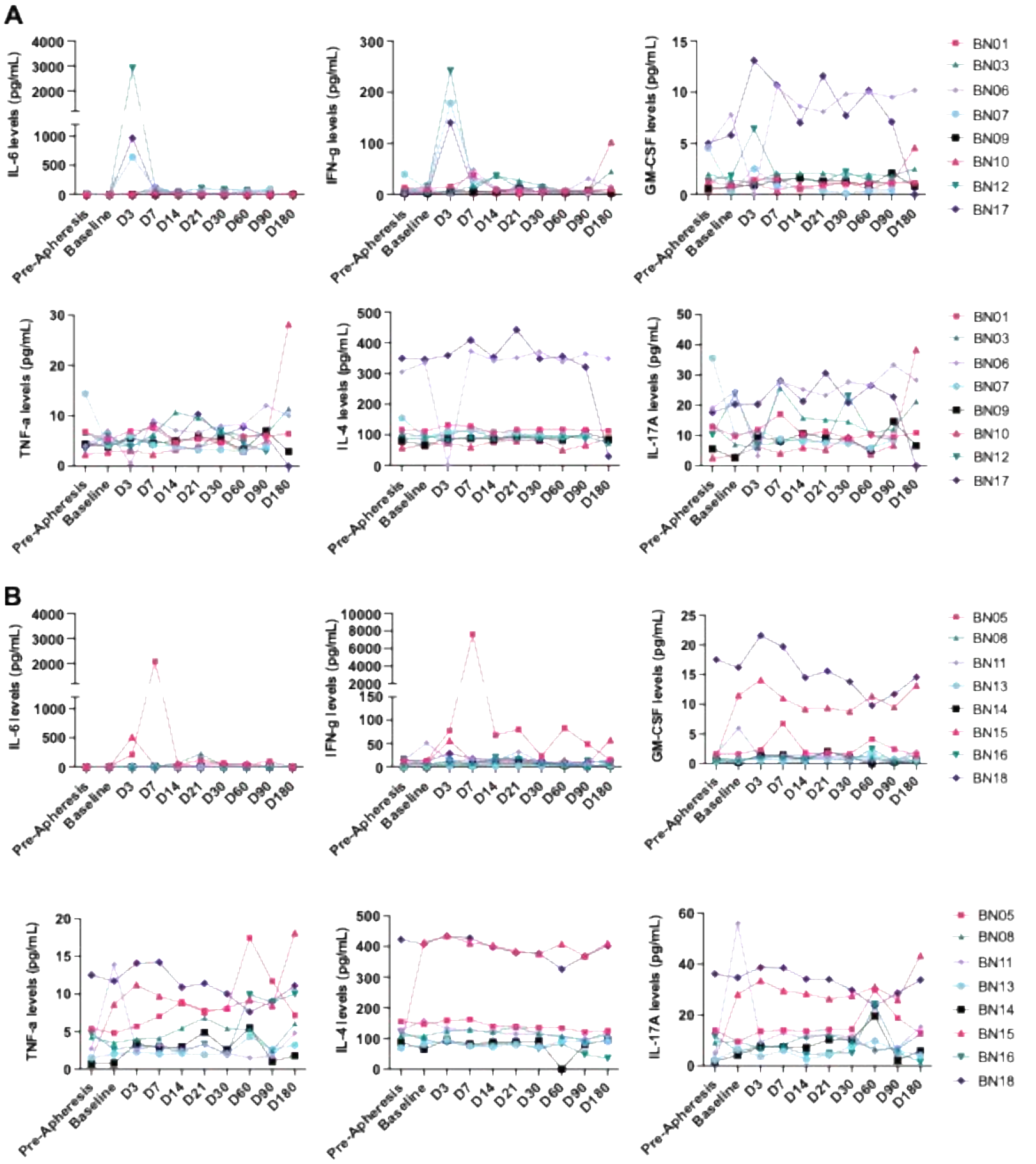

Figure S2: Cytokine levels in plasma of ALL (A) and NHL (B) patients treated with CAR T-cells. IL-2, IL-9, IL-10 levels remained below the assay limit of detection.

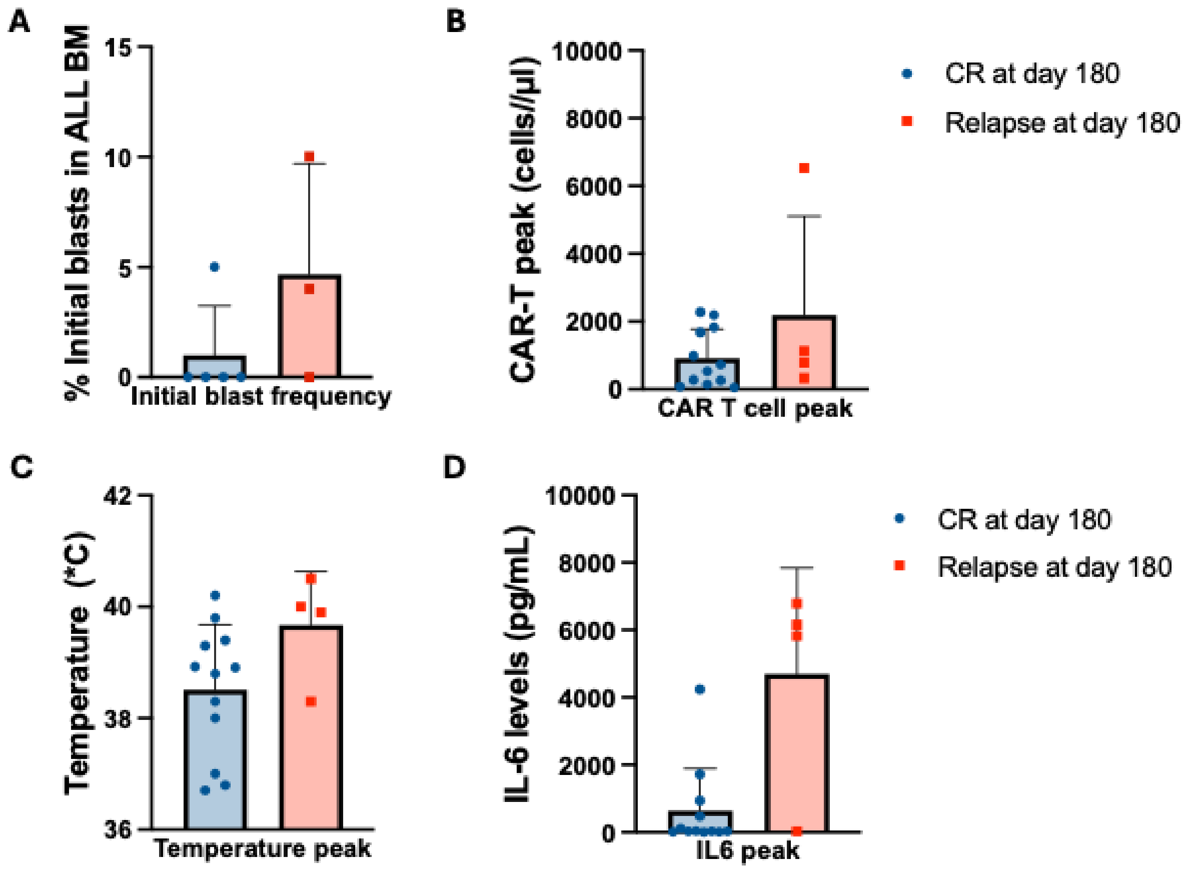

Figure S3. Correlations with clinical outcome. (A) Frequency of leukemic blasts in the BM of ALL patients at baseline, comparing those who achieved CR at day 180 with those who experienced disease refractoriness or relapse (R/R). **(B)** CAR T-cell levels measured by flow cytometry in the PB of the CR and R/R groups. **(C–D)** Peak body temperature (C) and IL-6 concentration (D) recorded following CAR T-cell infusion in both patient groups. Abbreviations: bone marrow (BM), complete remission (CR), peripheral blood (PB), and refractoriness/relapse (R/R).

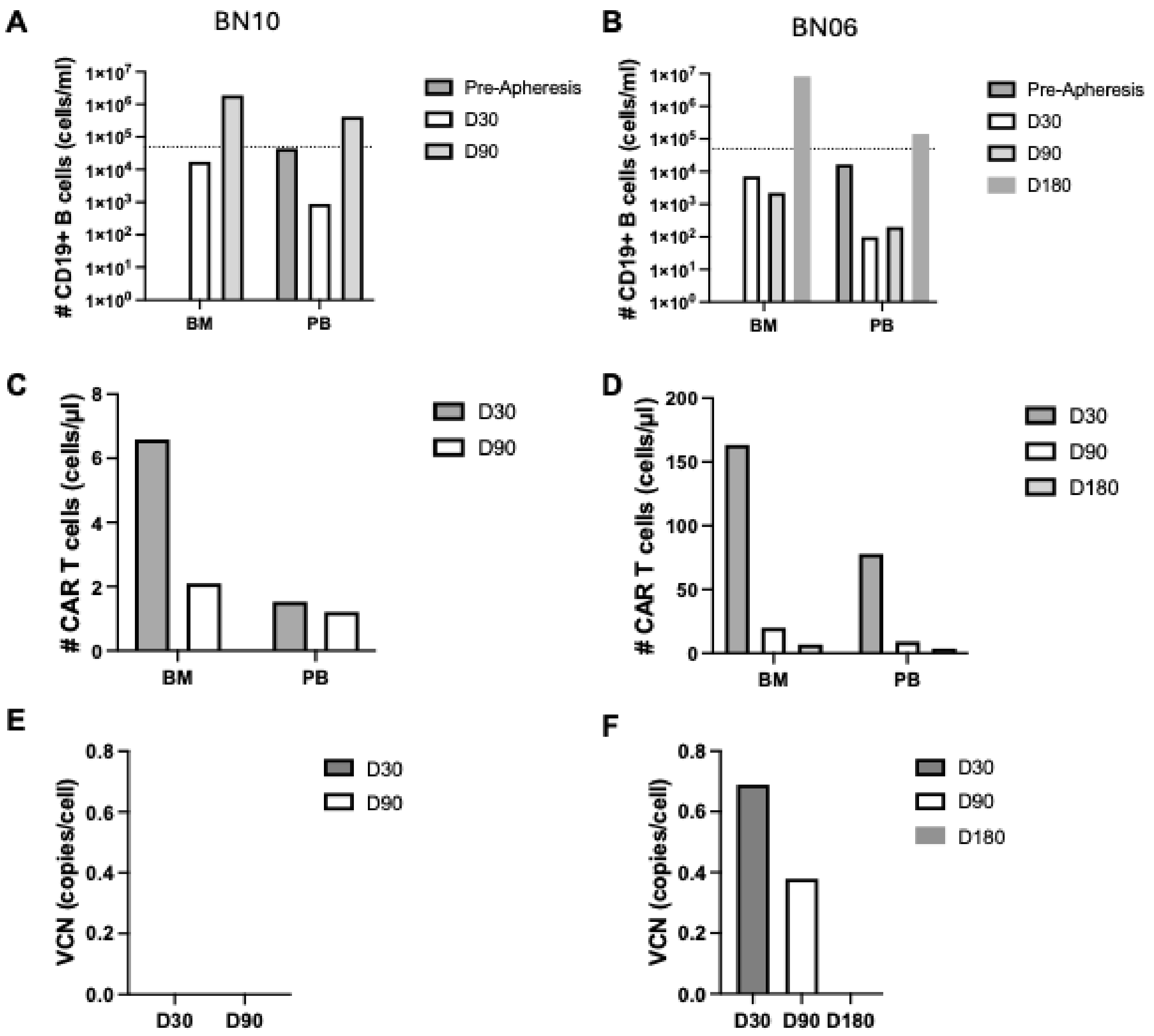

Figure S4: CD19⁺ B cell and CAR T-cell levels in patients with B cell recovery. (A, B) Flow cytometry analysis of the BM and PB from patients BN10 (A) and BN06 (B) at pre-apheresis (only available for PB), day 30, day 90, and day 180. **(C, D)** Quantification of CAR T-cell by flow cytometry in the BM and PB of patients BN10 (C) and BN06 (D) at day 30, day 90, and day 180. **(E, F)** Vector copy number (VCN) in the BM and PB of patients BN10 (E) and BN06 (F), measured by quantitative PCR at day 30, day 90, and day 180. Abbreviations: bone marrow (BM), complete remission (CR), day (D), peripheral blood (PB), polymerase chain reaction (PCR), and vector copy number (VCN).

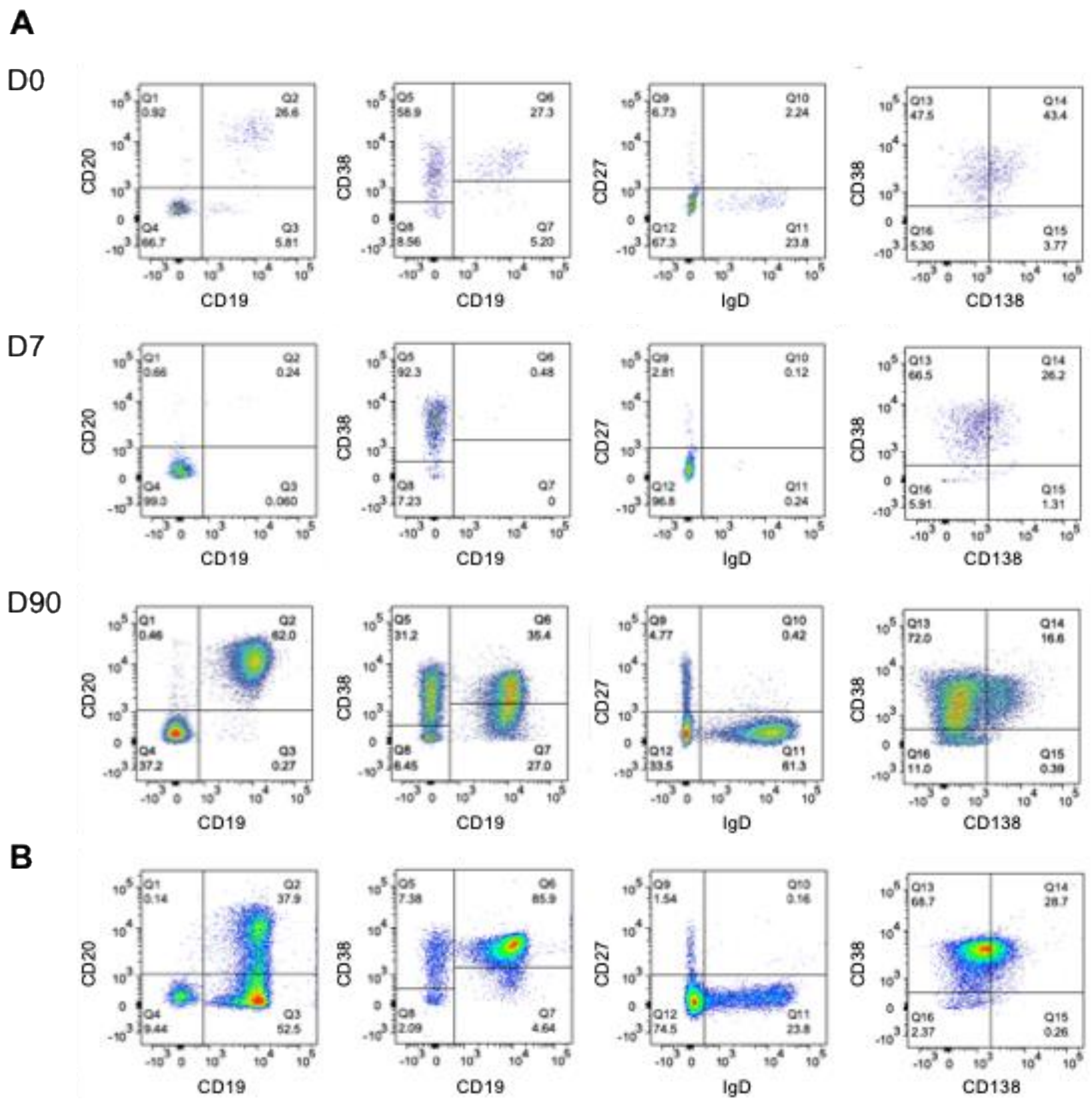

Figure S5: B-cell aplasia and hypogammaglobulinemia recovery in patients following CAR T-cell infusion. (A) B-cell recovery in patient BN10. On day 7 post-infusion, the patient exhibited B-cell aplasia. By day 90, B-cells had recovered in the PB, displaying differentiation into CD19+CD20+CD38+ pre-B cells, CD19+CD20+IgD+ activated B cells, CD19+CD20+CD27+ memory B cells, and CD38+CD138+ plasma cells. **(B)** BM sample from day 90 revealed a similar differentiation pattern, but with a higher frequency of immature CD19+CD20−CD38+ pro-B cells. Abbreviations: bone marrow (BM), day (D), peripheral blood (PB).

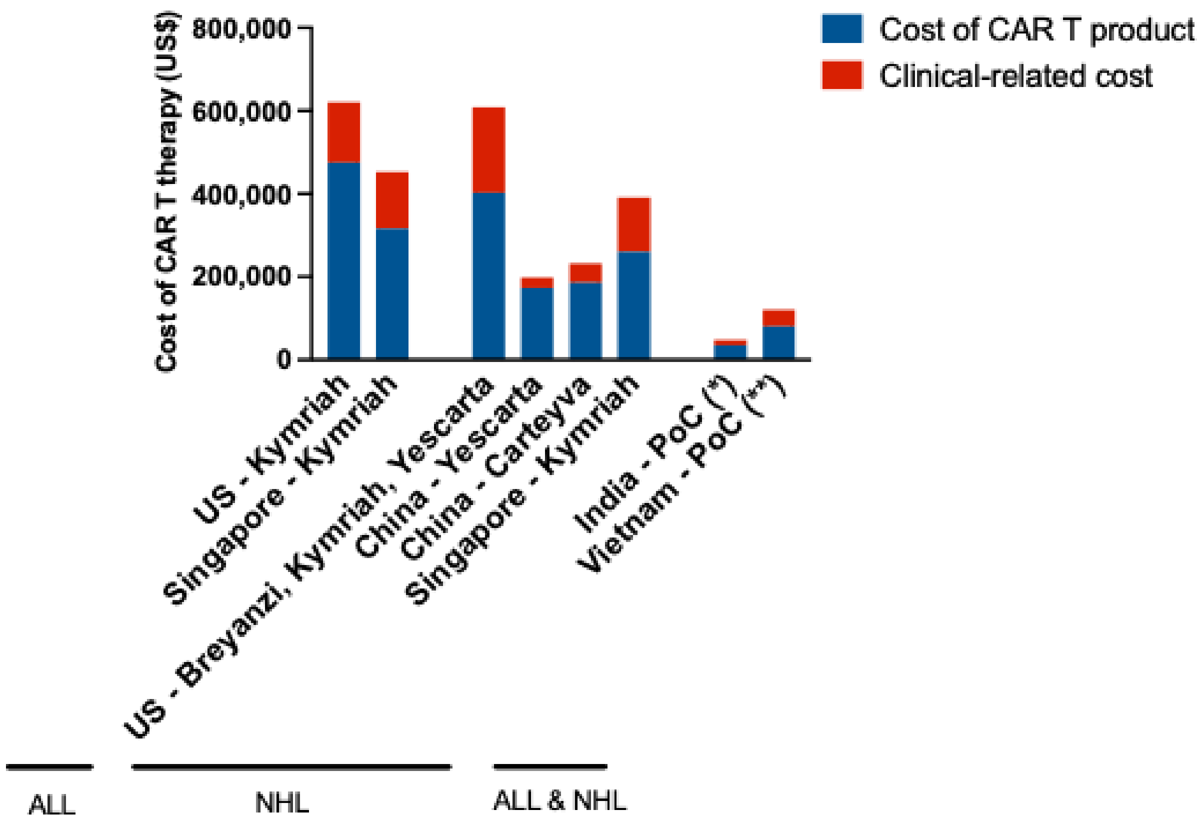

Figure S6: CAR T therapy cost for ALL and NHL. (*) Exclusive cost of vector; (**) Inclusive cost of vector. Abbreviations: PoC, Point of Care.

Table S1. Disease burden and outcomes of individual patients

Patient ID	Age	Tumor size (mm x mm)	BM blast before treatment	CAR T-cell peak (cells/µl)	Follow-up duration (months)	Response at day 30	Response at day 90	Response at day 180
**ALL**								
BN01	8	NA	5%	528.9	20.9	CR	CR	CR
BN03	12	NA	10%	331.9	17.0	CR	Relapse	Relapse
BN06	5	NA	0%	989.4	17.9	CR	CR	CR
BN07	16	NA	4%	1125.9	3.5	CR	Relapse	Death
BN09	24	NA	0%	1680.6	14.5	CR	CR	CR
BN10	11	NA	0%	54.0	14.2	CR	CR	CR
BN12	50	NA	0%	788.9	12.8	CR	CR	Relapse
BN17	27	NA	0%	280.5	7.2	CR	CR	CR
**NHL**								
BN05	35	117 x 81	NA	6529.3	7.4	NA	No response	No response
BN08	55	Nil	NA	260.2	16.3	NA	CR	CR
BN11	32	Nil	NA	2194.3	13.5	NA	CR	CR
BN13	38	Nil	NA	142.5	11.9	NA	CR	CR
BN14	40	8x19	NA	82.9	10.6	NA	CR	CR
BN15	45	12x15	NA	2277.5	9.8	NA	CR	CR

BN16	50	Nil	NA	1825.2	7.9	NA	CR	CR
BN18	42	Nil	NA	731.3	6.1	NA	CR	CR

Abbreviations: ALL, acute lymphoblastic leukemia; BM, bone marrow; CR, complete remission; NA, not applicable; NHL, non-Hogdkin lymphoma.

Table S2. Baseline characteristics and pharmacodynamic metrics stratified by clinical response

**Clinical Parameters**	**CR at day 180 (the CR group)**	**Non-responders/relapsed patients (the R/R group)**	**p value**	**p adj BH**
**Baseline characteristics**				
Age (years)	35.0 [20.8, 42.8]	25.5 [15.0, 38.8]	0.903	1.000
Serum LDH (U/L)	202.5 [158.8, 272.2]	277.0 [209.8, 618.8]	0.203	0.568
Female sex, n (%)	8 (66.7%)	1 (25.0%)	0.262	0.610
High disease burden (ALL BM blast ≥ 5%; NHL: bulky lymphoma)	1 (8.3%)	2 (50.0%)	0.136	0.475
**Pharmacodynamic metrics**				
Total CAR T-cell dose	1.8 [1.7, 2.0]	2.0 [1.7, 2.1]	0.504	0.785
Peak CAR T-cells (cells/μL)	630.1 [230.8, 1716.7]	957.4 [674.6, 2476.8]	0.363	0.726
Peak IL-6 (pg/mL)	35.3 [22.1, 589.8]	5987.5 [4372.0, 6312.5]	0.06	0.421

Other baseline laboratory parameters				
White blood cells (G/L)	5.1 [3.1, 5.7]	4.4 [2.8, 5.9]	1	1.000
Lymphocytes (G/L)	1.1 [1.0, 1.4]	0.8 [0.7, 0.8]	0.029	0.403
Platelets (G/L)	187.0 [150.5, 238.0]	227.0 [171.2, 288.0]	0.504	0.785
Total bilirubin (μmol/L)	8.7 [7.3, 10.2]	11.4 [10.3, 13.6]	0.129	0.475
SGOT (U/L)	27.2 [23.2, 31.3]	29.4 [22.2, 39.5]	0.952	1.000
SGPT (U/L)	22.5 [14.7, 33.5]	25.1 [17.8, 36.4]	0.671	0.939
Creatinine (μmol/L)	50.0 [42.0, 78.8]	50.5 [42.0, 62.2]	0.808	1.000

Notes: Continuous variables are median [IQR] and were compared using the Mann–Whitney U (Wilcoxon rank-sum) test. Categorical variables are n (%) and were compared using Fisher's exact test. Two-sided p values are reported; Benjamini–Hochberg adjusted p values are provided for reference. Patient level disease burden measures are summarized in Table S1.

Abbreviations: BH, Benjamini–Hochberg; CAR T, chimeric antigen receptor T-cells; IL-6, interleukin 6; LDH, lactate dehydrogenase.

Table S3. Costs of CAR T-cell therapy across commercial and point-of-care manufacturing settings.

Due to its size, this table is provided as a separate Excel file.

Table S4. CRS and ICANS of CAR T-cell therapy

	CRS		ICANS		ICU requirement	Early Death
Author	Total number of CRS (%)	CRS ≥ 3 $level$ (%)	Total number of ICANS (%)	ICANS ≥3 (%)		
Dourthe M-E et al. (1)	59	20	24	8	35	1
Kochenderfer JN	NA	26.7	33.3	NA	NA	1
Palani HK et al. (2)	90	10	Nil	Nil	No	No
Locke FL et al. (3)	92	11	67	33	0	
Schuster SJ* et al. (4)	58	22	20	11	24	3
Abramson JS et al. ** (5)	42	2	30	10	4	
**Liem NT et al.**	**81.3**	**6.3**	**12.5**	**0**	**1**	**No**

*The JULIET study employed a grading scale and management guideline developed by researchers at the University of Pennsylvania, while the ZUMA-1.

**TRANSCEND trials adopted a version formulated by a panel of experts convened by the National Cancer Institute and later published by Lee et al (6).

Abbreviations: CRS, cytokine release syndrome; ICANS, immune effector cell-associated neurotoxicity syndrome; ICU, intensive care unit.

Table S5. Outcomes of CAR T-cell therapy

Authors	ORR (%)	CR (%)	Median PFS, mo (95% CI)	Median OS, mo (95% CI)	Median follow-up duration, mo
ALL					
Maude SL et al. (9)	81	81[a]	–	76 (63 – 86)	13.1
**Liem NT et al.**	**100**	**100**	**NE (1-year PFS rate of 62.5%)**	**NE (1-year OS rate of 75%)**	**10.4**
NHL					
Locke FL et al. (3)	83	58	5.9 (3.3–15.0)	NR (12.8-NE)	27.1
Schuster SJ et al. (7)	53	39	2.9 (2.3–5.2)	11.1 (6.6–23.9)	40.3
Abramson JS et al. (8)	73	53	6.8 (3.3-12.7)	27.3 (16.2-45.6)	19.9
**Liem NT et al.**	**87.5**	**87.5**	**NE (1-year PFS rate of 87.5%)**	**NE (1-year OS rate of 87.5%)**	**12.9**

Abbreviations: CRR, complete remission rate; mo, month; NE, not estimable; NR, not reached; ORR: overall response rate; OS, overall survival; PFS, progression-free survival.

[a] 60% of patients achieved CR and 21% of patients achieved CR with incomplete blood count recovery (CRi).

References

1. Dourthe M-E, Rabian F, Yakouben K, Chevillon F, Cabannes-Hamy A, Méchinaud F, Grain A, Chaillou D, Rahal I, Caillat-Zucman S, Lesprit E, Naudin J, Roupret-Serzec J, Parquet N, Brignier A, Guérin-El Khourouj V, Lainey E, Caye-Eude A, Cavé H, Clappier E, Mathis S, Azoulay E, Dalle JH, Dhédin N, Madelaine I, Larghero J, Boissel N, Baruchel A. Determinants of CD19-positive vs CD19-negative relapse after tisagenlecleucel for B-cell acute lymphoblastic leukemia. Leukemia. 2021 2021/12/01;35(12):3383-3393. doi:10.1038/s41375-021-01281-7.

2. Palani HK, Arunachalam AK, Kulkarni U, Yasar M, Venkatraman A, Palanikumar S, Radhakrishnan RN, Solomon M, Rajasekaran A, Bankar A, Datari PVR, Selvarajan S, Korula A, Dash P, Schneider D, Wirthlin L, Abraham A, George B, Mathews V. Safety, efficacy and total cost of point-of-care manufactured anti-CD19 CAR-T cell therapy in India: VELCART trial. Molecular Therapy Oncology. 2025;33(2). doi:10.1016/j.omton.2025.200977.

3. Locke FL, Ghobadi A, Jacobson CA, Miklos DB, Lekakis LJ, Oluwole OO, Lin Y, Braunschweig I, Hill BT, Timmerman JM, Deol A, Reagan PM, Stiff P, Flinn IW, Farooq U, Goy A, McSweeney PA, Munoz J, Siddiqi T, Chavez JC, Herrera AF, Bartlett NL, Wiezorek JS, Navale L, Xue A, Jiang Y, Bot A, Rossi JM, Kim JJ, Go WY, Neelapu SS. Long-term safety and activity of axicabtagene ciloleucel in refractory large B-cell lymphoma (ZUMA-1): a single-arm, multicentre, phase 1-2 trial. Lancet Oncol. 2019 Jan;20(1):31-42. eng. Declaration of interests FLL has served on scientific advisory boards for Kite and Novartis and reports consultancy fees for Cellular Biomedicine and research support from Forma Therapeutics. AGh has received speaker fees and research funding from, and served on scientific advisory boards for, Kite. CAJ reports personal fees from Kite, Novartis, Precision Bioscience, Bayer, Pfizer, and Humanigen. DBM has received grants and research funding from, and served on scientific advisory boards for, Kite. IB reports speaker fees from Kite.

BTH has served on advisory boards for Gilead. AD has served on advisory boards for Kite, Agios, and Novarits, and received research funding from Bristol-Myers Squibb. PMR reports research funding from Seattle Genetics, and has served on advisory boards for, and received personal fees from, Curis. IWF reports research funding from Kite, Agios, ArQule, Beigene, Calithera, Celgene, Constellation, Curis, Forma, Forty-Seven, Genentech, Gilead, Incyte, Infinity, Janssen, Merck, Novartis, Pfizer, Pharmacyclics, Portola, Seattle Genetics, Takeda, TG Therapeutics, Trillium, Verastem, and Roche. UF reports travel support from Kite and honoraria from Celgene. AGo has participated in speakers bureaus for, and received research funding from, Takeda, Kite, Gilead, Pharmacyclics, Janssen, Genentech, and Acerta, and has a leadership role at COTA, a for-profit health-care company. PAM reports speaker and consultant fees from Kite. JM has participated on speakers bureaus or advisory boards for Kite, Pharmacyclics, Janssen, Bayer, Alexion, Pfizer, Juno, Celgene, Bristol-Myers Squibb, Genentech, and Kyowa. TS reports travel support from Kite, speaker and consultancy fees from Pharmacyclics; speaker fees from Janssen and Seattle Genetics, and consultancy for Juno and BeiGene. JCC has participated in advisory boards or speakers bureaus for Kite, Genentech, Novartis, Bayer, and Janssen, and has received research support from Merck. AFH reports grants, personal fees, and consultancy for Bristol-Myers Squibb, Genentech, Merck, Pharamcyclics, and Kite, and grants and consultancy for Gilead. NLB reports research funding from Affimed, Bristol-Meyers Squibb, Celgene, Forty Seven, Genentech, Gilead, Immune Design, Kite, Merck, Millennium, Pharmacyclics, and Acerta, and has served on advisory boards for Pfizer. LN, JMR, AX, YJ, JJK, and WYG, are employed by Kite, and have equity ownership in Gilead. AB and JSW report employment and an issued patent with Kite. SSN reports personal fees and research support from Kite, Merck, and Celgene, research support from Bristol-Myers Sqibb, Poseida, Cellectis, Karus, and Acerta Pharma, and personal fees from Novartis, Pfizer, and Unum Therapeutics. LJL, OOO, YL, JMT, and PS declare no

competing interests. Epub 20181202. doi:10.1016/s1470-2045(18)30864-7. Cited in: Pubmed; PMID 30518502.

4. Schuster SJ, Bishop MR, Tam CS, Waller EK, Borchmann P, McGuirk JP, Jäger U, Jaglowski S, Andreadis C, Westin JR, Fleury I, Bachanova V, Foley SR, Ho PJ, Mielke S, Magenau JM, Holte H, Pantano S, Pacaud LB, Awasthi R, Chu J, Anak Ö, Salles G, Maziarz RT. Tisagenlecleucel in Adult Relapsed or Refractory Diffuse Large B-Cell Lymphoma. N Engl J Med. 2019 Jan 3;380(1):45-56. eng. Epub 20181201. doi:10.1056/NEJMoa1804980. Cited in: Pubmed; PMID 30501490.

5. Abramson JS, Palomba ML, Gordon LI, Lunning MA, Wang M, Arnason J, Mehta A, Purev E, Maloney DG, Andreadis C, Sehgal A, Solomon SR, Ghosh N, Albertson TM, Garcia J, Kostic A, Mallaney M, Ogasawara K, Newhall K, Kim Y, Li D, Siddiqi T. Lisocabtagene maraleucel for patients with relapsed or refractory large B-cell lymphomas (TRANSCEND NHL 001): a multicentre seamless design study. Lancet. 2020 Sep 19;396(10254):839-852. eng. Epub 20200901. doi:10.1016/s0140-6736(20)31366-0. Cited in: Pubmed; PMID 32888407.

6. Lee DW, Gardner R, Porter DL, Louis CU, Ahmed N, Jensen M, Grupp SA, Mackall CL. Current concepts in the diagnosis and management of cytokine release syndrome. Blood. 2014 Jul 10;124(2):188-95. eng. Epub 20140529. doi:10.1182/blood-2014-05-552729. Cited in: Pubmed; PMID 24876563.

7. Schuster SJ, Tam CS, Borchmann P, Worel N, McGuirk JP, Holte H, Waller EK, Jaglowski S, Bishop MR, Damon LE, Foley SR, Westin JR, Fleury I, Ho PJ, Mielke S, Teshima T, Janakiram M, Hsu J-M, Izutsu K, Kersten MJ, Ghosh M, Wagner-Johnston N, Kato K, Corradini P, Martinez-Prieto M, Han X, Tiwari R, Salles G, Maziarz RT. Long-term clinical outcomes of tisagenlecleucel in patients with relapsed or refractory aggressive B-cell

lymphomas (JULIET): a multicentre, open-label, single-arm, phase 2 study. The Lancet Oncology. 2021;22(10):1403-1415. doi:10.1016/S1470-2045(21)00375-2.

8. Abramson JS, Palomba ML, Gordon LI, Lunning MA, Wang M, Arnason JE, Purev E, Maloney DG, Andreadis C, Sehgal AR, Solomon SR, Ghosh N, Kostic A, Kim Y, Ogasawara K, Dehner C, Siddiqi T. Two-Year Follow-up of Transcend NHL 001, a Multicenter Phase 1 Study of Lisocabtagene Maraleucel (liso-cel) in Relapsed or Refractory (R/R) Large B-Cell Lymphomas (LBCL). Blood. 2021;138(Supplement 1):2840-2840. doi:10.1182/blood-2021-148948.

9. Maude SL, Laetsch TW, Buechner J, Rives S, Boyer M, Bittencourt H, Bader P, Verneris MR, Stefanski HE, Myers GD, Qayed M, Moerloose BD, Hiramatsu H, Schlis K, Davis KL, Martin PL, Nemecek ER, Yanik GA, Peters C, Baruchel A, Boissel N, Mechinaud F, Balduzzi A, Krueger J, June CH, Levine BL, Wood P, Taran T, Leung M, Mueller KT, Zhang Y, Sen K, Lebwohl D, Pulsipher MA, Grupp SA. Tisagenlecleucel in Children and Young Adults with B-Cell Lymphoblastic Leukemia. New England Journal of Medicine. 2018;378(5):439-448. doi:doi:10.1056/NEJMoa1709866.

10. Hoover A, Reimche P, Watson D, Tanner L, Gilchrist L, Finch M, Messinger YH, Turcotte LM. Healthcare cost and utilization for chimeric antigen receptor (CAR) T-cell therapy in the treatment of pediatric acute lymphoblastic leukemia: A commercial insurance claims database analysis. Cancer Rep (Hoboken). 2024 Feb;7(2):e1980. The authors have no conflicts of interest to disclose. Epub 20240113. doi:10.1002/cnr2.1980. Cited in: Pubmed; PMID 38217445.

11. Di M, Potnis KC, Long JB, Isufi I, Foss F, Seropian S, Gross CP, Huntington SF. Costs of care during chimeric antigen receptor T-cell therapy in relapsed or refractory B-cell lymphomas. JNCI Cancer Spectr. 2024 Jul 1;8(4). K.C.P., J.B.L., and S.S. do not have any

conflict of interest to report. M.D. reports an honorarium from Intellisphere, consultancy fee from BeiGene, and research funding from Schrodinger and BeiGene. I.I. reports membership on an entity's board of directors or advisory committees at ADC Therapeutics, BEAM Therapeutics, and Epizyme; honoraria from Bayer; and speaker bureau from Kite. F.F. reports consultancy fees from Kyowa, Conjupro, Daiichi, Seagen, and Astex and speaker bureau from Seagen. C.P.G. reports research funding from the National Comprehensive Cancer Network Foundation (AstraZeneca), Genentech, and Johnson & Johnson. S.F.H. reports consultancy fees from Janssen, Pharmacyclics, AbbVie, AstraZeneca, Flatiron Health, Novartis, Seagen, Genentech, Merck, TG Therapeutics, ADC Therapeutics, Epizyme, Servier, and Thyme; research funding from Celgene, DTRM Biopharm, and TG Therapeutics; and honoraria form Pharmacyclics, AstraZeneca, and Bayer. doi:10.1093/jncics/pkae059. Cited in: Pubmed; PMID 39115391.

12. Wu J, Ghobadi A, Maziarz R, Patel K, Hsu H, Liu Z, Sheetz C, Kardel P, Fu C. Medicare Utilization and Cost Trends for CAR T Cell Therapies Across Settings of Care in the Treatment of Diffuse Large B-Cell Lymphoma. Adv Ther. 2024 Aug;41(8):3232-3246. Hill Hsu is employee of Kite Pharma, A Gilead Company and Christine Fu and James Wu are former employees of Kite Pharma, A Gilead Company. Zihao Liu, Peter Kardel, and Caitlin Sheetz are employees of ADVI Health Inc. Richard Maziarz reports serving as consultant for Autolous, Kite/Gilead, and Novartis, research support from Gamida, Allovir, OrcaBio, Kite/Gilead and Novartis, participating in a DSMB for Athersys, Novartis, Century Therapeutics and VorPharma and a patent with Athersys; no activity has conflict with the material of this article. Armin Ghobadi serving as consultant/advisory board for WUGEN, Atara pharmaceuticals, Celgene/BMS, CRISPR Therapeutics, Autolus, ADC Therapeutics, Nurix Therapeutics, serving as consultant and provide research support for Kite, a Gilead Company and Amgen Inc and Genentech. Krish Patel serve as consultant or received research

funding from AstraZeneca, Beigene, BMS, Loxo, Gentech/Roche, Kite, Century, CRISPR, Caribou, Curis, Sana, Fate Therapeutics, Xencor, ADC, Janssen, Nurix, Pfizer, Pharmacyclics. Epub 20240625. doi:10.1007/s12325-024-02917-7. Cited in: Pubmed; PMID 38916811.

13. Masucci L, Kuruvilla J, Sander B, Prica A, Wong WWL, Chan KKW. Real-world healthcare costs for patients treated with chimeric antigen receptor T-cell therapy in Canada. Leuk Lymphoma. 2025 Jun 17:1-9. Epub 20250617. doi:10.1080/10428194.2025.2518440. Cited in: Pubmed; PMID 40525245.

14. Heine R, Thielen FW, Koopmanschap M, Kersten MJ, Einsele H, Jaeger U, Sonneveld P, Sierra J, Smand C, Uyl-de Groot CA. Health Economic Aspects of Chimeric Antigen Receptor T-cell Therapies for Hematological Cancers: Present and Future. Hemasphere. 2021 Feb;5(2):e524. The authors have no conflicts of interest to disclose. Epub 20210128. doi:10.1097/HS9.0000000000000524. Cited in: Pubmed; PMID 33880433.

15. Ribera Santasusana JM, de Andres Saldana A, Garcia-Munoz N, Gostkorzewicz J, Martinez Llinas D, Diaz de Heredia C. Cost-Effectiveness Analysis of Tisagenlecleucel in the Treatment of Relapsed or Refractory B-Cell Acute Lymphoblastic Leukaemia in Children and Young Adults in Spain. Clinicoecon Outcomes Res. 2020;12:253-264. JMRS and CDH are, respectively, employed by Catalan Institute of Oncology-Hospital Germans Trias i Pujol and Hospital Universitari Vall d'Hebron. DML and NGM are employees of Oblikue Consulting, an independent contract health economic organization that received consultancy fees from Novartis Farmaceutica, S.A. to conduct this research. AAS and JG are employees of Novartis Farmaceutica, S.A., the marketing authorization holder for Kymriah(R) (tisagenlecleucel). JMRS reports grants and personal fees from AMGEN, Pfizer, Incyte, and Shire, and personal fees from Celgene, outside the submitted work. CHD reports personal fees and non-financial support from Novartis during the conduct of the study; and personal fees and non-financial support from Jazz Pharmaceuticals, Gilead, and Novartis, and non-financial support from

Alexion, outside the submitted work. The funding body was not involved in the study design, collection and interpretation of the data, or the decision to publish. The authors report no other conflicts of interest in this work. Epub 20200515. doi:10.2147/CEOR.S241880. Cited in: Pubmed; PMID 32523362.

16. Bastos-Oreiro M, de Las Heras A, Presa M, Casado MA, Pardo C, Martin-Escudero V, Sureda A. Cost-Effectiveness Analysis of Axicabtagene Ciloleucel vs. Tisagenlecleucel for the Management of Relapsed/Refractory Diffuse Large B-Cell Lymphoma in Spain. Cancers (Basel). 2022 Jan 21;14(3). Bastos-Oreiro M. has received conference and consulting fees from BMS, Celgene, Kite Pharma, Novartis, Roche and Takeda. Sureda A. has received conference and consulting fees from BMS, Celgene, Gilead, Janssen, MSD, Novartis, Roche, Sanofi and Takeda. Bastos-Oreiro B. and Sureda A. have received honoraria from Gilead for advocacy tasks related to this project. de las Heras A., Presa M., and Casado MA. are employees of Pharmacoeconomics & Outcomes Research Iberia, a consultant company specialised in economic evaluation of health technologies which has received unrestricted funding for development of the analysis. Pardo C. and Martin-Escudero V. are employees of Gilead Sciences Spain. Epub 20220121. doi:10.3390/cancers14030538. Cited in: Pubmed; PMID 35158805.

17. Li N, Zheng B, Cai H, Yang T, Hong Y, Liu M, Hu J. Cost-effectiveness analysis of axicabtagene ciloleucel vs. salvage chemotherapy for relapsed or refractory adult diffuse large B-cell lymphoma in China. Support Care Cancer. 2022 Jul;30(7):6113-6121. Epub 20220414. doi:10.1007/s00520-022-07041-2. Cited in: Pubmed; PMID 35419735.

18. Lin Z, Zuo C, Jiang Y, Su W, Yao X, Man Y, Wu Q, Xuan J. Cost-Effectiveness Analysis of Relmacabtagene Autoleucel for Relapsed or Refractory Large B-Cell Lymphoma in China. Value Health Reg Issues. 2023 Sep;37:41-48. Epub 20230518. doi:10.1016/j.vhri.2023.03.006. Cited in: Pubmed; PMID 37209540.

19. Wang XJ, Wang YH, Ong MJC, Gkitzia C, Soh SY, Hwang WYK. Cost-Effectiveness and Budget Impact Analyses of Tisagenlecleucel in Pediatric and Young Adult Patients with Relapsed or Refractory B-Cell Acute Lymphoblastic Leukemia from the Singapore Healthcare System Perspective. Clinicoecon Outcomes Res. 2022;14:333-355. XJW, YHW, MJCO, and CG are employees of Novartis. WYKH has received personal fees as Advisory Board member from Novartis and Gilead, and grant from CordLife for a clinical study. SYS has no financial or other interests for declaration here. The authors report no other conflicts of interest in this work. Epub 20220503. doi:10.2147/CEOR.S355557. Cited in: Pubmed; PMID 35535300.

20. Mallapaty S. Cutting-edge CAR-T cancer therapy is now made in India - at one-tenth the cost. Nature. 2024 Mar;627(8005):709-710. doi:10.1038/d41586-024-00809-y. Cited in: Pubmed; PMID 38514877.

21. Cliff ERS, Kelkar AH, Russler-Germain DA, Tessema FA, Raymakers AJN, Feldman WB, Kesselheim AS. High Cost of Chimeric Antigen Receptor T-Cells: Challenges and Solutions. Am Soc Clin Oncol Educ Book. 2023 Jun;43:e397912. doi:10.1200/EDBK_397912. Cited in: Pubmed; PMID 37433102.

22. Ran T, Eichmuller SB, Schmidt P, Schlander M. Cost of decentralized CAR T-cell production in an academic nonprofit setting. Int J Cancer. 2020 Dec 15;147(12):3438-3445. Epub 20200706. doi:10.1002/ijc.33156. Cited in: Pubmed; PMID 32535920.

23. Palani HK, Arunachalam AK, Kulkarni U, Yasar M, Venkatraman A, Palanikumar S, Radhakrishnan RN, Solomon M, Rajasekaran A, Bankar A, Datari PVR, Selvarajan S, Korula A, Dash P, Schneider D, Wirthlin L, Abraham A, George B, Mathews V. Safety, efficacy and total cost of point-of-care manufactured anti-CD19 CAR-T cell therapy in India: VELCART trial. Mol Ther Oncol. 2025 Jun 18;33(2):200977. The authors declare no competing interests. Epub 20250325. doi:10.1016/j.omton.2025.200977. Cited in: Pubmed; PMID 40248244.
