# Supplementary material for: Outcomes of point-of-care manufactured CAR T cell therapy for B cell acute lymphoblastic leukemia and non-Hodgkin lymphoma in Vietnam
Source: Mol Ther Oncol. 2026 Feb 13;34(1):201156. doi: 10.1016/j.omton.2026.201156 (PMC12969028; doi:10.1016/j.omton.2026.201156)
Supplement: Document S2. Article plus supplemental information [file mmc3.pdf]

Molecular Therapy
Oncology
Original Article

Outcomes of point-of-care manufactured CAR T cell therapy for B cell acute lymphoblastic leukemia and non-Hodgkin lymphoma in Vietnam

Liem Thanh Nguyen,[1,2,3,8] Duy D. Nguyen,[3] Quoc Khanh Bach,[4] Lan T.M. Dao,[1] Trang Thi Kieu Phan,[1] Hoang - Phuong Nguyen,[1] Hong-Nhung Dao,[1] Trang H. Pham,[1] Phuong T. Pham,[2] Hien T. Mai,[2] Viet Huong T. Pham,[3] Thanh Mai T. Nguyen,[2] Van Binh Le,[5] Nam Lam Phung,[5] Ngoc Quang Nguyen,[5] Michelle L. Hermiston,[6,7] Quynh Lan Phan,[5] Do Quang Trung Nguyen,[5] Lan Mai,[4] Quoc Nhat Nguyen,[4] and Van T. Hoang[1,8]

[1]Vinmec Research Institute of Stem Cell and Gene Technology, VinUniversity, Hanoi, Vietnam; [2]Department of Regenerative Medicine and Cell Therapy, Vinmec Healthcare System, Hanoi, Vietnam; [3]Vinmec Smart City International Hospital, Vinmec Health Care System, 2A Tay Mo Street, Hanoi 12009, Vietnam; [4]National Institute of Hematology and Blood Transfusion (NIHBT), 5 Pham Van Bach, Cau Giay District, Hanoi, Vietnam; [5]Vinmec Times City International Hospital, Vinmec Health Care System, 458 Minh Khai, Hanoi 11622, Vietnam; [6]College of Health Sciences, VinUniversity, Vinhomes Ocean Park, Gia Lam District, Hanoi 1310, Vietnam; [7]University of California, San Francisco, Helen Diller Comprehensive Cancer Center, 1450 3rd Streen, San Francisco, CA, USA

Chimeric antigen receptor (CAR) T cell therapy has transformed the treatment of relapsed/refractory (R/R) B cell acute lymphoblastic leukemia (ALL) and non-Hodgkin lymphoma (NHL), but access remains limited in resource-constrained settings. This phase I study evaluated the safety and feasibility of point-of-care (PoC) manufactured CD19-targeted CAR T cell therapy in Vietnamese patients. Between August 2023 and June 2025, 16 patients, eight with R/R ALL and eight with R/R NHL, were enrolled. All received fresh CAR T cells produced on-site using the CliniMACS Prodigy system, with a median dose of 1.9 $\times$ 10^6 CAR T cells/kg (range, 0.83–2.17 $\times$ 10^6). Cytokine release syndrome (CRS) occurred in 13 patients (12 with grade 1–2, one with grade 3), and grade 1 neurotoxicity was observed in two patients. In ALL, the complete remission (CR) rates were 100% on day 30, 75% on day 90, and 62.5% on day 180. Patients with NHL showed CR rates of 87.5% on both day 90 and day 180. The estimated 1-year progression-free survival rates were 62.5% (95% confidence interval [CI]: 36.5%–100%) for ALL and 87.5% (95% CI: 67.3%–100%) for NHL. PoC manufactured CD19 CAR T cells demonstrated manageable toxicity and encouraging early efficacy in Vietnamese patients with R/R ALL and NHL. This model offers a cost-effective strategy for delivering advanced therapy in resource-limited settings.

INTRODUCTION

Cure rates for acute lymphoblastic leukemia (ALL) and non-Hodgkin lymphoma (NHL) have improved substantially.[1,2] However, the prognosis for patients with relapsed/refractory (R/R) disease remains poor. Before the introduction of chimeric antigen receptor (CAR) T cell therapy into clinical practice, the Acute Leukemia Working Party of the European Society for Blood and Marrow Transplantation (EBMT) reported a median time to relapse of less than 6.9 months following hematopoietic stem cell transplantation, with a median survival after relapse of only 5.5 months in ALL.[3] Although initial responses to first-line chemotherapy in B cell NHL are generally favorable, historical data from the pre-CAR T cell era demonstrate high rates of treatment failure: approximately 15%–20% of patients with diffuse large B cell lymphoma (DLBCL) exhibited refractory disease, and an additional 20%–30% experienced relapse within 2 years of initial therapy.[4] For refractory DLBCL, response rates were only 26%, with a median overall survival (OS) of 6.3 months.[5]

CAR T cell therapy has revolutionized the treatment of patients with R/R ALL and NHL. So far, seven CAR T cell products have been approved by the U.S. Food and Drug Administration (FDA), including five CD19-targeted therapies for use in R/R B cell ALL, DLBCL, follicular lymphoma, and/or mantle cell lymphoma.[6–9] Tisa-cel has demonstrated complete remission (CR) rates of 81%–87% in pediatric and young adult patients with R/R B cell ALL across clinical trials and real-world studies. In adult patients with R/R B cell ALL, Brexu-cel has achieved CR rates ranging from 71% to 81%. For DLBCL, three CAR T cell

Received 13 November 2025; accepted 11 February 2026;
https://doi.org/10.1016/j.omton.2026.201156.

[8]These authors contributed equally

Correspondence: Liem Nguyen Thanh, PhD, Vinmec Research Institute of Stem Cell and Gene Technology, College of Health Sciences, VinUniversity, Vinhomes Ocean Park, Gia Lam District, Hanoi 1310, Vietnam.
E-mail: liem.nt@vinuni.edu.vn
Correspondence: Van T. Hoang, PhD, Vinmec Research Institute of Stem Cell and Gene Technology, College of Health Sciences, VinUniversity, Vinhomes Ocean Park, Gia Lam District, Hanoi 1310, Vietnam.
E-mail: van.ht@vinuni.edu.vn

© 2026 The Authors.
Published by Elsevier Inc. on behalf of The American Society of Gene and Cell Therapy.
This is an open access article under the CC BY license (http://creativecommons.org/licenses/by/4.0/).

therapies are currently approved, with CR rates of 37%–54% when used as third-line therapy and 65%–66% when administered as second-line therapy.[7,8]

However, all approved CAR T cell products are centrally manufactured, leading to high production costs and long turnaround times due to the need for shipping cells to and from specialized facilities.[10] This poses significant barriers to access, particularly for patients in countries outside of Europe and North America, such as Vietnam. To address these challenges, some centers have successfully implemented point-of-care (PoC) CAR T cell manufacturing to reduce costs and delays.[11,12] This study is the first to evaluate the safety and preliminary efficacy of CAR T cell therapy using PoC-manufactured CAR T cells (VINCAR-T) in Vietnamese patients with R/R NHL and ALL.

RESULTS

Baseline demographics and patient characteristics

Eighteen patients with R/R B cell NHL and ALL met the inclusion and exclusion criteria. Two patients were excluded from the final analyses because their CAR T cell product did not meet release criteria, and the remaining 16 patients comprised the evaluable cohort (Figure S1A). Baseline demographic and clinical characteristics are summarized in Table 1. The median age was 14 years (range, 5–50 years) for ALL and 41 years (range, 32–55 years) for NHL. The median number of prior lines of therapy was 3 (range, 1–3) for ALL and 3 (range, 2–5) for NHL. At baseline, all eight patients with ALL had relapsed disease, of which four were refractory and four had incomplete responses to their last regimen. Bone marrow (BM) blast counts exceeding 5% were observed in two patients. In the NHL cohort, three patients had refractory disease. The remaining five patients with NHL experienced disease relapse, with one classified as refractory and four exhibiting incomplete responses to the most recent line of treatment. One patient with NHL presented with bulky disease, with a tumor size of 117 × 81 mm.

Table 1. Baseline demographics and disease characteristics of patients treated with anti-CD19 CAR T cell therapy

Clinical parameters	ALL (*n* = 8)	NHL (*n* = 8)
Age, median (range), years	14 (5–50)	41 (32–55)
Gender, n (%)		
Female	5 (62.5%)	4 (50.0%)
Male	3 (37.5%)	4 (50.0%)
Hemoglobin, median (range), gm%	11.0 (8.2–12.9)	12.9 (8.6–13.8)
White blood cells, median (range), G/l	5.2 (2.9–6)	4.3 (2.4–6.5)
Lymphocytes, median (range), G/l	1.0 (0.5–2.1)	1.1 (0.79–2.6)
Platelets, median (range), G/l	275 (152–315)	170 (107–227)
Liver Function Test		
Total bilirubin, median (range), μmol/L	8.1 (6.4–19.8)	9.2 (5.8–16.5)
SGOT, median (range), U/L	29.2 (19–55)	25.7 (16.1–52.2)
SGPT, median (range), U/L (range)	27.8 (8.4–50.8)	18.9 (8.4–59.8)
Serum creatinine, median (range), μmol/L	41.0 (27.0–58.0)	75.0 (43.0–115.0)
Serum LDH, median (range), U/L	221.5 (124–592)	206 (14.2–1455)
Patients with Additional Comorbidities		
Cardiovascular disease	0	1
Hepatitis B	1	2
Primary indication, n (%)		
Refractory relapse	4 (50.0%)	1 (12.5%)
Non-refractory relapse	4 (50.0%)	4 (50.0%)
Refractory	0 (0%)	3 (37.5%)
Line	3 (1–3)	3 (2–5)
BM blast (>5%)	2/8	NA
BM blast (>2%)	3/8	NA
Bulky lymphoma (i.e., >7.5 cm)	NA	1/8
CNS or other extramedullary involvement before lymphodepletion	Nil	Nil
Length of hospitalization (days)	43 (31–74)	32 (30–34)

Abbreviations are as follows: ALL, acute lymphoblastic leukemia; LDH, lactate dehydrogenase; NHL, non-Hodgkin lymphoma; SGOT, serum glutamic-oxaloacetic transaminase; SGPT, serum glutamate pyruvate transaminase.

VinCART cells were manufactured on-site using the CliniMACS Prodigy system, with a vein-to-vein time of 13 days for the first two patients and 9 days for the remaining patients. Characteristics of the VinCART cells are presented in Table 2. Patients received lymphodepleting chemotherapy with cyclophosphamide and fludarabine, followed by intravenous infusion of CAR T cells at a median dose of 1.90×10^6 cells/kg body weight (range, $0.83–2.17 \times 10^6$ cells/kg body weight) (Figure S1B).

Safety

There were no deaths attributed to CAR T cell therapy. Patients with ALL had a total of 162 adverse events (AEs) and no serious AEs (SAEs). One patient with NHL experienced an SAE, and the NHL cohort had a total of 139 AEs (Table 3).

Cytokine release syndrome (CRS) occurred in 13 of 16 patients (81.2%), and immune effector cell-associated neurotoxicity syndrome (ICANS) was observed in two of 16 patients (12.5%) (Table 3). Among patients with ALL, CRS was restricted to grade 1–2 events, occurring in 6 of 8 patients (75.0%), with no grade ≥3 CRS reported. ICANS in the ALL cohort was infrequent and mild, with one patient (12.5%) experiencing grade 1 ICANS. In the NHL cohort, CRS was reported in 7 of 8 patients (87.5%), including six patients (75.0%) with grade 1–2 CRS and one patient (12.5%) with grade 3 CRS. The patient who developed grade 3 CRS also experienced grade 1 ICANS (12.5%) and required admission to the intensive care unit. ICANS was not observed in the other patients.

For CRS management, tocilizumab was administered to 6 of 8 patients with ALL (75.0%) and 3 of 8 patients with NHL (37.5%), with a median time to first administration of 1.5 days

Table 2. Characterization and quality control parameters of CD19 CAR T cell products

ID	Total cell culture day	%CD3+ (%)	%CAR+ (%)	CAR-T (million cells/kg)	Viability (%)	Mycoplasma	Endotoxin (EU/kg)	Bacteria/ fungi	VCN (copies per cell)	Potency (%) E:T = 5:1
BN01	12	99.17	52.82	2.09	90.22	Neg.	<0.05	Neg.	2.46	99.99
BN03	12	99.49	67.92	1.84	95.45	Neg.	<0.05	Neg.	2.25	99.8
BN06	8	99.21	37.95	1.75	99	Neg.	<0.05	Neg.	1.82	99.78
BN07	8	99.22	34.5	1.33	91.44	Neg.	0.086	Neg.	1.69	99.97
BN09	8	99.33	41.96	2.09	98.33	Neg.	<0.05	Neg.	2.01	99.99
BN10	8	99.24	49.64	1.61	98.47	Neg.	<0.05	Neg.	2.14	99.95
BN12	8	99.23	43.34	1.97	97.91	Neg.	<0.05	Neg.	1.82	99.97
BN17	8	99.21	33.76	2.05	98.64	Neg.	<0.1	Neg.	1.96	99.91
BN05	8	99.39	43.25	1.85	97.46	Neg.	<0.05	Neg.	1.8	99.9
BN08	8	98.7	20.2	0.83	92.17	Neg.	<0.05	Neg.	1.87	99.96
BN11	8	99.49	40.12	1.81	98.41	Neg.	<0.05	Neg.	1.82	99.96
BN13	8	99.4	25.28	2.09	98.14	Neg.	<0.05	Neg.	1.73	99.93
BN14	8	99.17	41.13	1.6	95.25	Neg.	<0.05	Neg.	1.78	99.94
BN15	8	99.71	17.24	2.17	96.29	Neg.	<0.05	Neg.	3.44	99.83
BN16	8	99.36	42.98	2	98.18	Neg.	<0.05	Neg.	2.1	99.94
BN18	8	99.16	32.8	1.94	98.46	Neg.	<0.053	Neg.	2.04	99.66
Median (range)	**8 (8,12)**	**99.24 (98.70–99.71)**	**40.63 (17.24–67.92)**	**1.90 (0.83–2.17)**	**98.03 (90.22–99.00)**	**NA**	**NA**	**NA**	**1.92 (1.69–3.44)**	**99.94 (99.66–99.99)**

Abbreviations are as follows: E, effector; NA, not applicable; neg., negative; T, target; VCN, vector copy number.

post-infusion (range, 1–8 days) (Table 3). Patients receiving tocilizumab required a median of 3 doses (range, 1–17). Dexamethasone was administered to two patients with ALL (25.0%) and one patient with NHL (12.5%), with dosing frequencies of 1, 4, and 11 doses, respectively (Table 3).

Clinical responses

In the cohort of ALL patients, the CR rates were 100% at day 30 post-treatment, 75.0% at day 90, and 62.5% at day 180, and these rates remained stable through the most recent follow-up assessments (Table S1). During a mean follow-up of 12.9 months (95% confidence interval [CI]: 7.8–17.9 months) for the ALL group, three patients in the ALL cohort died of disease progression (Figure 1A). The estimated 1-year progression-free survival (PFS) rate for the ALL group was 62.5% (95% CI: 36.5%–100%), and the median PFS was not reached.

Patients with NHL demonstrated a CR rate of 87.5% at both day 90 and day 180, with this rate persisting through subsequent follow-up evaluations (Table S1). The mean follow-up duration was 10.4 months (95% CI: 8.07–12.79 months). One patient experienced disease progression, resulting in death at 7.4 months post-treatment (Figure 1A). The 1-year PFS rate in this group was 87.5% (95% CI: 67.3%–100%), while the median PFS was not reached. Kaplan-Meier plots illustrating OS and PFS for both ALL and NHL cohorts are presented in Figures 1B–1E.

Correlates of response: $CD19^+$ B cells and CAR T cells in patients

B cells and CAR T cells were monitored to evaluate the therapeutic response. In patients with ALL, $CD19^+$ B cell levels declined following conditioning therapy and CAR T cell infusion (Figure 2A). In parallel, CAR T cell concentrations increased progressively, peaking on days 10–14 post-infusion in all patients (Figure 2B). Similarly, patients with NHL exhibited rapid depletion of B cells, corresponding with CAR T cell expansion (Figures 2C and 2D). The median peak CAR T cell count was 658.9 cells/μl (range: 54.0–1680.6) in patients with ALL and 1278.2 cells/μl (range: 82.9–6529.3) in those with NHL, representing a median of 59.55% (range: 9.17–85.30) and 52.07% (range: 18.22–91.06) of the CD3+ T cell subset in flow cytometry analysis, respectively (Figure 2E). CAR T cells persisted at lower levels through the final follow-up on day 180, with a median of 2.99 cells/μl (n = 6, range: 0.80–11.12) in patients with ALL and 3.32 cells/μl (n = 8, range: 0.25–47.36) in patients with NHL (Figure 2F). Data were not available for two patients who relapsed. Furthermore, quantitative PCR analysis confirmed an initial increase in CAR vector copy number (VCN) in peripheral blood (PB) following infusion, followed by a gradual decline over time in patients with ALL and more sustained persistence in NHL cases (Figures 2G and 2H).

The infused CAR T cell products exhibited a balanced CD4:CD8 ratio. However, following infusion, the CAR T cell population became skewed toward predominantly $CD8^+$ T cells, as observed in both the PB and BM of patients with ALL (Figures 3A and 3B) and in the PB

Table 3. AEs and SAEs

AE/SAE	ALL	NHL	Notes
SAE	0	1	
AE	162	139	
Unrelated to the CT intervention	7 (4.3%)	11 (7.9%)	flank pain, lymph node pain, rash, abdominal pain, anal pain, hypercalcemia, hypercholesterolemia
Unlikely related to the CT intervention	41 (25.3%)	30 (21.6%)	bone pain, hypocalcemia, decreased albumin, hypokalemia, hypocalcemia, hyponatremia, vomiting, increased uric acid, hypertriglyceridemia, diarrhea
Possibly related to the CT intervention	48 (29.6%)	43 (30.9%)	headache, decreased fibrinogen, fever, elevated bilirubin, increased INR, elevated creatinine, anemia, elevated LDH, increased liver enzymes.
Probably related to the CT intervention	58 (35.8%)	48 (34.5%)	leukopenia, neutropenia, thrombocytopenia
Definitely related to the CT intervention	8 (4.9%)	7 (5.0%)	CRS, ICANS.
**CRS/ICANS**	**Grade**	**ALL**	**NHL**
CRS	Grade 1 and 2	6 (75.0%)	6 (75.0%)
	Grade ≥3	0 (0%)	1 (12.5%)
ICANS	Grade 1 and 2	1 (12.5%)	1 (12.5%)
	Grade ≥3	0 (0%)	0 (0%)
Medications	Tocilizumab	6 (75.0%)	3 (37.5%)
	Dexamethasone	2 (25.0%)	1 (12.5%)

AEs and SAEs occurred following VinCART therapy.
Abbreviations are as follows: AE, adverse event; ALL, acute lymphoblastic leukemia; CRS, cytokine release syndrome; CT, cell therapy; ICANS, immune effector cell-associated neurotoxicity syndrome; NHL, non-Hodgkin lymphoma; SAE, serious adverse event.

of patients with NHL (Figure 3C). Both patients with ALL and patients with NHL demonstrated a phenotypic shift from central memory T cells to effector memory T cells around days 10–14 post-infusion (Figures 3D–3I).

IgG levels

Serum IgG levels declined in all patients, consistent with B cell depletion. Median IgG concentrations decreased from 990.5 mg/dL at baseline (n = 16; range: 524.0–1774.9 mg/dL) to 314.0 mg/dL by day 180 (n = 13; range: 120.0–1170.0 mg/dL) (Figure 4A).

Hypogammaglobulinemia, defined as serum IgG < 400 mg/dL, was observed post-treatment in 6 of 8 patients with ALL and 5 of 8 patients with NHL at least at one time point during the 180-day follow-up. On day 180, hypogammaglobulinemia persisted in 4 of 5 patients with ALL and 4 of 7 patients with NHL who remained in CR, as well as in one patient with NHL experiencing disease progression. Unfortunately, IgG data at day 180 were unavailable for three patients with ALL who relapsed. Patients with hypogammaglobulinemia received intravenous immunoglobulin (IVIG) therapy (0.4 mg/kg body weight per dose) to reduce infection risk.

Cytokine quantification

Serum cytokines were analyzed on days 3, 7, 14, 21, 30, 60, 90, and 180 after CAR T cell infusion. Levels of IL-6 and IFN-γ were elevated on days 3–7 in a subset of individuals following CAR T cell infusion, whereas other cytokines, including GM-CSF, TNF-α, IL-4, and IL-17A, remained unchanged (Figure S2). Among patients who developed CRS, IL-6 levels significantly increased by day 3 post-infusion relative to baseline (p = 0.01; Figure 4B). IFN-γ levels were higher on day 7; however, this change did not reach statistical significance (Figure 4C).

A positive association was observed between peak body temperature and both the highest IL- 6 concentration (Spearman $\rho = 0.90$, $p = 1.8 \times 10^{-6}$) and peak CAR T cell levels (Spearman $\rho = 0.50$, $p = 0.047$), indicating that higher febrile responses coincided with increased cytokine release and CAR T cell expansion (Figures 4D and 4E).

Patients were stratified into two groups: those who maintained CR at day 180 and those who experienced refractory disease or relapse (R/R) to explore a potential correlation between these parameters and clinical outcomes. The baseline characteristics and pharmacodynamic parameters of these groups are represented in Table S2. Notably, the R/R group exhibited a higher initial disease burden, as reflected by increased blast frequency in the ALL cohort and larger tumor size in patients with NHL (Figure S3; Table S2). This group also tended to exhibit greater CAR T cell expansion, higher body temperature, and elevated IL-6 levels than the CR group (Figure S3; Table S2). However, no statistically significant differences were found, potentially due to the limited sample size of this cohort.

Treatment failure and relapse

In the ALL cohort, three patients presented with a disease burden of ≥4% blasts (4%, 5%, and 10%, respectively) in the BM prior to treatment. All three patients achieved CR by day 30 post-infusion. However, two children (BN03 and BN07) with pre-treatment disease burdens of 4% and 10% experienced relapse by day 91 and 83, respectively, and both subsequently succumbed to the disease on days 510 and 106, respectively (Table S1). An additional relapse occurred in an adult patient with ALL (BN12) on day 126 (Table S1). In the NHL cohort, one patient, presenting with a tumor mass measuring 117 × 81 mm, demonstrated refractory disease and died on day 221 (Table S1). The relationship between tumor load, peak CAR T cell and IL-6 levels, and clinical responses is detailed in Table S2.

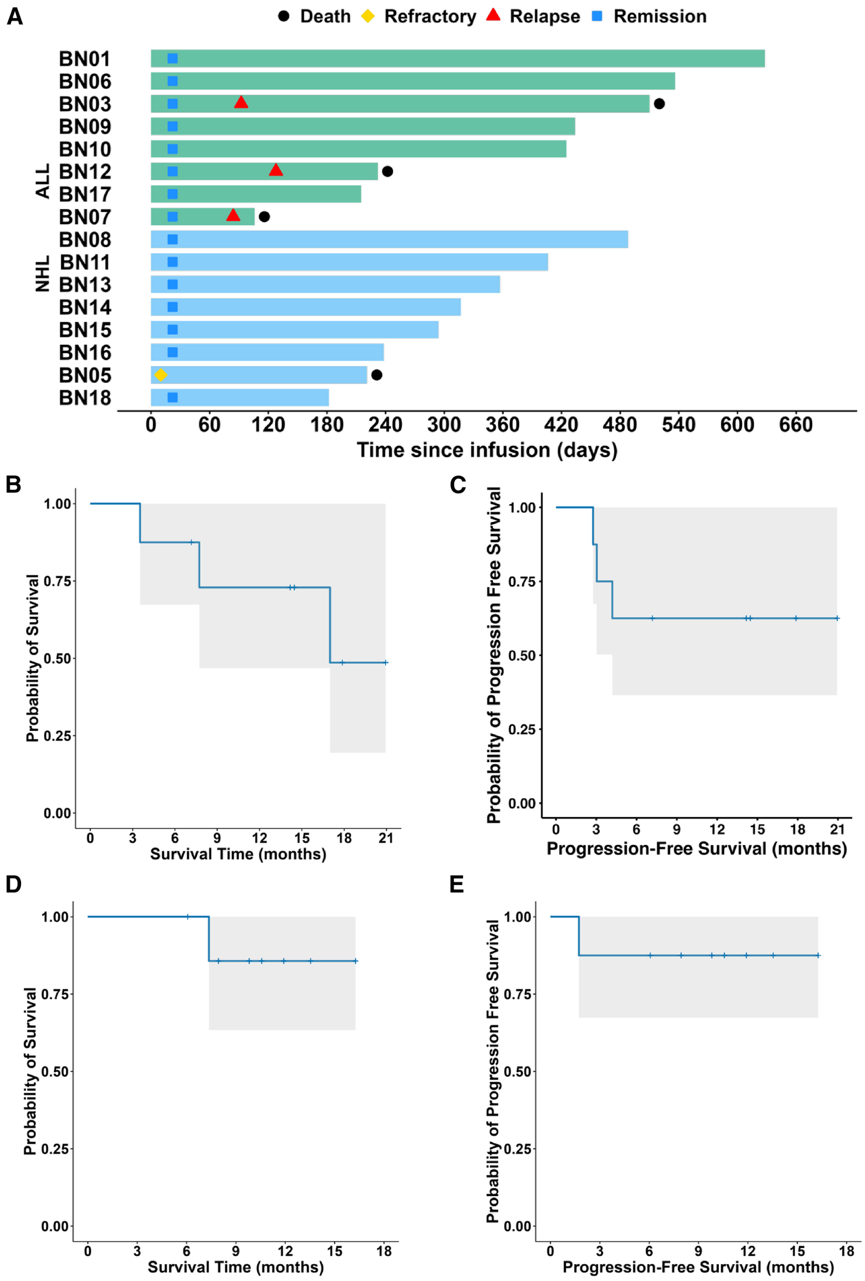

Figure 1. Overall and progression-free survival of ALL and NHL

(A) Clinical outcomes of individual patients. Each horizontal bar represents a single patient, grouped by diagnosis: B cell ALL (top, green) and NHL (bottom, blue).
(B and C) OS and PFS of the ALL cohort (*n* = 8), respectively. The gray area denotes the 95% confidence interval of the Kaplan-Meier survival estimate.
(D and E) OS and PFS of patients with NHL (*n* = 8), respectively. The gray area denotes the 95% confidence interval of the Kaplan-Meier survival estimate.
ALL, acute lymphoblastic leukemia; NHL, non-Hodgkin lymphoma; PFS, progression-free survival; OS, overall survival.

All three patients with relapsed ALL exhibited $CD19^{-}$ disease recurrence. In patient BN03, 69.3% $CD45^{dim}SSC^{low}$ blasts expressed CD19 prior to apheresis, while 38.9% lacked this marker. Both blast populations disappeared at the time of CR on day 30. At relapse, 95.5% blasts were $CD19^{-}$ (Figure 5A). Similarly, patient BN07 had 62.9% $CD19^{+}$ and 36.3% $CD19^{-}$ blasts prior to apheresis, shifting to 93.2% $CD19^{-}$ blasts at relapse (Figure 5B). Patient BN12 also demonstrated loss of CD19 expression in the blast population at relapse.

Interestingly, the BM and PB of the relapsed patients displayed variable CAR T cell levels, as determined by flow cytometry and quantitative PCR over time (Figures 5C and 5D, respectively). At relapse on day 90, patient BN03 exhibited low levels of CAR T cells (Figures 5C and 5D), which corresponded with an elevated concentration of B cells in the BM (Figure 5E). In contrast, CAR T cells persisted in patients BN07 and BN12 at day 90 (Figures 5C and 5D), corresponding with profound B cell aplasia (Figure 5E), indicating sustained activity and consistent with CD19-negative relapses.

B cell recovery

In the ALL cohort, B cell recovery (>50,000 B cells/mL) was observed in two patients who remained in CR. These patients exhibited 0% blast frequency in the BM at pre-apheresis (Table S1). Patient BN10 showed re-emergence of peripheral B cells by day 60 following CAR T cell infusion (Figures 2A and S4A), while patient BN06 recovered by day 180 (Figures 2A and S4B). Notably, these patients exhibited low to no detectable CAR T cells as B cells recovered, as measured by flow cytometry (Figures S4C and S4D) and quantitative PCR (Figures S4E and S4F). B cell differentiation was analyzed in BN10 (Figure S5A). By day 7 post-infusion, the patient exhibited profound B cell aplasia. However, by day 90, PB analysis showed evidence of B cell reconstitution, with populations including $CD19^{+}CD20^{+}CD38^{+}$ pre-B cells, $CD19^{+}CD20^{+}IgD^{+}$ activated B cells, $CD19^{+}CD20^{+}CD27^{+}$ memory B cells, and $CD38^{+}CD138^{+}$ plasma cells. A concurrent BM sample collected on day 90 revealed a similar pattern of B cell differentiation, though with a predominance of immature $CD19^{+}CD20^{-}CD38^{+}$ pro-B cells (Figure S5B). The remaining six patients exhibited sustained B cell aplasia, with no evidence of B cell recovery by day 180 (Figure 2A). In the NHL cohort (*n* = 8), all patients demonstrated

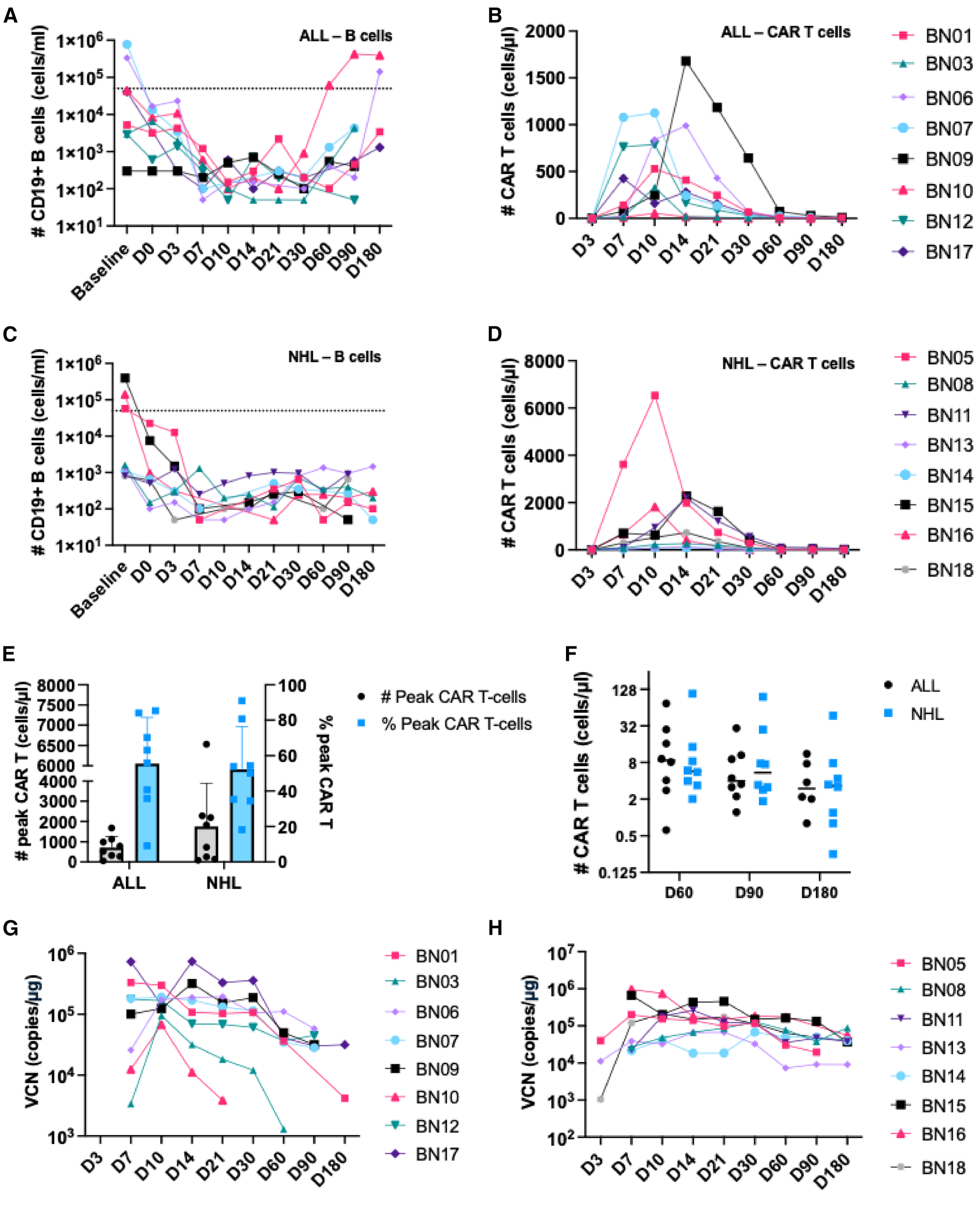

Figure 2. Analysis of B cells and CAR T cells in patients with ALL and NHL

(A and B) Flow cytometry analysis of PB samples from patients with ALL from baseline to day 180, quantifying B cells (A) and CAR T cell numbers (B).

(C and D) Flow cytometry analysis of B cells (C) and CAR T cell numbers (D) in patients with NHL. The dotted line represents the threshold for B cell aplasia, defined as fewer than 50 B cells/μL.

(E) The highest observed numbers and frequencies of CAR T cells in the PB of patients with ALL and NHL.

(F) CAR T cell numbers at days 60, 90, and 180 in patients with ALL and NHL.

(G and H) Quantitative analysis of CAR VCN in T cells from the PB of patients with ALL (G) and NHL (H).

ALL, acute lymphoblastic leukemia; NHL, non-Hodgkin lymphoma; PB, peripheral blood; VCN, vector copy number.

The data in E are represented as mean ± SD.

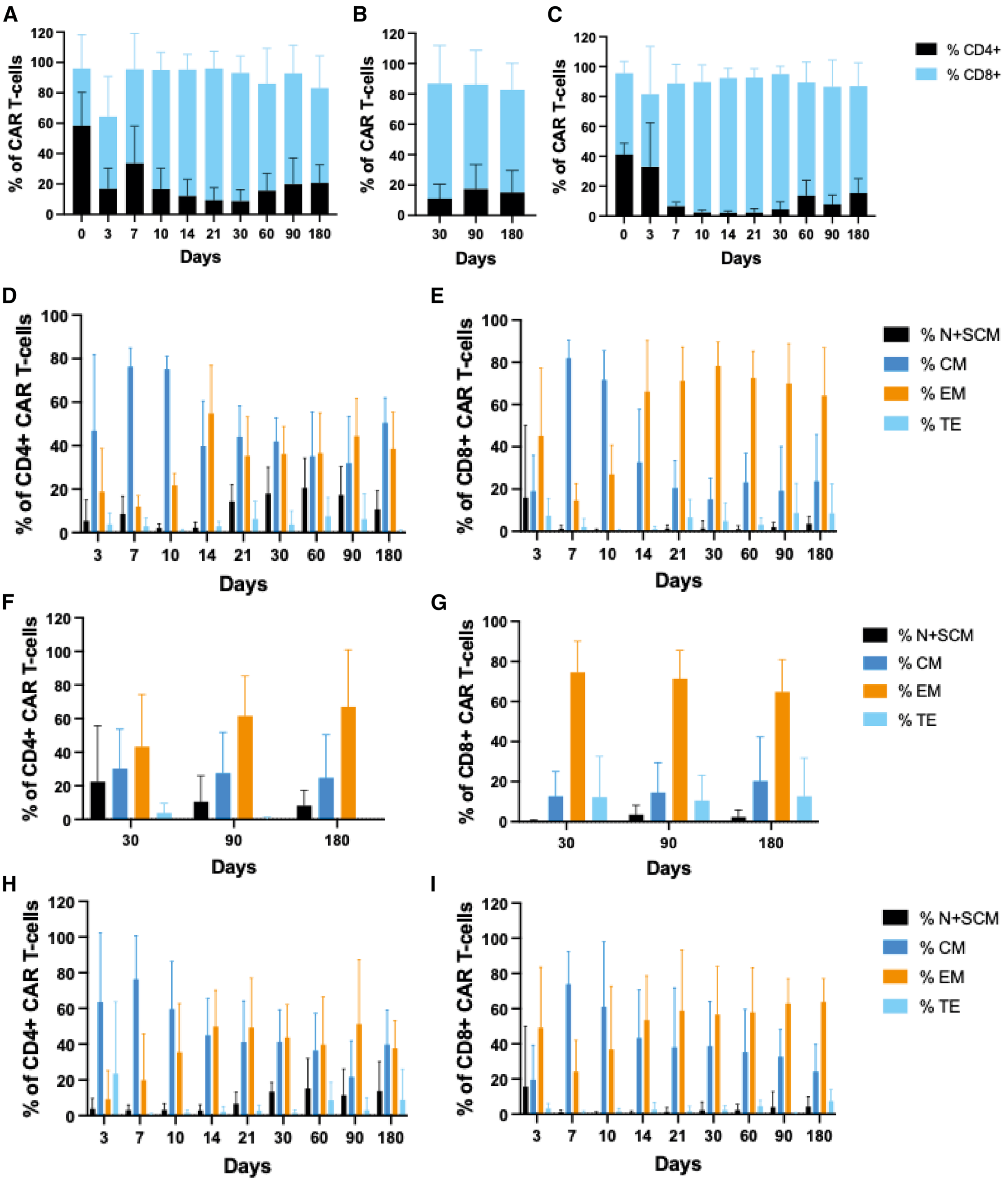

Figure 3. Phenotypic characterization of CAR T cells in patients

(A and B) Distribution of CD4+ and CD8+ subsets of CAR T cells in PB (A) and BM (B) of patients with ALL.

(C) CD4+ and CD8+ subsets of CAR T cells in PB of patients with NHL.

(D and E) Longitudinal immunophenotypic profiling of $CD4^+$ (D) and $CD8^+$ (E) CAR T cells in the PB of patients with ALL from day 3 to day 180 post-infusion.

(F and G) BM analysis of $CD4^+$ (F) and $CD8^+$ (G) CAR T cells in patients with ALL at days 30, 90, and 180 post-infusion.

(H and I) Evaluation of $CD4^+$ (H) and $CD8^+$ (I) CAR T cells in PB of patients with NHL over the 180-day follow-up period. ALL, acute lymphoblastic leukemia; BM, bone marrow; NHL, non-Hodgkin lymphoma; PB, peripheral blood; VCN, vector copy number.

The data are represented as mean ± SD.

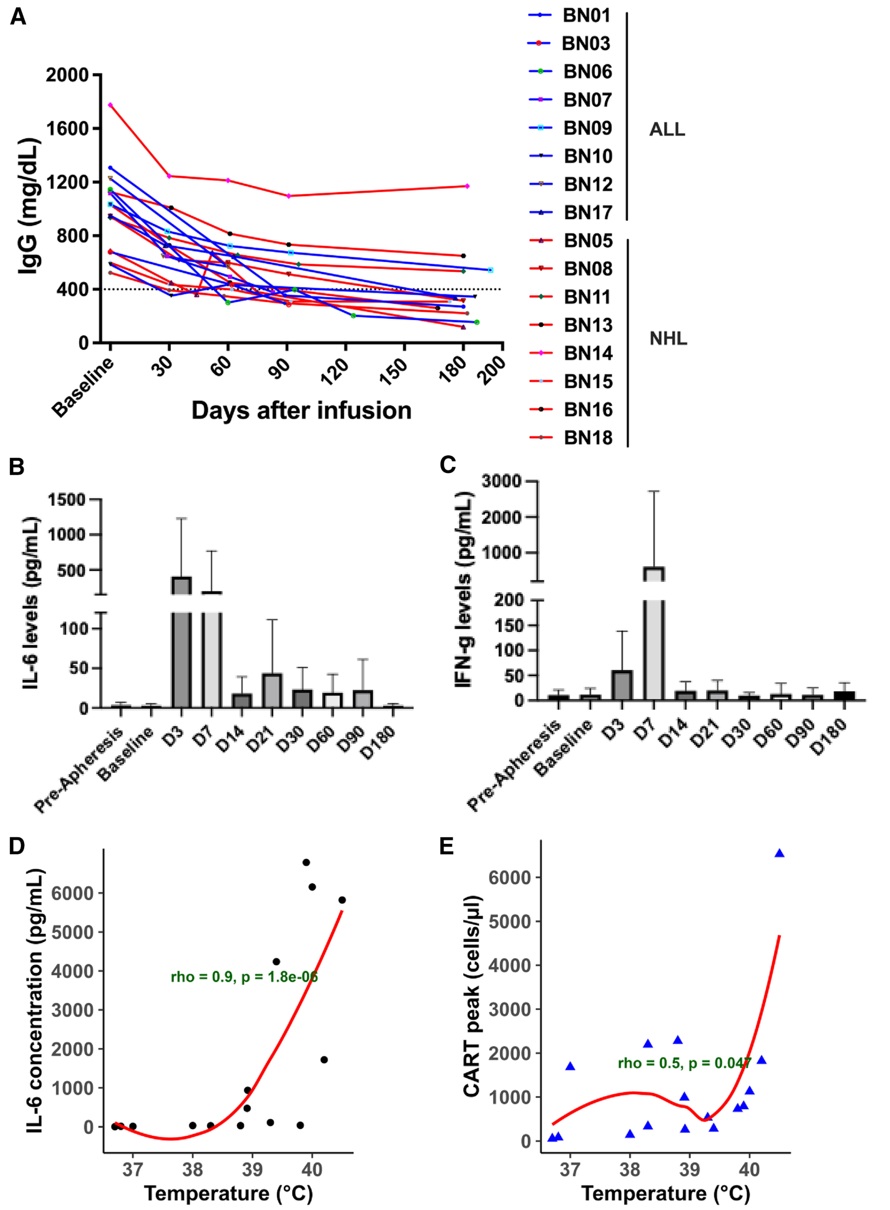

Figure 4. IgG and cytokine levels after CAR T cell infusion

(A) Longitudinal assessment of IgG levels in patients after CAR T cell infusion.
(B and C) Plasma concentrations of IL-6 (B) and IFN-γ (C) in patients treated with CAR T cells.
(D) Correlation between peak IL-6 levels and patients' maximum recorded temperature.
(E) Correlation between patients' maximum recorded temperature and peak CAR T cell expansion.
The data in B and C are represented as mean ± SD.

persistent B cell aplasia throughout the observation period, with no recovery detected up to day 180 post-infusion (Figure 2C).

Cost for VinCART therapy

The estimated total cost per patient receiving VinCART therapy in our study was approximately US$120,000, which is significantly lower than that of commercial products in the United States, Canada, the European Union, Singapore, and China (Figure S6; Table S3). This included US$80,000 attributed to the manufacturing of the CAR T cell product, which involved reagent and quality control costs of US$39,100, lentiviral vector cost of US$33,000, personnel expenses of US$2,300, and facility costs of US$5,600. This is comparable to other PoC CAR T products manufactured in Spain, Germany, and India, the latter with estimated costs that excludes the viral vector (Table S3). The remaining US$40,000 covered clinical care-related costs, including hospitalization, laboratory testing, and supportive medications administered during treatment and post-infusion monitoring. Two-thirds of the clinical care-related costs were allocated to inpatient expenses, while the remaining one-third covered outpatient and follow-up expenses.

DISCUSSION

CAR T cell therapy has led to impressive clinical outcomes in hematological cancers; however, technological and manufacturing challenges significantly limit its applications, especially in low- and middle-income countries (LMICs) and resource-limited settings. Decentralized CAR T cell manufacturing can pave the way to accelerate the implementation of CAR T cell therapy, especially in developing countries.[13] Our results demonstrate that VinCART can be successfully manufactured on-site, ensuring high product quality and a rapid turnaround time.

VinCART products underwent rigorous quality control testing in accordance with regulatory guidance from the U.S. FDA and the European Pharmacopoeia.[14–17] Quality assessments included evaluations of safety (sterility, mycoplasma testing, and VCN); purity (T cell and CAR T cell content and contaminating cell populations); and potency (CAR expression and *in vitro* cytotoxic activity). Despite the absence of internationally harmonized release criteria,[18] our PoC manufacturing process adhered to the standards proposed by Delgado et al. in the EBMT/ European Haematology Association (EHA) CAR T cell Handbook.[19] We demonstrated high purity, cell viability, cytotoxicity, and reasonable VCN in our CAR T cell products.

Safety

In our cohort of 16 patients, CAR T cell therapy demonstrated a favorable safety and efficacy profile in the treatment of both ALL

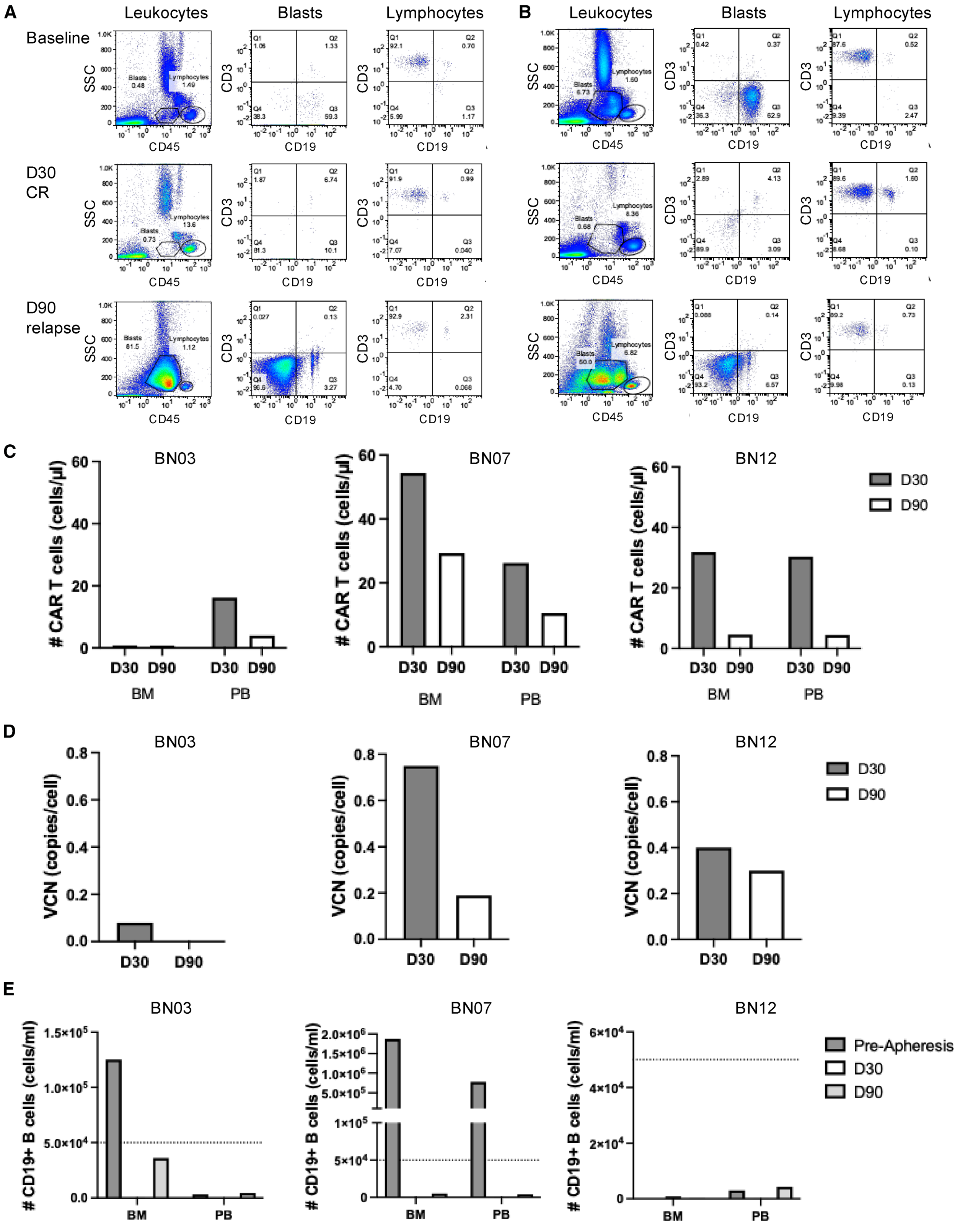

(legend on next page)

and NHL. No treatment-related deaths were observed. Notably, only one case of grade 3 CRS occurred in the NHL group. ICANS was infrequent and mild across both disease cohorts, with only one grade 1 case reported in each. These safety outcomes align favorably with findings from previously published clinical studies (Table S4).[11,20,21]

CRS is a frequently observed complication associated with CAR T therapy. Despite its prevalence, optimal strategies for early detection and the appropriate timing for therapeutic intervention remain matters of ongoing debate. Fever is widely regarded as a hallmark clinical feature indicative of early CRS onset; however, distinguishing CRS from infectious etiologies remains challenging in clinical practice.[22] Tocilizumab is generally recommended for grade 1 CRS if fever persists beyond 72 h following the exclusion of infection or at grade 2 in patients with significant comorbidities or advanced age.[22,23] Early administration of tocilizumab is also advised in cases in which two temperature readings $\geq$38.5°C occur within a 24-h period, spaced at least 4 h apart, or in patients with CRS of grade $\leq$2, in order to mitigate progression to more severe grades, as reported elsewhere.[24,25]

In our cohort, CRS was detected by the onset of fever in accordance with international guidelines.[23] Furthermore, real-time monitoring of IL-6 upon fever occurrence enabled prompt recognition of cytokine elevation, based on a strong correlation observed between peak body temperature and IL-6 concentrations (Spearman ρ = 0.90). This facilitated early administration of tocilizumab, and such rapid intervention might have contributed to the overall mitigation of CRS severity, with only one patient developing grade 3 CRS and no instances of grade 4 or 5 events in our cohort.

Efficacy

The data revealed high CR rates in both ALL and NHL cohorts, underscoring the robust antitumor efficacy of CAR T cell therapy. In ALL, the CR rate was 100% at day 30 post-infusion. Although three patients experienced relapse during the mean follow-up period of 12.9 months, the overall durability of remission remained favorable, with an estimated 1-year PFS rate of 62.5%, indicating sustained clinical benefit in the majority of patients, comparable to findings reported by Maude et al.[26] In NHL, CR rates of 87.5% were observed at both day 90 and day 180. The 1-year PFS rate of 87.5% further supports the potential of CAR T cell therapy to induce durable responses in this patient population, surpassing outcomes reported in prior studies (Table S5).[6,27,28] CAR T cells persisted in the majority of patients, indicating sustained therapeutic activity. Although continued follow-up will be essential to assess long-term disease control and to identify factors predictive of durable response, the efficacy outcomes observed in this cohort are encouraging and reinforce the therapeutic promise of PoC CAR T cell therapy across hematologic malignancies.

Pre-treatment disease burden is widely recognized as a critical factor influencing the efficacy of CAR T cell therapy, with higher tumor load associated with lower response rates and poorer outcomes in both patients with ALL and patients with NHL.[6,26] Among the eight patients with NHL, one individual with bulky disease died due to disease progression, while CR was observed in the other seven patients without bulky disease. In the ALL group, two of the three patients who relapsed and subsequently died had pre-treatment blast percentages of 4% and 10%, respectively. In retrospect, both patients had a significant CD19-negative blast population at the time of infusion. In subsequent studies, we will use this as an exclusion criterion or shift similar patients to therapy with a dual antigen-targeting CAR T product (e.g., a CD19/CD22 CAR T cell product). The third relapsed patient was 40 years old and exhibited 0% blasts at baseline. Despite robust CAR T cell expansion and persistence post-infusion, leading to sustained B cell aplasia through day 90, the patient relapsed on day 128 with a CD19-negative clone. Notably, adult ALL is associated with a higher risk of relapse and poorer prognosis, which may have contributed to this outcome.[29]

CAR T cell expansion, persistence, and loss of B cell aplasia in patients

A higher peak of CAR T cell levels has been correlated with initial CR and negative minimal residual disease.[30] CAR T cell persistence is recognized as a key determinant of sustained therapeutic benefit in both patients with ALL and patients with NHL.[31,32] In our study, VinCART products exhibited robust expansion, peaking between days 10 and 14 and gradually declining thereafter, but still remaining detectable at the final follow-up on day 180, which is consistent with other reports.[26,33] The cells displayed a central memory phenotype during the first 2 weeks, then transitioned to an effector memory phenotype with cytotoxic functionality in both PB and BM. Central memory T cells are associated with durable CAR T cell persistence and antitumor efficacy,[34] which aligns with the high CR rates observed and suggests a sustained therapeutic response.

Early loss of B cell aplasia (within 6 months) is considered a risk factor for relapse.[9,35] Applying the threshold proposed by Sahai et al. (<50 B cells/μL),[36] we found that two out of five patients with ALL

Figure 5. Leukemic blasts and CAR T cells in relapsed patients

(A and B) Flow cytometry analysis of CD19 and CD3 expression in $CD45^{dim}$ SSC^{low} leukemic blasts and $CD45^{bright}$ SSC^{low} lymphocytes from patients BN03 (A) and BN07 (B) at three time points: prior to apheresis, day 30 (CR), and day 90 (relapse).

(C) Quantification of CAR T cell levels in BM and PB samples from patients BN03, BN07, and BN12 on days 30 and 90, based on flow cytometry.

(D) Quantitative PCR analysis of CAR VCN at the corresponding time points in relapsed patients.

(E) CD19+ B cell counts in BM and PB samples obtained before apheresis and on days 30 and 90 post-infusion. BM, bone marrow; CR, complete remission; PB, peripheral blood; PCR, polymerase chain reaction; VCN, vector copy number.

experienced loss of B cell aplasia, which corresponded with low to undetectable CAR T cell levels at days 60 and 180, respectively. In contrast, all seven patients with NHL maintained B cell aplasia throughout the observation period. Notably, both patients with ALL remained in CR, and the recovered B cells displayed differentiating phenotypes, suggesting reconstitution of normal B cell populations.

Economic considerations of VinCART therapy

The median cost of CAR T cell therapy in the US is US$620,500 and US$608,100 for ALL and DLBCL, respectively.[37,38] In comparison, the total cost per patient in our study was approximately US$120,000, including US$80,000 for CAR T cell production (inclusive of the lentiviral vector) and US$40,000 for clinical care-related costs. Our manufacturing cost of the CAR T cell product alone is comparable to the reported costs in India, where it is US$35,107, excluding the cost of the lentiviral vector, and to those reported by other PoC centers in Spain and Germany.[11,39] To further reduce the overall cost of CAR T cell therapy, in addition to PoC manufacturing, key strategies include optimizing CAR T cell production methods, lowering the cost of viral vectors, and minimizing the duration of hospital stays. Our excellent safety profile supports outpatient care of patients until the onset of fever, similar to standard practice in high-resource settings.[40]

Conclusion

PoC manufacturing of CAR T cells is feasible in resource-limited settings such as Vietnam, offering a cost-effective alternative to centralized production. CAR T cell therapies generated through PoC platforms have demonstrated safety and efficacy in the treatment of B cell ALL and NHL.

MATERIALS AND METHODS

Patients and study design

This phase I, single-center trial (NCT06027957) was conducted between August 2023 and June 2025 with approval from the National Ethics Committee (No. 64/CN-HDDD). The study followed the Declaration of Helsinki and ICH-GCP (International Council for Harmonisation - Good Clinical Practice) guidelines. CAR T cell therapy and related clinical costs were fully covered by the sponsor. All patients provided written informed consent. Patients with R/R B cell NHL and ALL were enrolled according to the inclusion and exclusion criteria (for details, see supplemental information).

Manufacturing CAR T cells using the CliniMACS Prodigy system

Mononuclear cells were collected using the Spectra Optia Apheresis system (Terumo BCT). CD19 CAR T cells were produced with the CliniMACS Prodigy system and Lentigen's CD19 CAR lentiviral vector (Miltenyi Biotec, Bergisch Gladbach, Germany) for 8–12 days. The lentiviral vector LTG1563 encoded an anti-CD19 single-chain variable fragment derived from FMC63, a CD8 linker, a TNFRSF19 transmembrane domain, a 4-1BB co-stimulatory domain, and a CD3-zeta chain intracellular signaling domain.[11,41] Quality control included assessments of $CD3^+$ T cells, transduction efficiency, viability, sterility, endotoxin, VCN, and cytotoxic potency (for details, see supplemental information).

Release criteria required 1–2 million ±20% CAR T cells/kg body weight, CD3+ T cells >70%, CAR T cells >10%, viability >70%, negative results for bacteria, fungi, and mycoplasma, endotoxin < 5 EU/mL, VCN < 5, and cytotoxicity against NALM6 cells.

Intervention

Patients underwent leukapheresis to obtain mononuclear cells for CAR T cell production. They received lymphodepleting conditioning with cyclophosphamide (NHL: 500 mg/m^2/day and ALL: 250 mg/m^2/day) and fludarabine (NHL: 30 mg/m^2/day and ALL: fludarabine 25 mg/m^2/day) from day −5 to day −3, followed by intravenous infusion of CAR T cells on day 0.

Toxicity monitoring

AEs and SAEs were recorded according to Common Terminology Criteria for Adverse Events (CTCAE) version 5.0,[42] with attribution graded in accordance with National Cancer Institute guidelines.[43] CRS and ICANS were evaluated using the American Society for Transplantation and Cellular Therapy (ASTCT) criteria.[44] IL-6 levels were measured every 24 h post-infusion and every 8 h following the onset of fever until resolution to guide tocilizumab use. Tocilizumab (8 mg/kg body weight/dose) was administered in response to fever accompanied by elevated IL-6 levels observed at two consecutive time points. Dexamethasone (10 mg per dose) was administered if CRS worsened following tocilizumab treatment.

Response assessment

ALL response was assessed via BM analysis of blast percentage using flow cytometry at baseline and on days 30, 90, and 180, in accordance with the 2022 National Comprehensive Cancer Network (NCCN) criteria.[45] NHL response was evaluated using positron emission tomography-computed tomography (PET-CT) at baseline and on days 90 and 180, according to the Lugano classification.[46]

Monitoring B cells, CAR T cells, and cytokines *in vivo*

PB samples were collected on days 3, 7, 10, 14, 21, 30, 60, 90, and 180 and monitored for CD19+ B cells, CAR T cells, plasma cytokines, and VCN according to the manufacturer's instructions (for details, see supplemental information).

Statistical analysis

Response outcomes and survival analyses were reported using frequency tables with absolute values, percentages, and 95% CIs. Categorical variables were compared using Fisher's exact test and continuous variables using nonparametric tests (the Wilcoxon rank-sum test for independent groups and the Wilcoxon signed-rank test for paired data). Exploratory comparisons between responders and non-responder/relapser for baseline and pharmacodynamic metrics used Fisher's exact test and the Wilcoxon rank-sum test. Survival was analyzed using Kaplan-Meier methods and compared with the

log-rank test. Statistical significance was set at $p < 0.05$. Analyses were performed using R vesion 3.5.2 and GraphPad Prism version 9.

DATA AND CODE AVAILABILITY

The data that support the findings of this study are available from the corresponding author upon reasonable request.

ACKNOWLEDGMENTS

We sincerely thank the doctors and nurses at the Department of Cell Therapy and Regenerative Medicine, Vinmec Healthcare System, for their exceptional patient care. We are grateful to colleagues at the HiTech Center and Laboratory Department for their support in biobanking, quality control, and labor management. Special thanks to Dang Van Duc (German Rheumatism Research Center, Germany) for his support with flow cytometric analysis support, and to Jacek Toporski (Karolinska University Hospital, Sweden), Fabio Ciceri and Jacopo Peccatori (Vita-Salute San Raffaele University, Italy), Rupert Handgretinger (University Hospital of Tübingen, Germany), and Dr. Michael Aigner (University Hospital of Erlangen, Germany) for their generous contributions to clinical and cell manufacturing training. We also acknowledge Miltenyi Biotec for technical training and lentiviral vector support. Most importantly, we thank the patients and their families for their trust and participation.

This investigation was supported by the Vingroup Research Grant (project no: ISC.19.26). The funding body played no role in the design of the study, data collection, analysis, or interpretation, or in writing the manuscript.

The trial was conducted with approval from the National Ethical Committee (no. 64/CN-HDDD) and registered on ClinicalTrials.gov (NCT06027957). The study followed the Declaration of Helsinki and ICH-GCP guidelines. CAR T cell therapy and related clinical costs were fully covered by the sponsor. All patients provided written informed consent.

During the preparation of this work, we used Microsoft Copilot to check grammar and improve clarity. After using this tool, we reviewed and edited the content as needed and take full responsibility for the content of the published article.

AUTHOR CONTRIBUTIONS

L.T.N., V.T.H., Q.K.B., D.D.N., and V.H.T.P. contributed to the study conception and design. V.T.H., L.T.M.D., T.T.K.P., H.-N.D., P.T.P., H.T.M., T.H.P., and T.M.T.N. were responsible for apheresis, CAR T cell manufacturing and QC, and follow-up analyses. L.T.N., D.D.N., Q.K.B., V.B.L., N.L.P., N.Q.N., M.L.H., Q.L.P., D.Q.T.N., L.M., and Q.N.N. provided patient care. D.D.N., T.H.P., H.-P.N., V.T.H., L.T.M.D., T.T.K.P., and H.-N.D. performed data collection and analysis. L.T.N., V.T.H., D.D.N., H.-P.N., T.H.P., and M.L.H. drafted the manuscript. All authors critically reviewed the manuscript and approved the final version.

DECLARATION OF INTERESTS

The authors declare no competing interests.

SUPPLEMENTAL INFORMATION

Supplemental information can be found online at https://doi.org/10.1016/j.omton.2026.201156.

REFERENCES

1. Mafra, A., Laversanne, M., Gospodarowicz, M., Klinger, P., De Paula Silva, N., Piñeros, M., Steliarova-Foucher, E., Bray, F., and Znaor, A. (2022). Global patterns of non-Hodgkin lymphoma in 2020. Int. J. Cancer *151*, 1474–1481. https://doi.org/10.1002/ijc.34163.

2. Wang, W., Zhang, D., Liang, Q., Liu, X., Shi, J., and Zhou, F. (2024). Global burden, risk factor analysis, and prediction study of leukaemia from 1990 to 2030. J. Glob. Health *14*, 04150. https://doi.org/10.7189/jogh.14.04150.

3. Spyridonidis, A., Labopin, M., Schmid, C., Volin, L., Yakoub-Agha, I., Stadler, M., Milpied, N., Socie, G., Browne, P., Lenhoff, S., et al. (2012). Outcomes and prognostic factors of adults with acute lymphoblastic leukemia who relapse after allogeneic hematopoietic cell transplantation. An analysis on behalf of the Acute Leukemia Working Party of EBMT. Leukemia *26*, 1211–1217. https://doi.org/10.1038/leu.2011.351.

4. Sehn, L.H., and Salles, G. (2021). Diffuse Large B-Cell Lymphoma. N. Engl. J. Med. *384*, 842–858. https://doi.org/10.1056/NEJMra2027612.

5. Crump, M., Neelapu, S.S., Farooq, U., Van Den Neste, E., Kuruvilla, J., Westin, J., Link, B.K., Hay, A., Cerhan, J.R., Zhu, L., et al. (2017). Outcomes in refractory diffuse large B-cell lymphoma: results from the international SCHOLAR-1 study. Blood *130*, 1800–1808. https://doi.org/10.1182/blood-2017-03-769620.

6. Locke, F.L., Ghobadi, A., Jacobson, C.A., Miklos, D.B., Lekakis, L.J., Oluwole, O.O., Lin, Y., Braunschweig, I., Hill, B.T., Timmerman, J.M., et al. (2019). Long-term safety and activity of axicabtagene ciloleucel in refractory large B-cell lymphoma (ZUMA-1): a single-arm, multicentre, phase 1–2 trial. Lancet *20*, 31–42. https://doi.org/10.1016/S1470-2045(18)30864-7.

7. Meng, J., Wu, X., Sun, Z., Xun, R., Liu, M., Hu, R., and Huang, J. (2021). Efficacy and Safety of CAR-T Cell Products Axicabtagene Ciloleucel, Tisagenlecleucel, and Lisocabtagene Maraleucel for the Treatment of Hematologic Malignancies: A Systematic Review and Meta-Analysis. Front. Oncol. *11*, 698607. https://doi.org/10.3389/fonc.2021.698607.

8. Goyco Vera, D., Waghela, H., Nuh, M., Pan, J., and Lulla, P. (2024). Approved CAR-T therapies have reproducible efficacy and safety in clinical practice. Hum. Vaccin. Immunother. *20*, 2378543. https://doi.org/10.1080/21645515.2024.2378543.

9. Laetsch, T.W., Maude, S.L., Rives, S., Hiramatsu, H., Bittencourt, H., Bader, P., Baruchel, A., Boyer, M., De Moerloose, B., Qayed, M., et al. (2023). Three-Year Update of Tisagenlecleucel in Pediatric and Young Adult Patients With Relapsed/Refractory Acute Lymphoblastic Leukemia in the ELIANA Trial. J. Clin. Oncol. *41*, 1664–1669. https://doi.org/10.1200/jco.22.00642.

10. Abdo, L., Batista-Silva, L.R., and Bonamino, M.H. (2025). Cost-effective strategies for CAR-T cell therapy manufacturing. Mol. Ther. Oncol. *33*, 200980. https://doi.org/10.1016/j.omton.2025.200980.

11. Palani, H.K., Arunachalam, A.K., Kulkarni, U., Yasar, M., Venkatraman, A., Palanikumar, S., Radhakrishnan, R.N., Solomon, M., Rajasekaran, A., Bankar, A., et al. (2025). Safety, efficacy and total cost of point-of-care manufactured anti-CD19 CAR-T cell therapy in India: VELCART trial. Mol. Ther. Oncol. *33*, 200977. https://doi.org/10.1016/j.omton.2025.200977.

12. Zhang, W., Jordan, K.R., Schulte, B., and Purev, E. (2018). Characterization of clinical grade CD19 chimeric antigen receptor T cells produced using automated CliniMACS Prodigy system. Drug Des. Devel. Ther. *12*, 3343–3356. https://doi.org/10.2147/dddt.S175113.

13. Blache, U., Popp, G., Dünkel, A., Koehl, U., and Fricke, S. (2022). Potential solutions for manufacture of CAR T cells in cancer immunotherapy. Nat. Commun. *13*, 5225. https://doi.org/10.1038/s41467-022-32866-0.

14. Reddy, O.L., Stroncek, D.F., and Panch, S.R. (2020). Improving CAR T cell therapy by optimizing critical quality attributes. Semin. Hematol. *57*, 33–38. https://doi.org/10.1053/j.seminhematol.2020.07.005.

15. De Angelis, B., D'Amore, M.L., Lecot, P., Neininger, K., Lorrain, M., Gambotti, L., Dreuillet, C., Courcault, E., Chatterjee, S., Delgado, J., et al. (2025). European survey on CAR T-Cell analytical methods from apheresis to post-infusion immunomonitoring. Front. Immunol. *16*, 1567582. https://doi.org/10.3389/fimmu.2025.1567582.

16. (FDA), U.S.F.a.D.A. (2022). Considerations for the Development of Chimeric Antigen Receptor (CAR) T Cell Products: Guidance for Industry ((FDA), U.S.F.a.D.A).

17. Chen, A., Velickovic, Z., and Rasko, J. (2020). Vector copy number quality control testing for CAR T-cells: critical validation parameters. Cytotherapy *22*, S142. https://doi.org/10.1016/j.jcyt.2020.03.283.

18. Worel, N., Holbro, A., Vrielink, H., Ootjers, C., Le Poole, K., Beer-Wekking, I., Rintala, T., Lozano, M., and Bonig, H. (2023). A guide to the collection of T-cells by apheresis for ATMP manufacturing-recommendations of the GoCART coalition apheresis working group. Bone Marrow Transplant. *58*, 742–748. https://doi.org/10.1038/s41409-023-01957-x.

19. Delgado, J., Roddie, C., and Schmitt, M. (2022). Point-of-Care Production of CAR-T Cells. In The EBMT/EHA CAR-T Cell Handbook, N. Kröger, J. Gribben, C.

Chabannon, I. Yakoub-Agha, and H. Einsele, eds. (Springer International Publishing), pp. 45–49. https://doi.org/10.1007/978-3-030-94353-0_8.

20. Schuster, S.J., Bishop, M.R., Tam, C.S., Waller, E.K., Borchmann, P., McGuirk, J.P., Jäger, U., Jaglowski, S., Andreadis, C., Westin, J.R., et al. (2019). Tisagenlecleucel in Adult Relapsed or Refractory Diffuse Large B-Cell Lymphoma. N. Engl. J. Med. *380*, 45–56. https://doi.org/10.1056/NEJMoa1804980.

21. Abramson, J.S., Palomba, M.L., Gordon, L.I., Lunning, M.A., Wang, M., Arnason, J., Mehta, A., Purev, E., Maloney, D.G., Andreadis, C., et al. (2020). Lisocabtagene maraleucel for patients with relapsed or refractory large B-cell lymphomas (TRANSCEND NHL 001): a multicentre seamless design study. Lancet *396*, 839–852. https://doi.org/10.1016/s0140-6736(20)31366-0.

22. Lee, D.W., Gardner, R., Porter, D.L., Louis, C.U., Ahmed, N., Jensen, M., Grupp, S.A., and Mackall, C.L. (2014). Current concepts in the diagnosis and management of cytokine release syndrome. Blood *124*, 188–195. https://doi.org/10.1182/blood-2014-05-552729.

23. Hayden, P.J., Roddie, C., Bader, P., Basak, G.W., Bonig, H., Bonini, C., Chabannon, C., Ciceri, F., Corbacioglu, S., Ellard, R., et al. (2022). Management of adults and children receiving CAR T-cell therapy: 2021 best practice recommendations of the European Society for Blood and Marrow Transplantation (EBMT) and the Joint Accreditation Committee of ISCT and EBMT (JACIE) and the European Haematology Association (EHA). Ann. Oncol. *33*, 259–275. https://doi.org/10.1016/j.annonc.2021.12.003.

24. Kadauke, S., Myers, R.M., Li, Y., Aplenc, R., Baniewicz, D., Barrett, D.M., Barz Leahy, A., Callahan, C., Dolan, J.G., Fitzgerald, J.C., et al. (2021). Risk-Adapted Preemptive Tocilizumab to Prevent Severe Cytokine Release Syndrome After CTL019 for Pediatric B-Cell Acute Lymphoblastic Leukemia: A Prospective Clinical Trial. J. Clin. Oncol. *39*, 920–930. https://doi.org/10.1200/jco.20.02477.

25. Luan, C., Wang, H., Zhou, J., Long, Z., Chen, X., Chen, X., Ni, J., Huang, Z., Xia, R., and Ge, J. (2023). Improved efficacy with early tocilizumab in the prophylaxis and treatment of cytokine release syndrome of chimeric antigen receptor T cell (CAR-T) therapy for diffuse large B-cell lymphoma than acute lymphoblastic leukemia. Biomed. Technol. *3*, 59–65. https://doi.org/10.1016/j.bmt.2023.01.002.

26. Maude, S.L., Laetsch, T.W., Buechner, J., Rives, S., Boyer, M., Bittencourt, H., Bader, P., Verneris, M.R., Stefanski, H.E., Myers, G.D., et al. (2018). Tisagenlecleucel in Children and Young Adults with B-Cell Lymphoblastic Leukemia. N. Engl. J. Med. *378*, 439–448. https://doi.org/10.1056/NEJMoa1709866.

27. Schuster, S.J., Tam, C.S., Borchmann, P., Worel, N., McGuirk, J.P., Holte, H., Waller, E.K., Jaglowski, S., Bishop, M.R., Damon, L.E., et al. (2021). Long-term clinical outcomes of tisagenlecleucel in patients with relapsed or refractory aggressive B-cell lymphomas (JULIET): a multicentre, open-label, single-arm, phase 2 study. Lancet Oncol. *22*, 1403–1415. https://doi.org/10.1016/S1470-2045(21)00375-2.

28. Abramson, J.S., Palomba, M.L., Gordon, L.I., Lunning, M.A., Wang, M., Arnason, J.E., Purev, E., Maloney, D.G., Andreadis, C., Sehgal, A.R., et al. (2021). Two-Year Follow-up of Transcend NHL 001, a Multicenter Phase 1 Study of Lisocabtagene Maraleucel (liso-cel) in Relapsed or Refractory (R/R) Large B-Cell Lymphomas (LBCL). Blood *138*, 2840. https://doi.org/10.1182/blood-2021-148948.

29. Wudhikarn, K., Flynn, J.R., Rivière, I., Gönen, M., Wang, X., Senechal, B., Curran, K.J., Roshal, M., Maslak, P.G., Geyer, M.B., et al. (2021). Interventions and outcomes of adult patients with B-ALL progressing after CD19 chimeric antigen receptor T-cell therapy. Blood *138*, 531–543. https://doi.org/10.1182/blood.2020009515.

30. Park, J.H., Rivière, I., Gonen, M., Wang, X., Sénéchal, B., Curran, K.J., Sauter, C., Wang, Y., Santomasso, B., Mead, E., et al. (2018). Long-Term Follow-up of CD19 CAR Therapy in Acute Lymphoblastic Leukemia. N. Engl. J. Med. *378*, 449–459. https://doi.org/10.1056/NEJMoa1709919.

31. Mueller, K.T., Maude, S.L., Porter, D.L., Frey, N., Wood, P., Han, X., Waldron, E., Chakraborty, A., Awasthi, R., Levine, B.L., et al. (2017). Cellular kinetics of CTL019 in relapsed/refractory B-cell acute lymphoblastic leukemia and chronic lymphocytic leukemia. Blood *130*, 2317–2325. https://doi.org/10.1182/blood-2017-06-786129.

32. Wittibschlager, V., Bacher, U., Seipel, K., Porret, N., Wiedemann, G., Haslebacher, C., Hoffmann, M., Daskalakis, M., Akhoundova, D., and Pabst, T. (2023). CAR T-Cell Persistence Correlates with Improved Outcome in Patients with B-Cell Lymphoma. Int. J. Mol. Sci. *24*, 5688. https://doi.org/10.3390/ijms24065688.

33. Finney, O.C., Brakke, H.M., Rawlings-Rhea, S., Hicks, R., Doolittle, D., Lopez, M., Futrell, R.B., Orentas, R.J., Li, D., Gardner, R.A., and Jensen, M.C. (2019). CD19 CAR T cell product and disease attributes predict leukemia remission durability. J. Clin. Investig. *129*, 2123–2132. https://doi.org/10.1172/JCI125423.

34. Tao, Z., Chyra, Z., Kotulová, J., Celichowski, P., Mihályová, J., Charvátová, S., and Hájek, R. (2024). Impact of T cell characteristics on CAR-T cell therapy in hematological malignancies. Blood Cancer J. *14*, 213. https://doi.org/10.1038/s41408-024-01193-6.

35. Ortiz-Maldonado, V., Rives, S., Espanol-Rego, M., Alonso-Saladrigues, A., Montoro, M., Magnano, L., Gine, E., Pascal, M., Diaz-Beya, M., Castella, M., et al. (2021). Factors associated with the clinical outcome of patients with relapsed/refractory CD19(+) acute lymphoblastic leukemia treated with ARI-0001 CART19-cell therapy. J. Immunother. Cancer *9*, e003644. https://doi.org/10.1136/jitc-2021-003644.

36. Sahai, I., Borgman, P., Yates, B., Rosenzweig, S., Rampertaap, S., Rankin, A.W., and Shah, N.N. (2024). Incidence of preexisting B-cell aplasia in B-ALL: implications for post-CAR T-cell monitoring. Blood Adv. *8*, 6329–6333. https://doi.org/10.1182/bloodadvances.2024014641.

37. Hoover, A., Reimche, P., Watson, D., Tanner, L., Gilchrist, L., Finch, M., Messinger, Y.H., and Turcotte, L.M. (2024). Healthcare cost and utilization for chimeric antigen receptor (CAR) T-cell therapy in the treatment of pediatric acute lymphoblastic leukemia: A commercial insurance claims database analysis. Cancer Rep. *7*, e1980. https://doi.org/10.1002/cnr2.1980.

38. Di, M., Potnis, K.C., Long, J.B., Isufi, I., Foss, F., Seropian, S., Gross, C.P., and Huntington, S.F. (2024). Costs of care during chimeric antigen receptor T-cell therapy in relapsed or refractory B-cell lymphomas. JNCI Cancer Spectr. *8*, pkae059. https://doi.org/10.1093/jncics/pkae059.

39. Cliff, E.R.S., Kelkar, A.H., Russler-Germain, D.A., Tessema, F.A., Raymakers, A.J.N., Feldman, W.B., and Kesselheim, A.S. (2023). High Cost of Chimeric Antigen Receptor T-Cells: Challenges and Solutions. Am. Soc. Clin. Oncol. Educ. Book. *43*, e397912. https://doi.org/10.1200/EDBK_397912.

40. Hansen, D.K., Liu, Y.H., Ranjan, S., Bhandari, H., Potluri, R., McFarland, L., De Braganca, K.C., and Huo, S. (2023). The Impact of Outpatient versus Inpatient Administration of CAR-T Therapies on Clinical, Economic, and Humanistic Outcomes in Patients with Hematological Cancer: A Systematic Literature Review. Cancers (Basel) *15*, 5746. https://doi.org/10.3390/cancers15245746.

41. Ghobadi, A., Caimi, P.F., Reese, J.S., Goparaju, K., di Trani, M., Ritchey, J., Jackson, Z., Tomlinson, B., Schiavone, J.M., Kleinsorge-Block, S., et al. (2025). Treatment of non-Hodgkin lymphoma with point-of-care manufactured CAR T cells: a dual institution, phase 1 trial. eClinicalMedicine *81*, 103138. https://doi.org/10.1016/j.eclinm.2025.103138.

42. SERVICES, U.S.D.O.H.A.H (2017). Common Terminology Criteria for Adverse Events (CTCAE) (U.S. Department of health and human services) Version 5.0. .

43. Institute, N.C. (2013). NCI Guidelines for Investigators: Adverse Event Reporting Requirements for DCTD (CTEP and CIP) and DCP INDs and IDEs (National Cancer Institute).

44. Lee, D.W., Santomasso, B.D., Locke, F.L., Ghobadi, A., Turtle, C.J., Brudno, J.N., Maus, M.V., Park, J.H., Mead, E., Pavletic, S., et al. (2019). ASTCT Consensus Grading for Cytokine Release Syndrome and Neurologic Toxicity Associated with Immune Effector Cells. Biol. Blood Marrow Transplant. *25*, 625–638. https://doi.org/10.1016/j.bbmt.2018.12.758.

45. Brown, P.A., Shah, B., Advani, A., Aoun, P., Boyer, M.W., Burke, P.W., DeAngelo, D.J., Dinner, S., Fathi, A.T., Gauthier, J., et al. (2021). Acute Lymphoblastic Leukemia, Version 2.2021, NCCN Clinical Practice Guidelines in Oncology. J. Natl. Compr. Canc. Netw. *19*, 1079–1109. https://doi.org/10.6004/jnccn.2021.0042.

46. Cheson, B.D., Fisher, R.I., Barrington, S.F., Cavalli, F., Schwartz, L.H., Zucca, E., Lister, T.A., Alliance Australasian Leukaemia and Lymphoma Group, Eastern Cooperative Oncology Group, European Mantle Cell Lymphoma Consortium, et al. (2014). Recommendations for initial evaluation, staging, and response assessment of Hodgkin and non-Hodgkin lymphoma: the Lugano classification. J. Clin. Oncol. *32*, 3059–3068. https://doi.org/10.1200/JCO.2013.54.8800.

OMTON, Volume *34*

Supplemental information

Outcomes of point-of-care manufactured CAR T cell therapy for B cell acute lymphoblastic leukemia and non-Hodgkin lymphoma in Vietnam

Liem Thanh Nguyen, Duy D. Nguyen, Quoc Khanh Bach, Lan T.M. Dao, Trang Thi Kieu Phan, Hoang - Phuong Nguyen, Hong-Nhung Dao, Trang H. Pham, Phuong T. Pham, Hien T. Mai, Viet Huong T. Pham, Thanh Mai T. Nguyen, Van Binh Le, Nam Lam Phung, Ngoc Quang Nguyen, Michelle L. Hermiston, Quynh Lan Phan, Do Quang Trung Nguyen, Lan Mai, Quoc Nhat Nguyen, and Van T. Hoang

Supplemental information

Methods

Eligibility

Inclusion criteria

- Both sexes, aged 1 to 60 years, diagnosed with relapsed or refractory (R/R) B-cell non-Hodgkin lymphoma (B-NHL) or B-cell acute lymphoblastic leukemia (B-ALL).
- B-ALL refractory to ≥2 cycles of chemotherapy or relapsed after chemotherapy or hematopoietic stem cell transplantation (HSCT); or B-NHL refractory to ≥2 lines of chemotherapy or relapsed after chemotherapy or HSCT.
- CD19 positivity confirmed by immunohistochemistry or flow cytometry.
- Absolute neutrophil count (ANC) ≥ 1.0 G/L without filgrastim support, absolute lymphocyte count ≥ 0.1 G/L, platelet count ≥ 75 G/L, and hemoglobin ≥ 8.0 g/dL.
- Renal function: serum creatinine ≤ 1.5 × upper limit of normal or estimated glomerular filtration rate ≥ 60 mL/min/1.73 m^2.
- Hepatic function: AST and ALT ≤ 5 × upper limit of normal; total bilirubin ≤ 2.0 mg/dL.
- Pulmonary status: no chronic pulmonary diseases requiring continuous medication and no respiratory insufficiency (oxygen saturation SpO_2 > 92% on room air).
- Cardiac function: no arrhythmias, intracardiac or vascular thrombosis, or heart failure, with left ventricular ejection fraction (LVEF) ≥ 45%.
- Willingness and ability to provide written informed consent to participate in the study.
- Female patients of childbearing potential agreed to use effective contraception during the study period.

Exclusion criteria

- Central nervous system involvement at the time of screening; history of autoimmune neurological disorders, neuromyelitis optica, or clinically significant CNS diseases.
- Diagnosed primary immunodeficiency.
- History of veno-occlusive disease.
- Acute medical conditions requiring immediate treatment; active, progressive, or chronic graft-versus-host disease; or active infectious disease.
- History of or concurrent malignancy other than B-cell lymphoma or B-ALL.
- Active hemolytic anemia.
- Receiving immunosuppressive therapy, except for ≤ 30 mg prednisolone or equivalent at the time of CAR-T-cell infusion.
- Critically ill patients or those at risk of premature death, including acute liver failure requiring dialysis, heart failure requiring vasopressors, systemic infection unresponsive to antibiotics, or ECOG performance status ≥ 3 at screening.
- Other severe concomitant diseases (e.g., uncontrolled hypertension or NYHA class III–IV heart failure) or unstable angina within 3 months prior to screening.
- Intolerance to excipients used in cellular products.
- Pregnant or breastfeeding women, or women planning pregnancy during the study period.
- Participation in another clinical trial at the time of screening.

Manufacturing CAR-T cells using the CliniMACS Prodigy system

Mononuclear cell collection was performed using the Spectra Optia Apheresis system with a MNC Spectra Optia Kit (Terumo BCT). Clinical-grade CD19-targeted CAR T-cells were manufactured using the automated CliniMACS Prodigy system with the TCT program and TS520 tubing set (Miltenyi Biotec, Germany). CD4+ and CD8+ T cells were isolated using CliniMACS CD4 and CD8 Reagents (Miltenyi Biotec, Germany) and cultured in Miltenyi's

TexMACS GMP Medium supplemented with 3% human AB serum (HiMedia, India) and recombinant human IL-7 and IL-15 (Miltenyi Biotec, Germany). Cells were activated with MACS® GMP T Cell TransAct™ for anti-CD3/CD28 co-stimulation overnight before transduction with Lentigen's CD19 CAR lentiviral vector (Miltenyi Biotec, Germany). Transduced T cells were expanded in TexMACS GMP Medium supplemented with 3% human AB serum (HiMedia, India) and recombinant human IL-7 and IL-15 for 8-9 days. On the day of infusion, CAR T-cells were harvested in 0.9% NaCl (Bbraun, Vietnam) supplemented with 0.5% human serum albumin (Grifols Biologicals Inc., USA).

Quality controls of CAR-T cells

Flow cytometry

CAR T-cells were stained with CD45 VioBlue, CD4 VioGreen, CD3 FITC, CD16/CD56 PE, CD19 PE-Vio 770, CD14 APC, CD8 APC-Vio 770 antibodies, and 7-AAD, and analyzed using a MACS Quant 10 (Miltenyi Biotec, Germany) following the manufacturer's instructions to assess immunophenotype and viability. Transduction efficiency was determined using CD45 VioBlue, CD4 VioGreen, CD3 FITC, CAR-DR PE, CD14 APC, CD8 APC-Vio 770, and 7-AAD (Miltenyi Biotec, Germany). Flow cytometry data were analyzed using MACSQuant Analyzers software.

Sterility

Sterility testing for bacteria and fungi was performed using the BacT/Alert3D microbial detection system (bioMérieux, Durham, North Carolina) at the CAP-accredited Vinmec laboratory department, Vinmec Times City Hospital. Mycoplasma contamination was assessed using the MycoAlert Mycoplasma Detection Kit (Lonza, Switzerland), and endotoxin levels were measured with the EndoSafe-PTS Kit (Charles River Laboratories, USA) according to the manufacturer's instructions.

Vector copy number was quantified using TaqMan real-time PCR with the MACS COPYcheck Kit (Miltenyi Biotec, Germany), which measures the lentiviral gag gene and the human reference gene PTBP2, following the manufacturer's instructions.

Potency assay

CAR T-cells were co-cultured with the CD19+ NALM6 cell line at an effector-to-target cell ratio of 5:1, stained with CD3 FITC, CD19 PE-Vio 770, and 7-AAD, and analyzed using a MACSQuant 10 (Miltenyi Biotec, Germany). The number of CD19+ cells was quantified and compared to samples containing only target cells. Additionally, the supernatant from the co-culture was collected, and concentrations of GM-CSF, Granzyme B, IFN-γ, IL-2, IL-4, IL-6, IL-10, IL-17A, IL-21, MCP-1 (CCL2), Perforin, and TNF-α were measured using the human MACSPlex Cytotoxic T/NK Cell Kit (Miltenyi Biotec, Germany) according to the manufacturer's instructions.

Monitoring B-cells, CAR T-cells, and cytokines in vivo

Peripheral blood samples from treated patients were collected on days 3, 7, 10, 14, 21, 30, 60, 90, and 180. Peripheral blood mononuclear cells (PBMCs) were isolated by density gradient centrifugation using Ficoll-Paque™ PREMIUM density gradient media (Cytiva, Sweden). B-cell quantification was performed by flow cytometry using the following antibodies: CD45 Vioblue, CD4 Viogreen, CD3 FITC, CD16/CD56 PE, CD19 PE-Vio® 770, CD14 APC, CD8 APC-Vio® 770, and 7-AAD, analyzed on a MACSQuant 10 (Miltenyi Biotec, Germany). CAR T-cell numbers and phenotypes were assessed by staining with CD45RA Vioblue, CD223 Vioblue, CD4 Viogreen, CD3 FITC, CD14-PerCP-Vio700, CD15-PerCP-Vio700, CAR-DR PE, CD62L PE-Vio® 770, CD279 PE-Vio® 770, CD45RO APC, CD366 APC, CD8 APC-Vio® 770, and 7-AAD (Miltenyi Biotec, Germany). Flow cytometry data were analyzed using MACSQuant® Analyzer software.

Plasma levels of GM-CSF, IFN-α, IFN-γ, IL-2, IL-4, IL-5, IL-6, IL-9, IL-10, IL-12p70, IL-17A, and TNF-α were measured using the MACSPlex Cytokine 12 Kit (Miltenyi Biotec, Germany) according to the manufacturer's instructions.

CAR T-cell copy number was quantified by TaqMan real-time PCR using the MACS® COPYcheck Kit (Miltenyi Biotec, Germany) following the manufacturer's instructions.

Statistical analysis

Response outcomes and survival analysis were reported using frequency tables with absolute values, percentages, and 95% CIs. Categorical variables were compared using Fisher's exact test, and continuous variables using nonparametric tests (the Wilcoxon rank-sum test for independent groups and the Wilcoxon signed-rank test for paired data). Exploratory responder versus non-responder/relapser comparisons of baseline and pharmacodynamic metrics used Fisher's exact and Wilcoxon rank-sum tests. Survival was analyzed via Kaplan-Meier methods and compared using the Log-rank test. Statistical significance was set at $p < 0.05$. Analyses were performed in R vesion 3.5.2 and GraphPad Prism version 9.

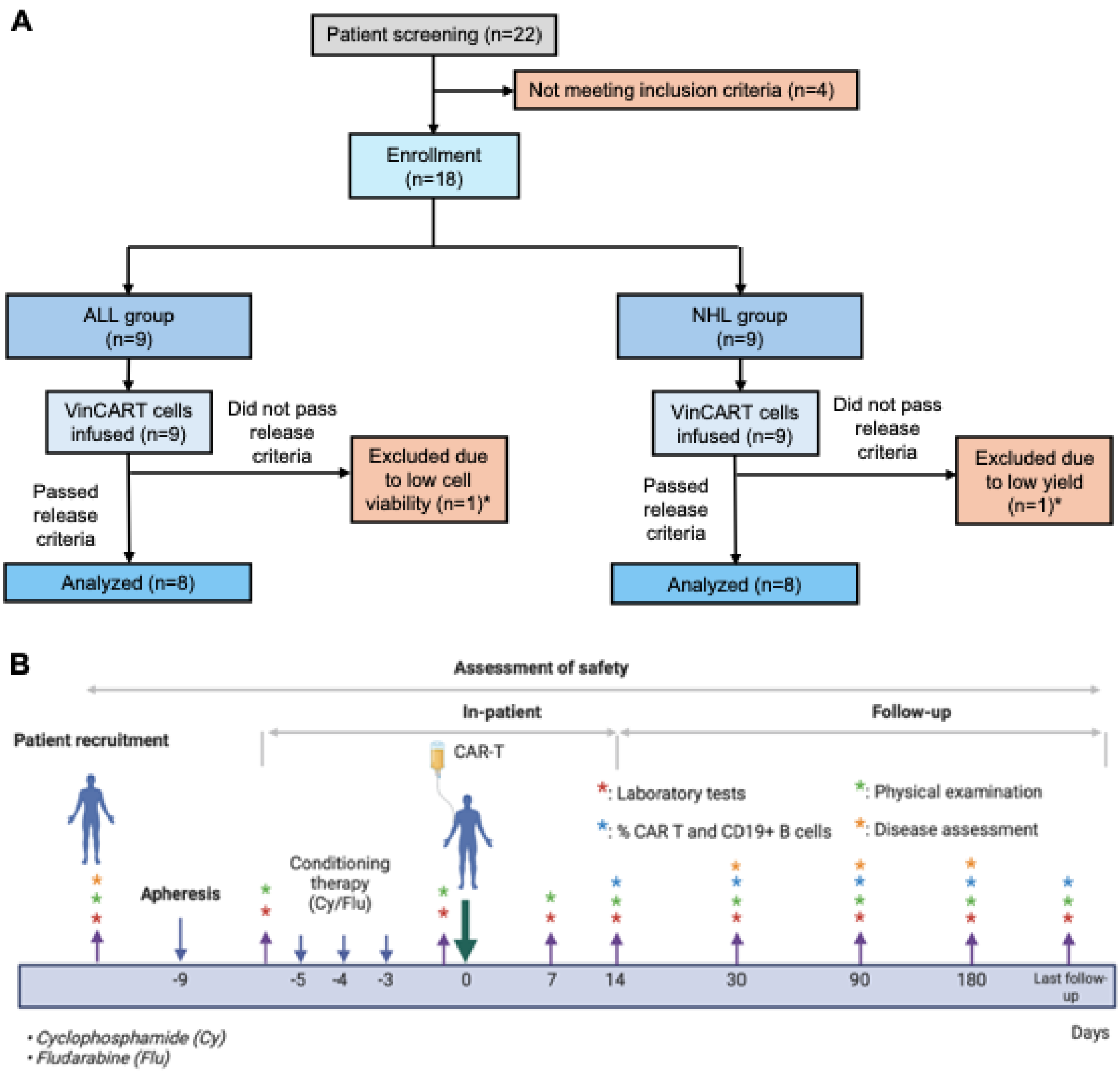

Figure S1. Patients and study design. A. Consort diagram of patients included in the VinCART trial. *Excluded from the evaluable cohort due to not meeting protocol eligibility criteria but still infused with CD19 CAR-T. **B.** Study timeline for CAR T-cell therapy administration and follow-up. Patients received apheresis on day -9 (or day -13 in the first two patients), lymphodepleting conditioning therapy with cyclophosphamide (Cy) and fludarabine (Flu) from days -5 to -3, CAR-T infusion on day 0, and scheduled follow-up visits for safety, laboratory testing, and outcome assessments through day 180 and last visit.

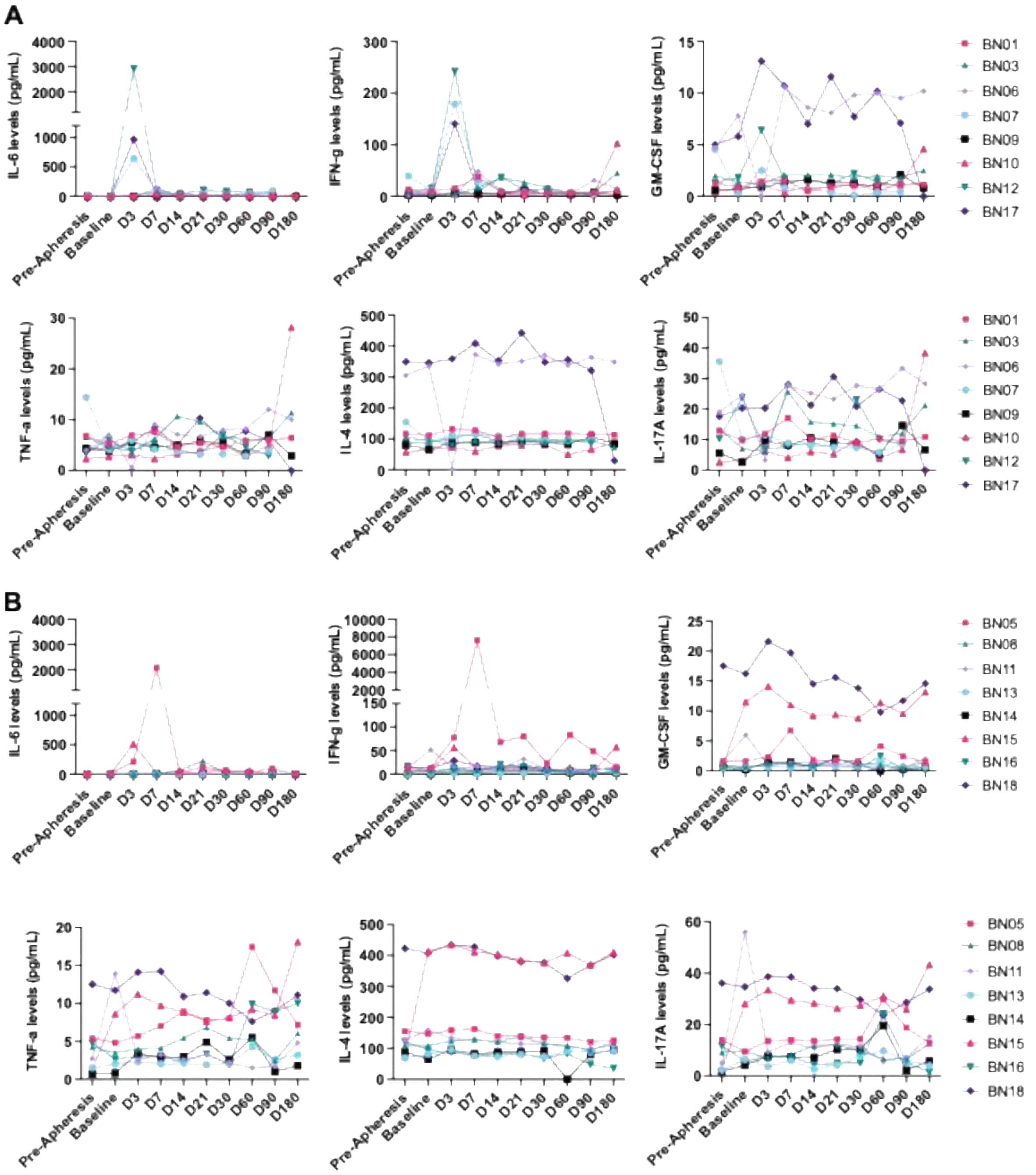

Figure S2: Cytokine levels in plasma of ALL (A) and NHL (B) patients treated with CAR T-cells. IL-2, IL-9, IL-10 levels remained below the assay limit of detection.

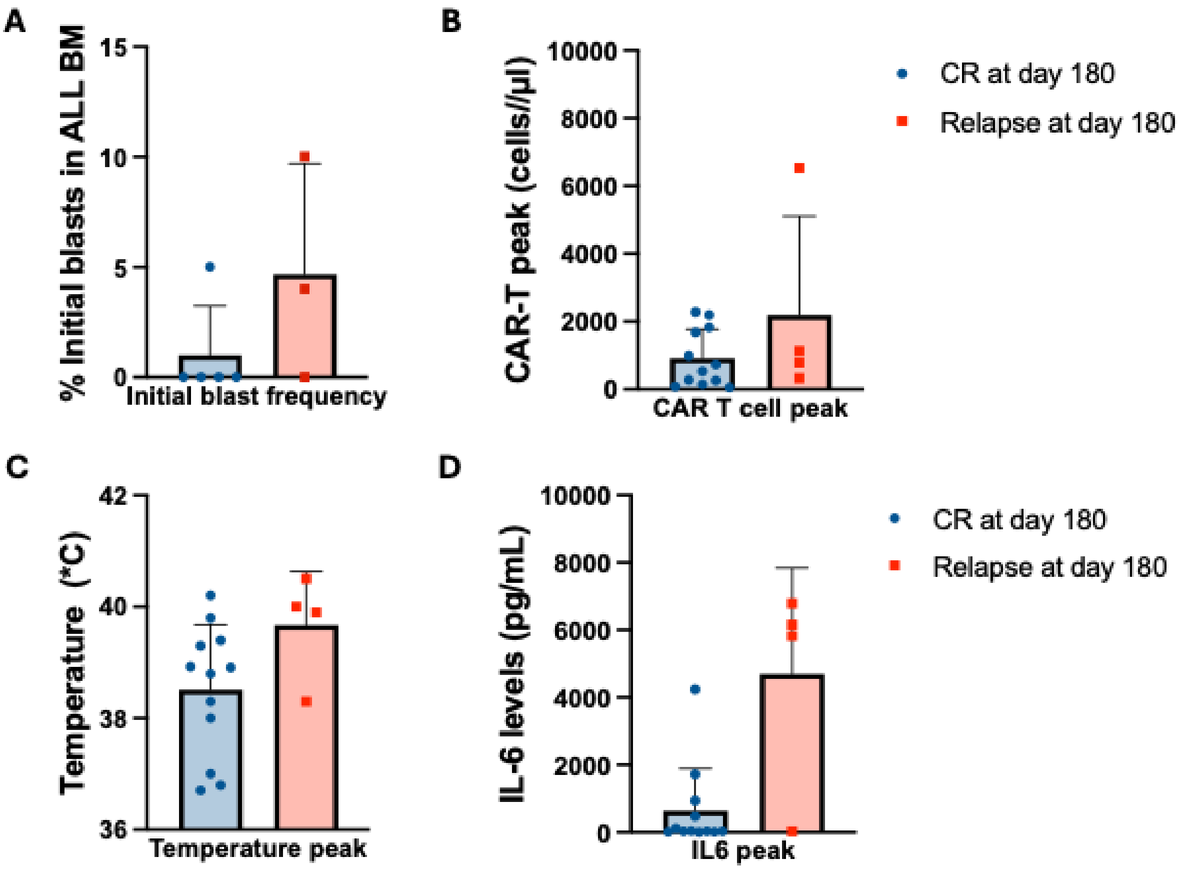

Figure S3. Correlations with clinical outcome. (A) Frequency of leukemic blasts in the BM of ALL patients at baseline, comparing those who achieved CR at day 180 with those who experienced disease refractoriness or relapse (R/R). **(B)** CAR T-cell levels measured by flow cytometry in the PB of the CR and R/R groups. **(C–D)** Peak body temperature (C) and IL-6 concentration (D) recorded following CAR T-cell infusion in both patient groups. Abbreviations: bone marrow (BM), complete remission (CR), peripheral blood (PB), and refractoriness/relapse (R/R).

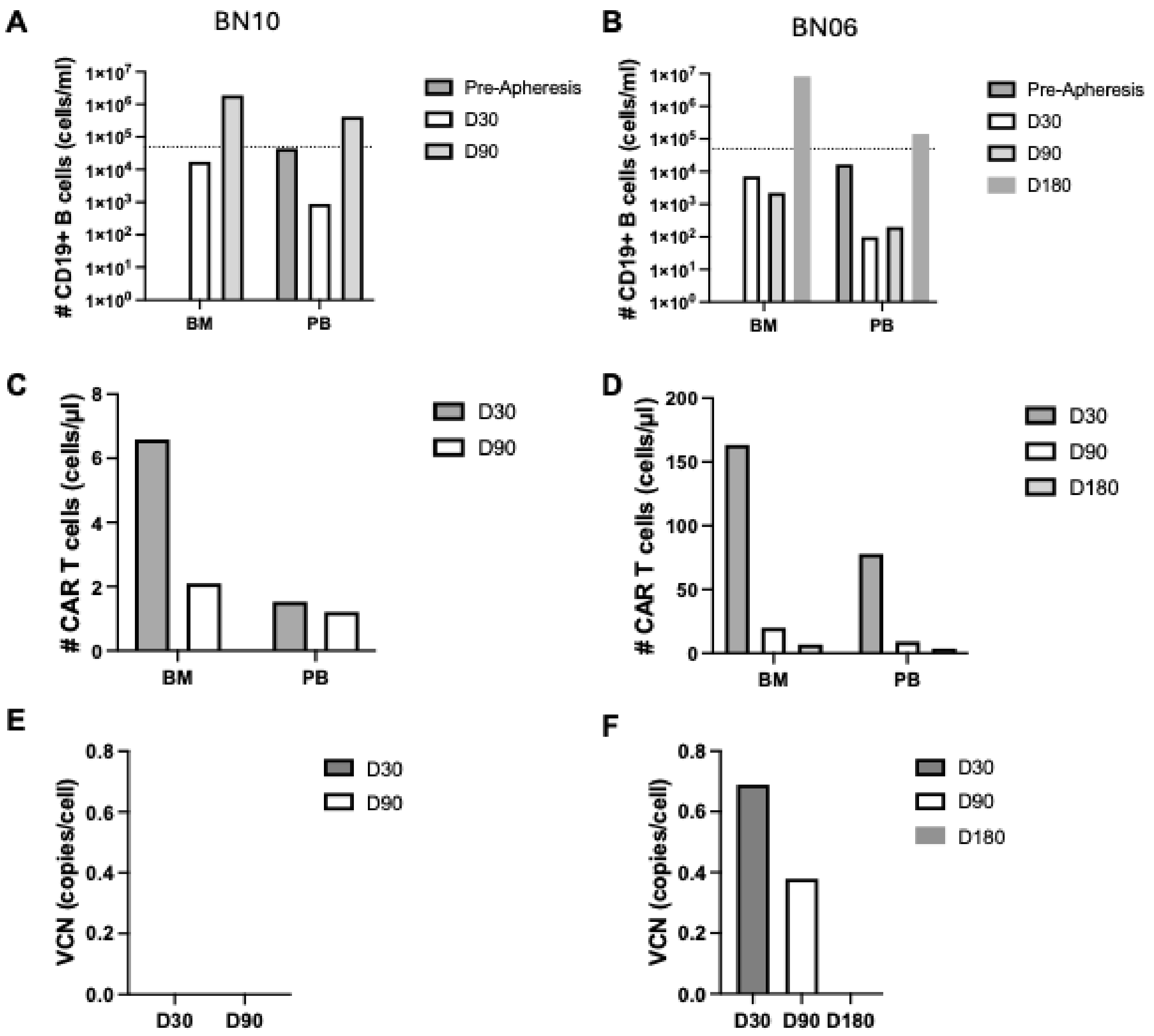

Figure S4: CD19^{+} B cell and CAR T-cell levels in patients with B cell recovery. (A, B) Flow cytometry analysis of the BM and PB from patients BN10 (A) and BN06 (B) at pre-apheresis (only available for PB), day 30, day 90, and day 180. **(C, D)** Quantification of CAR T-cell by flow cytometry in the BM and PB of patients BN10 (C) and BN06 (D) at day 30, day 90, and day 180. **(E, F)** Vector copy number (VCN) in the BM and PB of patients BN10 (E) and BN06 (F), measured by quantitative PCR at day 30, day 90, and day 180. Abbreviations: bone marrow (BM), complete remission (CR), day (D), peripheral blood (PB), polymerase chain reaction (PCR), and vector copy number (VCN).

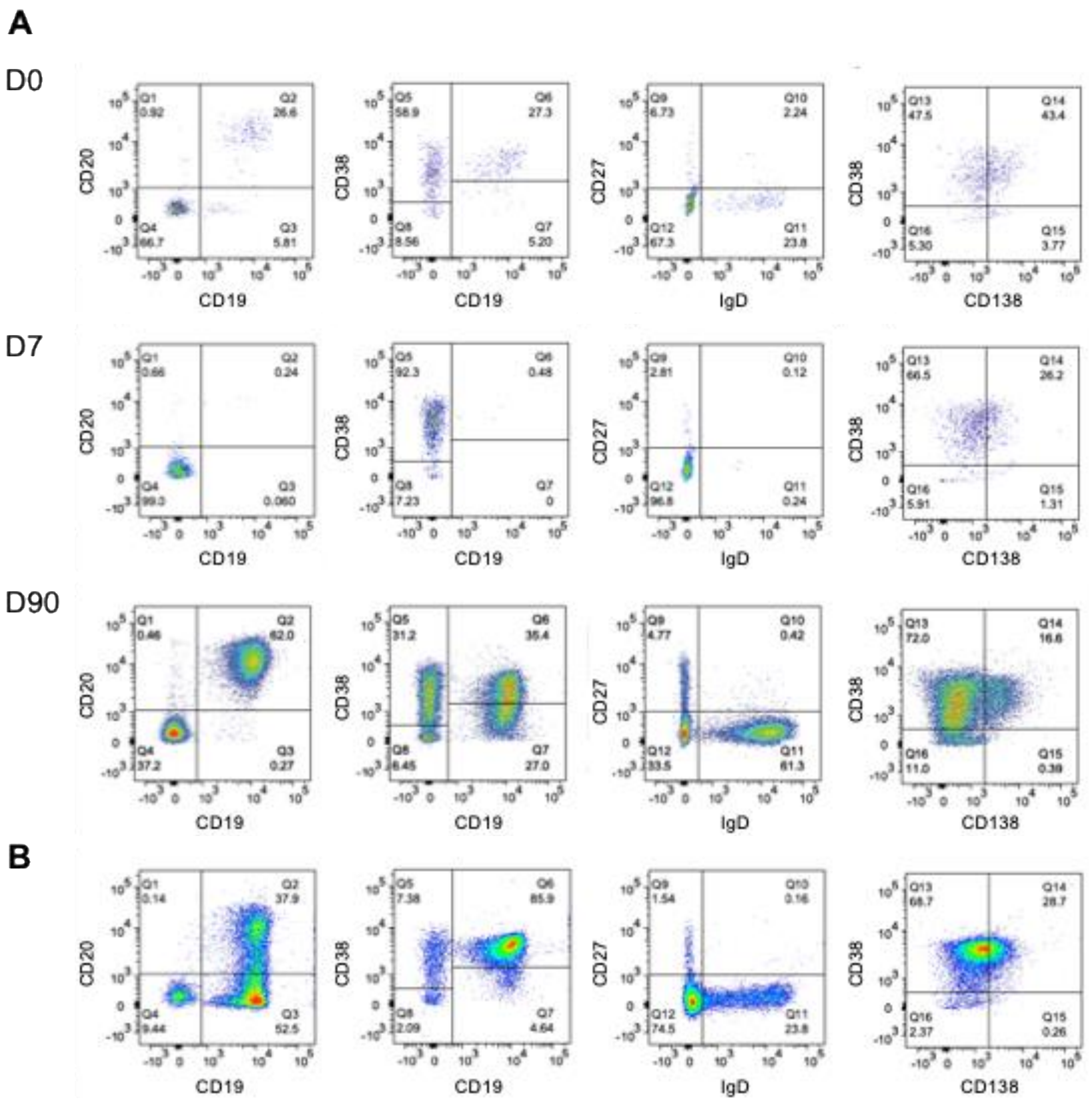

Figure S5: B-cell aplasia and hypogammaglobulinemia recovery in patients following CAR T-cell infusion. (A) B-cell recovery in patient BN10. On day 7 post-infusion, the patient exhibited B-cell aplasia. By day 90, B-cells had recovered in the PB, displaying differentiation into CD19+CD20+CD38+ pre-B cells, CD19+CD20+IgD+ activated B cells, CD19+CD20+CD27+ memory B cells, and CD38+CD138+ plasma cells. **(B)** BM sample from day 90 revealed a similar differentiation pattern, but with a higher frequency of immature CD19+CD20−CD38+ pro-B cells. Abbreviations: bone marrow (BM), day (D), peripheral blood (PB).

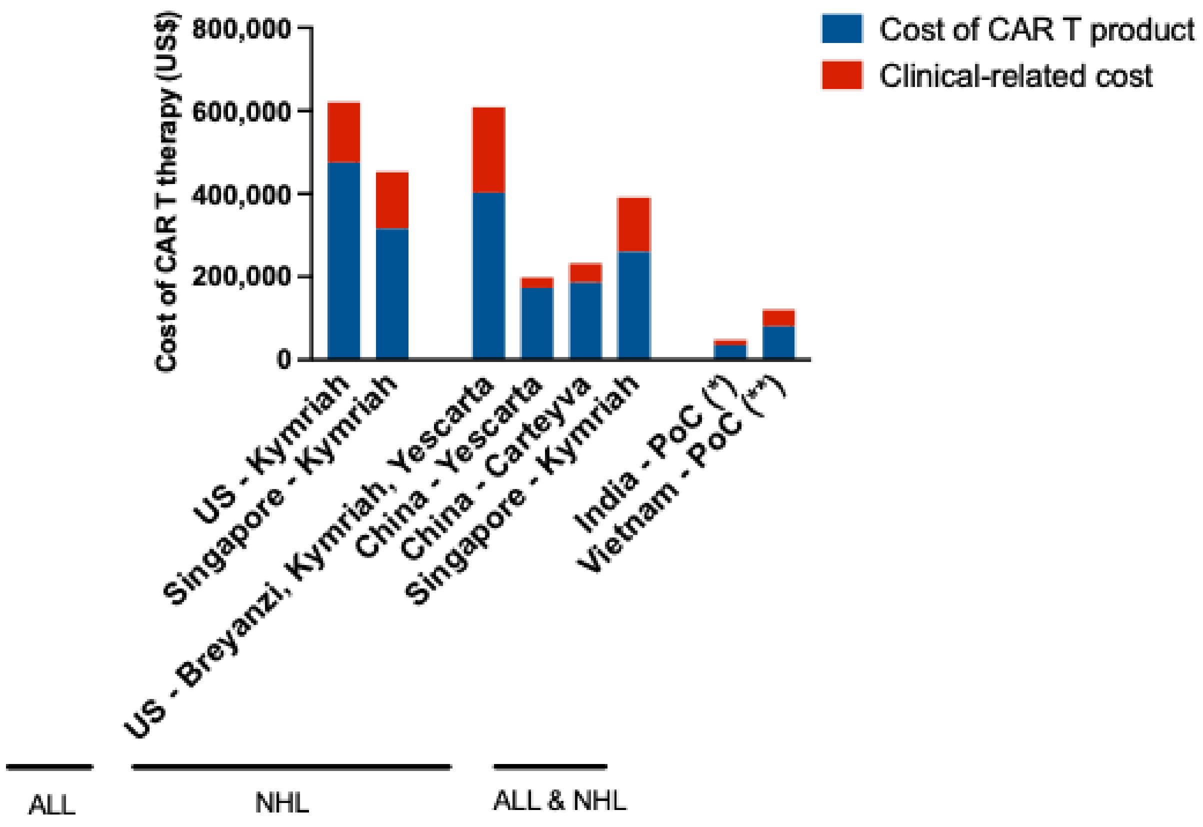

Figure S6: CAR T therapy cost for ALL and NHL. (*) Exclusive cost of vector; (**) Inclusive cost of vector. Abbreviations: PoC, Point of Care.

Table S1. Disease burden and outcomes of individual patients

Patient ID	Age	Tumor size (mm x mm)	BM blast before treatment	CAR T-cell peak (cells/µl)	Follow-up duration (months)	Response at day 30	Response at day 90	Response at day 180
**ALL**								
BN01	8	NA	5%	528.9	20.9	CR	CR	CR
BN03	12	NA	10%	331.9	17.0	CR	Relapse	Relapse
BN06	5	NA	0%	989.4	17.9	CR	CR	CR
BN07	16	NA	4%	1125.9	3.5	CR	Relapse	Death
BN09	24	NA	0%	1680.6	14.5	CR	CR	CR
BN10	11	NA	0%	54.0	14.2	CR	CR	CR
BN12	50	NA	0%	788.9	12.8	CR	CR	Relapse
BN17	27	NA	0%	280.5	7.2	CR	CR	CR
**NHL**								
BN05	35	117 x 81	NA	6529.3	7.4	NA	No response	No response
BN08	55	Nil	NA	260.2	16.3	NA	CR	CR
BN11	32	Nil	NA	2194.3	13.5	NA	CR	CR
BN13	38	Nil	NA	142.5	11.9	NA	CR	CR
BN14	40	8x19	NA	82.9	10.6	NA	CR	CR
BN15	45	12x15	NA	2277.5	9.8	NA	CR	CR

BN16	50	Nil	NA	1825.2	7.9	NA	CR	CR
BN18	42	Nil	NA	731.3	6.1	NA	CR	CR

Abbreviations: ALL, acute lymphoblastic leukemia; BM, bone marrow; CR, complete remission; NA, not applicable; NHL, non-Hogdkin lymphoma.

Table S2. Baseline characteristics and pharmacodynamic metrics stratified by clinical response

**Clinical Parameters**	**CR at day 180 (the CR group)**	**Non-responders/relapsed patients (the R/R group)**	**p value**	**p adj BH**
**Baseline characteristics**				
Age (years)	35.0 [20.8, 42.8]	25.5 [15.0, 38.8]	0.903	1.000
Serum LDH (U/L)	202.5 [158.8, 272.2]	277.0 [209.8, 618.8]	0.203	0.568
Female sex, n (%)	8 (66.7%)	1 (25.0%)	0.262	0.610
High disease burden (ALL BM blast ≥ 5%; NHL: bulky lymphoma)	1 (8.3%)	2 (50.0%)	0.136	0.475
**Pharmacodynamic metrics**				
Total CAR T-cell dose	1.8 [1.7, 2.0]	2.0 [1.7, 2.1]	0.504	0.785
Peak CAR T-cells (cells/μL)	630.1 [230.8, 1716.7]	957.4 [674.6, 2476.8]	0.363	0.726
Peak IL-6 (pg/mL)	35.3 [22.1, 589.8]	5987.5 [4372.0, 6312.5]	0.06	0.421

Other baseline laboratory parameters				
White blood cells (G/L)	5.1 [3.1, 5.7]	4.4 [2.8, 5.9]	1	1.000
Lymphocytes (G/L)	1.1 [1.0, 1.4]	0.8 [0.7, 0.8]	0.029	0.403
Platelets (G/L)	187.0 [150.5, 238.0]	227.0 [171.2, 288.0]	0.504	0.785
Total bilirubin (µmol/L)	8.7 [7.3, 10.2]	11.4 [10.3, 13.6]	0.129	0.475
SGOT (U/L)	27.2 [23.2, 31.3]	29.4 [22.2, 39.5]	0.952	1.000
SGPT (U/L)	22.5 [14.7, 33.5]	25.1 [17.8, 36.4]	0.671	0.939
Creatinine (µmol/L)	50.0 [42.0, 78.8]	50.5 [42.0, 62.2]	0.808	1.000

Notes: Continuous variables are median [IQR] and were compared using the Mann–Whitney U (Wilcoxon rank-sum) test. Categorical variables are n (%) and were compared using Fisher's exact test. Two-sided p values are reported; Benjamini–Hochberg adjusted p values are provided for reference. Patient level disease burden measures are summarized in Table S1.

Abbreviations: BH, Benjamini–Hochberg; CAR T, chimeric antigen receptor T-cells; IL-6, interleukin 6; LDH, lactate dehydrogenase.

Table S3. Costs of CAR T-cell therapy across commercial and point-of-care manufacturing settings.

Due to its size, this table is provided as a separate Excel file.

Table S4. CRS and ICANS of CAR T-cell therapy

	CRS		ICANS		ICU requirement	Early Death
Author	Total number of CRS (%)	CRS ≥ 3 *level* (%)	Total number of ICANS (%)	ICANS ≥3 (%)		
Dourthe M-E et al. (1)	59	20	24	8	35	1
Kochenderfer JN	NA	26.7	33.3	NA	NA	1
Palani HK et al. (2)	90	10	Nil	Nil	No	No
Locke FL et al. (3)	92	11	67	33	0	
Schuster SJ* et al. (4)	58	22	20	11	24	3
Abramson JS et al. ** (5)	42	2	30	10	4	
**Liem NT et al.**	**81.3**	**6.3**	**12.5**	**0**	**1**	**No**

*The JULIET study employed a grading scale and management guideline developed by researchers at the University of Pennsylvania, while the ZUMA-1.

**TRANSCEND trials adopted a version formulated by a panel of experts convened by the National Cancer Institute and later published by Lee et al (6).

Abbreviations: CRS, cytokine release syndrome; ICANS, immune effector cell-associated neurotoxicity syndrome; ICU, intensive care unit.

Table S5. Outcomes of CAR T-cell therapy

Authors	ORR (%)	CR (%)	Median PFS, mo (95% CI)	Median OS, mo (95% CI)	Median follow-up duration, mo
ALL					
Maude SL et al. (9)	81	81[a]	–	76 (63 – 86)	13.1
**Liem NT et al.**	**100**	**100**	**NE (1-year PFS rate of 62.5%)**	**NE (1-year OS rate of 75%)**	**10.4**
NHL					
Locke FL et al. (3)	83	58	5.9 (3.3–15.0)	NR (12.8-NE)	27.1
Schuster SJ et al. (7)	53	39	2.9 (2.3–5.2)	11.1 (6.6–23.9)	40.3
Abramson JS et al. (8)	73	53	6.8 (3.3-12.7)	27.3 (16.2-45.6)	19.9
**Liem NT et al.**	**87.5**	**87.5**	**NE (1-year PFS rate of 87.5%)**	**NE (1-year OS rate of 87.5%)**	**12.9**

Abbreviations: CRR, complete remission rate; mo, month; NE, not estimable; NR, not reached; ORR: overall response rate; OS, overall survival; PFS, progression-free survival.

[a] 60% of patients achieved CR and 21% of patients achieved CR with incomplete blood count recovery (CRi).

References

1. Dourthe M-E, Rabian F, Yakouben K, Chevillon F, Cabannes-Hamy A, Méchinaud F, Grain A, Chaillou D, Rahal I, Caillat-Zucman S, Lesprit E, Naudin J, Roupret-Serzec J, Parquet N, Brignier A, Guérin-El Khourouj V, Lainey E, Caye-Eude A, Cavé H, Clappier E, Mathis S, Azoulay E, Dalle JH, Dhédin N, Madelaine I, Larghero J, Boissel N, Baruchel A. Determinants of CD19-positive vs CD19-negative relapse after tisagenlecleucel for B-cell acute lymphoblastic leukemia. Leukemia. 2021 2021/12/01;35(12):3383-3393. doi:10.1038/s41375-021-01281-7.

2. Palani HK, Arunachalam AK, Kulkarni U, Yasar M, Venkatraman A, Palanikumar S, Radhakrishnan RN, Solomon M, Rajasekaran A, Bankar A, Datari PVR, Selvarajan S, Korula A, Dash P, Schneider D, Wirthlin L, Abraham A, George B, Mathews V. Safety, efficacy and total cost of point-of-care manufactured anti-CD19 CAR-T cell therapy in India: VELCART trial. Molecular Therapy Oncology. 2025;33(2). doi:10.1016/j.omton.2025.200977.

3. Locke FL, Ghobadi A, Jacobson CA, Miklos DB, Lekakis LJ, Oluwole OO, Lin Y, Braunschweig I, Hill BT, Timmerman JM, Deol A, Reagan PM, Stiff P, Flinn IW, Farooq U, Goy A, McSweeney PA, Munoz J, Siddiqi T, Chavez JC, Herrera AF, Bartlett NL, Wiezorek JS, Navale L, Xue A, Jiang Y, Bot A, Rossi JM, Kim JJ, Go WY, Neelapu SS. Long-term safety and activity of axicabtagene ciloleucel in refractory large B-cell lymphoma (ZUMA-1): a single-arm, multicentre, phase 1-2 trial. Lancet Oncol. 2019 Jan;20(1):31-42. eng. Declaration of interests FLL has served on scientific advisory boards for Kite and Novartis and reports consultancy fees for Cellular Biomedicine and research support from Forma Therapeutics. AGh has received speaker fees and research funding from, and served on scientific advisory boards for, Kite. CAJ reports personal fees from Kite, Novartis, Precision Bioscience, Bayer, Pfizer, and Humanigen. DBM has received grants and research funding from, and served on scientific advisory boards for, Kite. IB reports speaker fees from Kite.

BTH has served on advisory boards for Gilead. AD has served on advisory boards for Kite, Agios, and Novarits, and received research funding from Bristol-Myers Squibb. PMR reports research funding from Seattle Genetics, and has served on advisory boards for, and received personal fees from, Curis. IWF reports research funding from Kite, Agios, ArQule, Beigene, Calithera, Celgene, Constellation, Curis, Forma, Forty-Seven, Genentech, Gilead, Incyte, Infinity, Janssen, Merck, Novartis, Pfizer, Pharmacyclics, Portola, Seattle Genetics, Takeda, TG Therapeutics, Trillium, Verastem, and Roche. UF reports travel support from Kite and honoraria from Celgene. AGo has participated in speakers bureaus for, and received research funding from, Takeda, Kite, Gilead, Pharmacyclics, Janssen, Genentech, and Acerta, and has a leadership role at COTA, a for-profit health-care company. PAM reports speaker and consultant fees from Kite. JM has participated on speakers bureaus or advisory boards for Kite, Pharmacyclics, Janssen, Bayer, Alexion, Pfizer, Juno, Celgene, Bristol-Myers Squibb, Genentech, and Kyowa. TS reports travel support from Kite, speaker and consultancy fees from Pharmacyclics; speaker fees from Janssen and Seattle Genetics, and consultancy for Juno and BeiGene. JCC has participated in advisory boards or speakers bureaus for Kite, Genentech, Novartis, Bayer, and Janssen, and has received research support from Merck. AFH reports grants, personal fees, and consultancy for Bristol-Myers Squibb, Genentech, Merck, Pharamcyclics, and Kite, and grants and consultancy for Gilead. NLB reports research funding from Affimed, Bristol-Meyers Squibb, Celgene, Forty Seven, Genentech, Gilead, Immune Design, Kite, Merck, Millennium, Pharmacyclics, and Acerta, and has served on advisory boards for Pfizer. LN, JMR, AX, YJ, JJK, and WYG, are employed by Kite, and have equity ownership in Gilead. AB and JSW report employment and an issued patent with Kite. SSN reports personal fees and research support from Kite, Merck, and Celgene, research support from Bristol-Myers Sqibb, Poseida, Cellectis, Karus, and Acerta Pharma, and personal fees from Novartis, Pfizer, and Unum Therapeutics. LJL, OOO, YL, JMT, and PS declare no

competing interests. Epub 20181202. doi:10.1016/s1470-2045(18)30864-7. Cited in: Pubmed; PMID 30518502.

4. Schuster SJ, Bishop MR, Tam CS, Waller EK, Borchmann P, McGuirk JP, Jäger U, Jaglowski S, Andreadis C, Westin JR, Fleury I, Bachanova V, Foley SR, Ho PJ, Mielke S, Magenau JM, Holte H, Pantano S, Pacaud LB, Awasthi R, Chu J, Anak Ö, Salles G, Maziarz RT. Tisagenlecleucel in Adult Relapsed or Refractory Diffuse Large B-Cell Lymphoma. N Engl J Med. 2019 Jan 3;380(1):45-56. eng. Epub 20181201. doi:10.1056/NEJMoa1804980. Cited in: Pubmed; PMID 30501490.

5. Abramson JS, Palomba ML, Gordon LI, Lunning MA, Wang M, Arnason J, Mehta A, Purev E, Maloney DG, Andreadis C, Sehgal A, Solomon SR, Ghosh N, Albertson TM, Garcia J, Kostic A, Mallaney M, Ogasawara K, Newhall K, Kim Y, Li D, Siddiqi T. Lisocabtagene maraleucel for patients with relapsed or refractory large B-cell lymphomas (TRANSCEND NHL 001): a multicentre seamless design study. Lancet. 2020 Sep 19;396(10254):839-852. eng. Epub 20200901. doi:10.1016/s0140-6736(20)31366-0. Cited in: Pubmed; PMID 32888407.

6. Lee DW, Gardner R, Porter DL, Louis CU, Ahmed N, Jensen M, Grupp SA, Mackall CL. Current concepts in the diagnosis and management of cytokine release syndrome. Blood. 2014 Jul 10;124(2):188-95. eng. Epub 20140529. doi:10.1182/blood-2014-05-552729. Cited in: Pubmed; PMID 24876563.

7. Schuster SJ, Tam CS, Borchmann P, Worel N, McGuirk JP, Holte H, Waller EK, Jaglowski S, Bishop MR, Damon LE, Foley SR, Westin JR, Fleury I, Ho PJ, Mielke S, Teshima T, Janakiram M, Hsu J-M, Izutsu K, Kersten MJ, Ghosh M, Wagner-Johnston N, Kato K, Corradini P, Martinez-Prieto M, Han X, Tiwari R, Salles G, Maziarz RT. Long-term clinical outcomes of tisagenlecleucel in patients with relapsed or refractory aggressive B-cell

lymphomas (JULIET): a multicentre, open-label, single-arm, phase 2 study. The Lancet Oncology. 2021;22(10):1403-1415. doi:10.1016/S1470-2045(21)00375-2.

8. Abramson JS, Palomba ML, Gordon LI, Lunning MA, Wang M, Arnason JE, Purev E, Maloney DG, Andreadis C, Sehgal AR, Solomon SR, Ghosh N, Kostic A, Kim Y, Ogasawara K, Dehner C, Siddiqi T. Two-Year Follow-up of Transcend NHL 001, a Multicenter Phase 1 Study of Lisocabtagene Maraleucel (liso-cel) in Relapsed or Refractory (R/R) Large B-Cell Lymphomas (LBCL). Blood. 2021;138(Supplement 1):2840-2840. doi:10.1182/blood-2021-148948.

9. Maude SL, Laetsch TW, Buechner J, Rives S, Boyer M, Bittencourt H, Bader P, Verneris MR, Stefanski HE, Myers GD, Qayed M, Moerloose BD, Hiramatsu H, Schlis K, Davis KL, Martin PL, Nemecek ER, Yanik GA, Peters C, Baruchel A, Boissel N, Mechinaud F, Balduzzi A, Krueger J, June CH, Levine BL, Wood P, Taran T, Leung M, Mueller KT, Zhang Y, Sen K, Lebwohl D, Pulsipher MA, Grupp SA. Tisagenlecleucel in Children and Young Adults with B-Cell Lymphoblastic Leukemia. New England Journal of Medicine. 2018;378(5):439-448. doi:doi:10.1056/NEJMoa1709866.

10. Hoover A, Reimche P, Watson D, Tanner L, Gilchrist L, Finch M, Messinger YH, Turcotte LM. Healthcare cost and utilization for chimeric antigen receptor (CAR) T-cell therapy in the treatment of pediatric acute lymphoblastic leukemia: A commercial insurance claims database analysis. Cancer Rep (Hoboken). 2024 Feb;7(2):e1980. The authors have no conflicts of interest to disclose. Epub 20240113. doi:10.1002/cnr2.1980. Cited in: Pubmed; PMID 38217445.

11. Di M, Potnis KC, Long JB, Isufi I, Foss F, Seropian S, Gross CP, Huntington SF. Costs of care during chimeric antigen receptor T-cell therapy in relapsed or refractory B-cell lymphomas. JNCI Cancer Spectr. 2024 Jul 1;8(4). K.C.P., J.B.L., and S.S. do not have any

conflict of interest to report. M.D. reports an honorarium from Intellisphere, consultancy fee from BeiGene, and research funding from Schrodinger and BeiGene. I.I. reports membership on an entity's board of directors or advisory committees at ADC Therapeutics, BEAM Therapeutics, and Epizyme; honoraria from Bayer; and speaker bureau from Kite. F.F. reports consultancy fees from Kyowa, Conjupro, Daiichi, Seagen, and Astex and speaker bureau from Seagen. C.P.G. reports research funding from the National Comprehensive Cancer Network Foundation (AstraZeneca), Genentech, and Johnson & Johnson. S.F.H. reports consultancy fees from Janssen, Pharmacyclics, AbbVie, AstraZeneca, Flatiron Health, Novartis, Seagen, Genentech, Merck, TG Therapeutics, ADC Therapeutics, Epizyme, Servier, and Thyme; research funding from Celgene, DTRM Biopharm, and TG Therapeutics; and honoraria form Pharmacyclics, AstraZeneca, and Bayer. doi:10.1093/jncics/pkae059. Cited in: Pubmed; PMID 39115391.

12. Wu J, Ghobadi A, Maziarz R, Patel K, Hsu H, Liu Z, Sheetz C, Kardel P, Fu C. Medicare Utilization and Cost Trends for CAR T Cell Therapies Across Settings of Care in the Treatment of Diffuse Large B-Cell Lymphoma. Adv Ther. 2024 Aug;41(8):3232-3246. Hill Hsu is employee of Kite Pharma, A Gilead Company and Christine Fu and James Wu are former employees of Kite Pharma, A Gilead Company. Zihao Liu, Peter Kardel, and Caitlin Sheetz are employees of ADVI Health Inc. Richard Maziarz reports serving as consultant for Autolous, Kite/Gilead, and Novartis, research support from Gamida, Allovir, OrcaBio, Kite/Gilead and Novartis, participating in a DSMB for Athersys, Novartis, Century Therapeutics and VorPharma and a patent with Athersys; no activity has conflict with the material of this article. Armin Ghobadi serving as consultant/advisory board for WUGEN, Atara pharmaceuticals, Celgene/BMS, CRISPR Therapeutics, Autolus, ADC Therapeutics, Nurix Therapeutics, serving as consultant and provide research support for Kite, a Gilead Company and Amgen Inc and Genentech. Krish Patel serve as consultant or received research

funding from AstraZeneca, Beigene, BMS, Loxo, Gentech/Roche, Kite, Century, CRISPR, Caribou, Curis, Sana, Fate Therapeutics, Xencor, ADC, Janssen, Nurix, Pfizer, Pharmacyclics. Epub 20240625. doi:10.1007/s12325-024-02917-7. Cited in: Pubmed; PMID 38916811.

13. Masucci L, Kuruvilla J, Sander B, Prica A, Wong WWL, Chan KKW. Real-world healthcare costs for patients treated with chimeric antigen receptor T-cell therapy in Canada. Leuk Lymphoma. 2025 Jun 17:1-9. Epub 20250617. doi:10.1080/10428194.2025.2518440. Cited in: Pubmed; PMID 40525245.

14. Heine R, Thielen FW, Koopmanschap M, Kersten MJ, Einsele H, Jaeger U, Sonneveld P, Sierra J, Smand C, Uyl-de Groot CA. Health Economic Aspects of Chimeric Antigen Receptor T-cell Therapies for Hematological Cancers: Present and Future. Hemasphere. 2021 Feb;5(2):e524. The authors have no conflicts of interest to disclose. Epub 20210128. doi:10.1097/HS9.0000000000000524. Cited in: Pubmed; PMID 33880433.

15. Ribera Santasusana JM, de Andres Saldana A, Garcia-Munoz N, Gostkorzewicz J, Martinez Llinas D, Diaz de Heredia C. Cost-Effectiveness Analysis of Tisagenlecleucel in the Treatment of Relapsed or Refractory B-Cell Acute Lymphoblastic Leukaemia in Children and Young Adults in Spain. Clinicoecon Outcomes Res. 2020;12:253-264. JMRS and CDH are, respectively, employed by Catalan Institute of Oncology-Hospital Germans Trias i Pujol and Hospital Universitari Vall d'Hebron. DML and NGM are employees of Oblikue Consulting, an independent contract health economic organization that received consultancy fees from Novartis Farmaceutica, S.A. to conduct this research. AAS and JG are employees of Novartis Farmaceutica, S.A., the marketing authorization holder for Kymriah(R) (tisagenlecleucel). JMRS reports grants and personal fees from AMGEN, Pfizer, Incyte, and Shire, and personal fees from Celgene, outside the submitted work. CHD reports personal fees and non-financial support from Novartis during the conduct of the study; and personal fees and non-financial support from Jazz Pharmaceuticals, Gilead, and Novartis, and non-financial support from

Alexion, outside the submitted work. The funding body was not involved in the study design, collection and interpretation of the data, or the decision to publish. The authors report no other conflicts of interest in this work. Epub 20200515. doi:10.2147/CEOR.S241880. Cited in: Pubmed; PMID 32523362.

16. Bastos-Oreiro M, de Las Heras A, Presa M, Casado MA, Pardo C, Martin-Escudero V, Sureda A. Cost-Effectiveness Analysis of Axicabtagene Ciloleucel vs. Tisagenlecleucel for the Management of Relapsed/Refractory Diffuse Large B-Cell Lymphoma in Spain. Cancers (Basel). 2022 Jan 21;14(3). Bastos-Oreiro M. has received conference and consulting fees from BMS, Celgene, Kite Pharma, Novartis, Roche and Takeda. Sureda A. has received conference and consulting fees from BMS, Celgene, Gilead, Janssen, MSD, Novartis, Roche, Sanofi and Takeda. Bastos-Oreiro B. and Sureda A. have received honoraria from Gilead for advocacy tasks related to this project. de las Heras A., Presa M., and Casado MA. are employees of Pharmacoeconomics & Outcomes Research Iberia, a consultant company specialised in economic evaluation of health technologies which has received unrestricted funding for development of the analysis. Pardo C. and Martin-Escudero V. are employees of Gilead Sciences Spain. Epub 20220121. doi:10.3390/cancers14030538. Cited in: Pubmed; PMID 35158805.

17. Li N, Zheng B, Cai H, Yang T, Hong Y, Liu M, Hu J. Cost-effectiveness analysis of axicabtagene ciloleucel vs. salvage chemotherapy for relapsed or refractory adult diffuse large B-cell lymphoma in China. Support Care Cancer. 2022 Jul;30(7):6113-6121. Epub 20220414. doi:10.1007/s00520-022-07041-2. Cited in: Pubmed; PMID 35419735.

18. Lin Z, Zuo C, Jiang Y, Su W, Yao X, Man Y, Wu Q, Xuan J. Cost-Effectiveness Analysis of Relmacabtagene Autoleucel for Relapsed or Refractory Large B-Cell Lymphoma in China. Value Health Reg Issues. 2023 Sep;37:41-48. Epub 20230518. doi:10.1016/j.vhri.2023.03.006. Cited in: Pubmed; PMID 37209540.

19. Wang XJ, Wang YH, Ong MJC, Gkitzia C, Soh SY, Hwang WYK. Cost-Effectiveness and Budget Impact Analyses of Tisagenlecleucel in Pediatric and Young Adult Patients with Relapsed or Refractory B-Cell Acute Lymphoblastic Leukemia from the Singapore Healthcare System Perspective. Clinicoecon Outcomes Res. 2022;14:333-355. XJW, YHW, MJCO, and CG are employees of Novartis. WYKH has received personal fees as Advisory Board member from Novartis and Gilead, and grant from CordLife for a clinical study. SYS has no financial or other interests for declaration here. The authors report no other conflicts of interest in this work. Epub 20220503. doi:10.2147/CEOR.S355557. Cited in: Pubmed; PMID 35535300.

20. Mallapaty S. Cutting-edge CAR-T cancer therapy is now made in India - at one-tenth the cost. Nature. 2024 Mar;627(8005):709-710. doi:10.1038/d41586-024-00809-y. Cited in: Pubmed; PMID 38514877.

21. Cliff ERS, Kelkar AH, Russler-Germain DA, Tessema FA, Raymakers AJN, Feldman WB, Kesselheim AS. High Cost of Chimeric Antigen Receptor T-Cells: Challenges and Solutions. Am Soc Clin Oncol Educ Book. 2023 Jun;43:e397912. doi:10.1200/EDBK_397912. Cited in: Pubmed; PMID 37433102.

22. Ran T, Eichmuller SB, Schmidt P, Schlander M. Cost of decentralized CAR T-cell production in an academic nonprofit setting. Int J Cancer. 2020 Dec 15;147(12):3438-3445. Epub 20200706. doi:10.1002/ijc.33156. Cited in: Pubmed; PMID 32535920.

23. Palani HK, Arunachalam AK, Kulkarni U, Yasar M, Venkatraman A, Palanikumar S, Radhakrishnan RN, Solomon M, Rajasekaran A, Bankar A, Datari PVR, Selvarajan S, Korula A, Dash P, Schneider D, Wirthlin L, Abraham A, George B, Mathews V. Safety, efficacy and total cost of point-of-care manufactured anti-CD19 CAR-T cell therapy in India: VELCART trial. Mol Ther Oncol. 2025 Jun 18;33(2):200977. The authors declare no competing interests. Epub 20250325. doi:10.1016/j.omton.2025.200977. Cited in: Pubmed; PMID 40248244.
